# Tobacco smoking and risks of >470 diseases in China: a prospective cohort study

**DOI:** 10.1016/S2468-2667(22)00227-4

**Published:** 2022-12

**Authors:** Ka Hung Chan, Neil Wright, Dan Xiao, Yu Guo, Yiping Chen, Huaidong Du, Ling Yang, Iona Y. Millwood, Pei Pei, Junzheng Wang, Iain Turnbull, Simon Gilbert, Daniel Avery, Christiana Kartsonaki, Canqing Yu, Junshi Chen, Jun Lv, Robert Clarke, Rory Collins, Richard Peto, Liming Li, Chen Wang, Zhengming Chen

**Affiliations:** 1 Clinical Trial Service Unit and Epidemiological Studies Unit, Nuffield Department of Population Health, University of Oxford; 2 Oxford British Heart Foundation Centre of Research Excellence, University of Oxford; 3 WHO Collaborating Center for Tobacco Cessation and Respiratory Diseases Prevention, China-Japan Friendship Hospital, Beijing, China; 4 Institute of Respiratory Medicine, Chinese Academy of Medical Sciences, Beijing, China; 5 National Center for Cardiovascular Disease Fuwai Hospital, Chinese Academy of Medical Science, Beijing, China; 6 MRC Population Health Research Unit, Nuffield Department of Population Health, University of Oxford; 7 Peking University Center for Public Health and Epidemic Preparedness & Response; 8 Licang Center of Disease Control and Prevention, Qingdao, China; 9 Department of Epidemiology and Biostatistics, School of Public Health, Peking University, Beijing, China; 10 China National Center for Food Safety Risk Assessment, Beijing, China

## Abstract

**Background:**

Tobacco smoking is estimated to account for >1 million annual deaths in China, and the epidemic continues to increase in men. Large nationwide prospective studies linked to different health records can help assess periodically disease burden attributed to smoking. We examined associations of smoking with an extensive range of disease incidence and mortality in China.

**Methods:**

The prospective China Kadoorie Biobank recruited >512,000 adults aged 30-79 years from 10 diverse areas during 2004-2008, and recorded detailed smoking information. During 12-year follow-up, 1,137,603 ICD-10 coded hospitalisation events and 48,760 deaths were recorded, involving 476 and 85 distinct conditions, each with ≥100 incident cases and deaths, respectively. Cox regression yielded adjusted hazard ratios (HRs) associating smoking with disease outcomes, adjusting for multiple-testing.

**Findings:**

At baseline 67·7% of men and 3·2% of women (overall 29·4%) ever smoked regularly. Compared with never-smokers, ever-smokers had significantly higher risks for 9 of 18 ICD-10 disease chapters examined at ages 35-84 years. For individual conditions, smokers had significantly higher risks of 56 diseases (men 50; women 24) and 22 causes of death (men 17; women 9). Among men, ever-smokers had HR of 1·09 (95% CI 1·08-1·11) for any disease incidence, and also significantly more episodes and longer duration of hospitalisation, particularly those due to cancer and respiratory diseases. For overall mortality, the HRs were greater in urban than in rural men (1·50 [1·43-1·58] vs. 1·25 [1·20-1·30]). Among urban men who began smoking at age <18 years, the HRs were 2·06 (1·89-2·24) for overall mortality and 1·32 (1·27-1·37) for any disease incidence. In this population, 20% of male (urban 24%; rural 16%) and 3% of female deaths were attributed to ever-regular smoking.

**Interpretation:**

Among Chinese adults, smoking was associated with higher risks of morbidity and mortality from a wide range of diseases. Among men, the future smoking-attributed disease burden will increase further, highlighting a pressing need for reducing consumption, through widespread cessation and uptake prevention.

## Introduction

Worldwide smoking accounted for about 100 million deaths in the 20^th^ century and is projected to cause one billion deaths this century, mainly in low- and middle-income countries (LMICs), including China.^1^ China now consumes about 40% of the world’s tobacco, almost exclusively by men.^2^ In China the main increase in consumption of manufactured cigarette took place after 1980 and continued until the 2010s,^2^ many decades behind that in high-income Western countries.^1,3–5^ Previous nationwide cohort studies established decades apart in China have reliably demonstrated the increasing proportion of adult mortality attributed to smoking in men.^6^ However, the morbidity burden attributed to smoking from a much broader range of diseases has not been properly studied in China (and most other LMICs).

There is compelling evidence that smoking causes multiple diseases, chiefly cancer, cardiovascular disease (CVD) and chronic respiratory diseases.^5^ Nevertheless, more recent large prospective studies in high-income Western countries have also identified new diseases associated with smoking, including chronic kidney disease, influenza, and mental disorders.^5,7–10^ In most LMICs, the available prospective evidence on smoking hazards was chiefly confined to mortality.^6,11–14^ In China, although many studies have attempted to assess the effects of smoking on morbidity outcomes, they were constrained by the use of non-prospective study designs, small number of outcomes involved (mainly site-specific cancer, chronic obstructive pulmonary disease [COPD] and CVD), restriction to specific urban cities, and by lack of objective validation of self-reported smoking status.^15–19^ Reliable assessment of smoking attributed morbidity, over and above mortality, burden in different populations is needed to inform effective tobacco control nationally and globally.

To help fill the evidence gap, we undertook detailed analyses of smoking and risks of hospitalisations and death in the prospective China Kadoorie Biobank (CKB) of >512,000 adults,^20,21^ involving 476 distinct diseases and 85 specific causes of death across all organ systems.

## Methods

### Study population

Details of the study design and baseline characteristics of CKB have been described elsewhere.^20,21^ Briefly, 512,726 adults aged 30-79 years were recruited via multi-stage cluster sampling in 2004-2008 from 10 (four urban, five rural, and one semi-rural) areas across China. These were selected through China’s nationally representative Disease Surveillance Point System^22^ to cover a diverse range of geographical areas, socioeconomic development, risk exposures and disease patterns. For each study site, about 100-150 administrative units (i.e. rural villages or urban street committees) were selected, and all eligible residents aged 35-74 years (n=1,801,167) were identified through local residential records and were invited to participate, of whom 499,439 (28%) participated, plus 13,287 individuals just outside the targeted age range (n=9817 at 30-34; n=3470 at 75-79 years), resulting in 512,726 participants at baseline age range of 30-79 years.

In local study assessment clinics, trained health workers undertook physical measurements (e.g. height, weight, and blood pressure) and a laptop-based questionnaire interview covering socio-demographic status, lifestyle, environmental factors, female reproductive factors and medical history.

Ethical approvals were obtained by the Ethical Review Committee of the China National Center for Disease Control and Prevention and the Oxford Tropical Research Ethics Committee, University of Oxford prior to commencement of the field work. All participants provided written informed consent.

### Assessment of smoking

The questionnaire on current and past smoking behaviours included age first began to smoke regularly, frequency, amount and type of tobacco smoked, degree of inhalation, and for ex-smokers, age last stopped and main reasons for cessation (due to illnesses or other reasons). To validate self-reported smoking status (along with exposure to household air pollution), exhaled carbon monoxide was also measured using MicroCO meters (Carefusion, San Diego, CA, USA).^23^ In the present study, participants who had never smoked or smoked <100 cigarettes in their lifetime were classified as “never-regular smokers” and those who smoked ≥1cigarette (or ≥1 gram tobacco) daily for at least 6 months were classified as “ever-regular smokers” (or “ever-smokers” for simplicity). Among participants who had stopped for ≥6 months, those who had stopped smoking due to illnesses were grouped with baseline current smokers as “regular smokers” as they would have significantly elevated disease risk even after stopping, while those who had stopped voluntarily for other reasons (e.g. finance) constituted a separate category to assess the effects of cessation (before ill health).^6^

### Follow-up for mortality and morbidity

Participants were followed up for death and any episodes of hospitalisation through electronic linkage via unique personal identification number to established mortality and morbidity (for cancer, stroke, ischaemic heart disease [IHD] and diabetes) registries and to national health insurance systems. All the reported disease events were coded following the International Classification of Diseases, 10^th^ Revision (ICD-10) by trained medical professionals blinded to baseline information. By 1 Jan 2018, 49,459 (9·6%) participants had died and 5302 (1·0%) were lost to follow-up. Participants were censored upon death, loss to follow-up, or 1 Jan 2018, whichever came first.

### Outcome measures

To enable a “phenome-wide” investigation, all disease events coded up to the first three characters of ICD-10 codes (i.e. the “disease category" component) were reviewed, and, where appropriate, combined (based on knowledge about the disease characteristics), to produce a list of distinct diseases. Several ICD-10 chapters considered irrelevant to the study population (e.g. perinatal-origin diseases [chapter XVI] and congenital conditions [XVII]) were excluded. Specific outcomes (or small number of specific disease groups) with at least 100 incident events (i.e. for those with multiple hospitalised events of same nature, only the first one was considered) or deaths (80 for sex-specific analyses) recorded during follow-up were analysed separately to capture a wide spectrum of conditions while ensuring reasonable statistical precision. Under each ICD-10 chapter, outcomes with <100 (<80 for sex-specific analyses) events were combined as “other disease” of the individual chapter to enable exploratory analysis that may shed light on rarer outcomes of the same disease system. Since the classification for the “other disease” endpoints were based on event number, the ICD-10 criteria used varies slightly in the combined and sex-specific analyses.

For overall mortality and major diseases constituting the top five causes of disability adjusted life years in China,^24^ namely IHD (ICD-10 I20-I25), intracerebral haemorrhage (ICH; I61), ischaemic stroke (IS; I63), COPD (J41-J44), and lung cancer (C34), further in-depth analyses by detailed smoking characteristics were also undertaken. The total number of hospitalisation episodes (i.e. including both first and subsequent events) and duration spent in hospitals (i.e. bed-days) attributed to all-cause, CVD, respiratory disease, cancer, and other causes were also examined as indicators of use of health service.

### Statistical analysis

We used Cox regression to estimate hazard ratios (HRs) comparing disease incidence or mortality risks at age-at-risk of 35-84 years in ever- (overall and by certain smoking characteristics) versus never-regular smokers, both overall and separately by sex (given the substantial sex-difference in smoking prevalence^6^). Ten participants died or were lost to follow-up before reaching 35 years of age, so a total of 512,712 participants were included in the analysis.

All analyses were stratified by age-at-risk (5-year groups), study areas (10 sites), and, where appropriate, sex and adjusted for education (no formal school; primary school; middle or high school; college or university) and alcohol drinking (never, occasional, or ever regular). Among men, analyses were also conducted separately in urban (four) and rural/semi-rural (six) areas. All-cause mortality is a competing risk for disease events and cause-specific mortality. Hence, to facilitate observational analyses assessing aetiological questions,^25^ participants were censored at death from any cause to estimate cause-specific HRs, which compared event rates in participants who were alive and free of the event of interest.

For smoking exposures involving more than two categories, group-specific CIs of HRs were calculated using the variance of the log hazard in each category, including the reference group. This enables direct comparison of HRs across any two categories of exposure, instead of just between a fixed reference group and other exposure categories.^26^ The proportional hazards assumption for ever-regular smoking was investigated by plotting and testing correlations between transformed follow-up time and the scaled Schoenfeld residuals^27^ for incidence and mortality of the top-five major diseases, among men and women.

For disease outcomes showing significant associations with smoking, sensitivity analyses were undertaken with additional adjustment. For top-five major diseases, we further excluded participants with prior medical history of relevant conditions at baseline (e.g. excluding those with prior CVD in analyses of IHD/stroke). Moreover, we also conducted separate analyses among current smokers and ex-smokers who had stopped due to illnesses, who together formed the “regular smoker” category.

To assess the cumulative burden of smoking, the total number of hospitalisations and days in hospital were estimated for ever- versus never-regular smokers using the mean cumulative count. This estimates the total number of events occurring in a population and does not assume independence between hospitalisations and all-cause mortality.^28–30^ Overall survival of regular versus never-regular smokers was also examined using Kaplan-Meier curves by sex. Adjusted incidence rates for individual diseases were calculated within age (<65, ≥65 years) and sex strata as

$$HR_{i}\times \frac{\text{overall}\;\text{rate }}{\frac{\sum^{+}\left(n_{i}\times HR_{i}\right)}{\sum^{+}n_{i}}}$$

where HR_i_ and n_i_ are hazard ratios and number of events for ever-regular smoker and never-smokers. Overall incidence rates were calculated as weighted means of the age- and sex-specific rates, and total incidence rates are the sum of disease-specific incidence rates. The fraction of all deaths that is attributed to smoking in the study population (the population-attributed fraction [PAF]) was estimated by *P*(*HR* – 1)/*HR*, where P is the prevalence of ever regular smoking among those dying during follow-up and HR is the associated hazard ratio for all-cause mortality.

Statistical significance (at the 5% level) was evaluated using both conventional and false discovery rate (FDR) adjusted p-values applied within ICD-10 chapters (and separately in the combined and sex-specific analyses),^31^ the latter of which controls the expected proportion of false positives among all significant associations.^32,33^ Unless otherwise specified, we only highlight associations that were statistically significant after FDR adjustment. All analyses were conducted using R software version 3·6·2.

### Role of the funding source

The study funders had no role in study design, data collection, analysis, interpretation, or writing of the report. KHC, NW, LL, and ZC had access to all data and had final responsibility for the decision to submit for publication.

## Results

Of the 512,716 participants included, 41·0% (n=210,201) were men, 66·3% (n=339,794) resided in rural areas, and the mean (SD) age was 52·0 (10·7) years at baseline. Overall, 29·4% (n= 150,801) smoked regularly, much higher in men (67·7%; n=142,205) than women (2·8%; n=8596). Both regular and ex-smokers tended to live in rural areas, to be less educated, more likely to consume alcohol and to have slightly lower BMI and higher prevalence of prior chronic diseases (eTable 1). Compared with female smokers, male smokers were more likely to start at a younger age, to be heavy smokers, and to habitually inhale tobacco smoke into the lungs. Among men, similar differences were also seen between younger and older smokers, with younger smokers more likely to start at age <20 and to smoke persistently manufactured cigarettes.

During 5·5 million person-year (median 12 years) of follow-up, 49,452 (9·6%) participants died and 285,888 (55·8%) were ever hospitalised at age-at-risk of 35-84 years. The “phenome-wide” investigation across 18 ICD-10 chapters included a total of 1,137,603 incident disease events from 476 different conditions, and 48,760 deaths from 85 causes (Table 1). Of the 18 ICD-10 disease chapters examined, ever-smoking was associated with significantly elevated risks in nine chapters (and no significant associations for the rest), including infectious and parasitic diseases (HR=1·07, 95%CI 1·03-1·11), neoplasms (1·34, 1·30-1·38), endocrine, nutritional and metabolic diseases (1·05, 1·02-1·09), circulatory diseases (1·10, 1·08-1·12), respiratory diseases (1·18, 1·16-1·21), digestive diseases (1·03, 1·01-1·06) and diseases of skin and subcutaneous tissue (1·14, 1·06-1·22) (eFigure 1). For the corresponding mortality analyses, the HRs were generally greater than the morbidity findings (eFigure 2). Although the HRs tended to be somewhat larger in men, the patterns of associations were broadly similar in men and women. Overall, male ever-smokers had 9% (1·09, 1·08-1·11) excess risk of morbidity from any diseases (eFigure 1), higher in urban than rural areas (1·17 [1·15-1·20] vs 1·05 [1·03-1·06]) (eTables 2 and 3). Among women ever-smokers, the HR for any morbidity was 1·04 (1·01-1·06) (eFigure 1).

Across the 476 distinct conditions investigated, ever-smoking was associated with statistically significant higher risks of 87 diseases (or aggregates of similar diseases), with 56 associations remaining significant after FDR adjustment, including 10 different types of circulatory diseases, 14 respiratory diseases, 14 cancers, and 5 digestive diseases (Table 1). In men, 50 different diseases were significantly positively associated (FDR-adjusted) with smoking (eTable 4), while among women, there were 24 such diseases (eTable 5). In contrast, seven conditions showed significant inverse associations with smoking in sex-combined analyses (eight in men, none in women). No significant association was found with other conditions.

**Figure 1:**
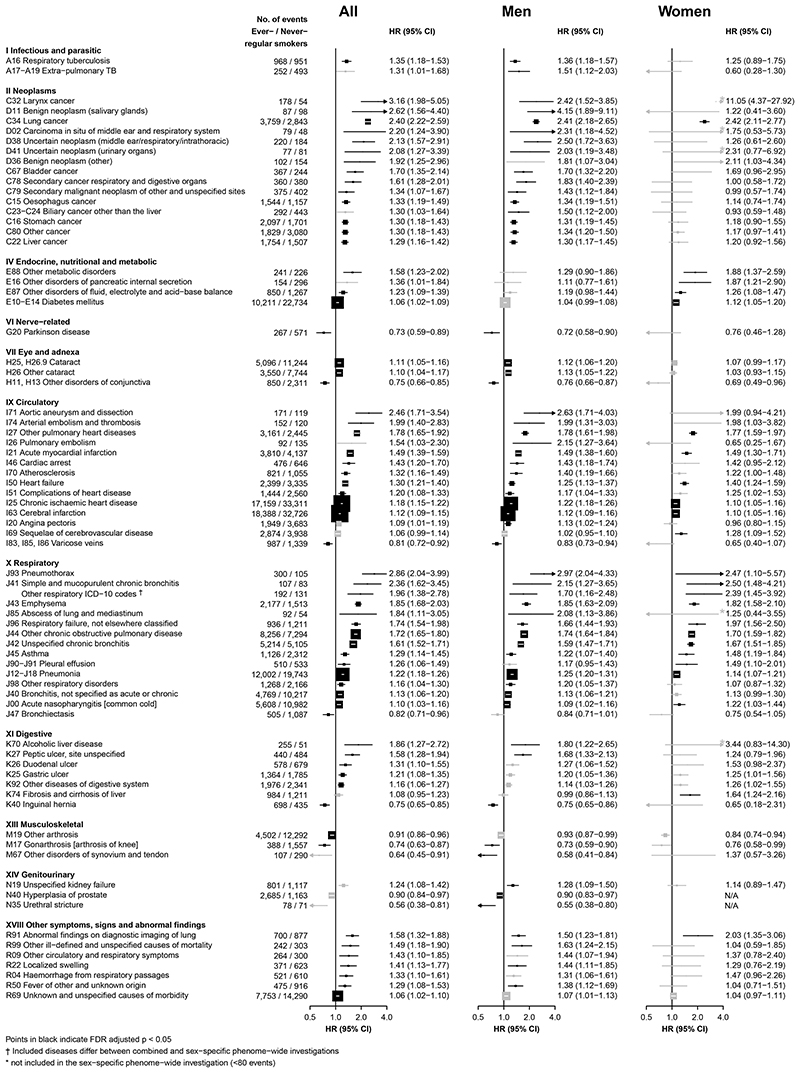
Adjusted HRs for cause-specific disease incidence significantly associated with ever-regular smoking Hazard ratios (HRs) were stratified by age-at-risk (5-year groups), sex, and study area and were adjusted for education and alcohol drinking. All analyse were restricted to age-at-risk range of 35-84 years. The solid boxes represent HRs, with the size inversely proportional to the variance of the logarithm of the HR, and the horizontal lines represent 95% confidence intervals. The individual diseases listed included all that showed FDR-adjusted significant associations with smoking, in overall or sex-specific analyses. The solid black and grey boxes indicate FDR-adjusted significant and non-significant associations, respectively.

Figure 1 shows the adjusted HRs for specific conditions showing FDR-significant positive associations with ever-smoking in sex-combined or sex-specific analyses. Overall in the combined analyses, the adjusted HRs ranged from 1·06 (1·02-1·09) for diabetes mellitus (ICD-10: E10-E14) to 3·16 (1·98-5·05) for larynx cancer (C32) (Figure 1). The HRs were generally higher in men than in women, with a few exceptions including larynx cancer (C32), lung cancer (C34) and several major respiratory diseases such as chronic bronchitis (J41), for which the HRs appeared similar or greater in women.

For the seven diseases showing an overall inverse association (i.e. Parkinson’s disease [G20], other disorders of conjunctiva [H11, H13], varicose veins [I83, I85, I86], bronchiectasis [J47], inguinal hernia [K40], other arthrosis [M19], gonarthrosis [M17]), the HRs ranged from 0·82 (0·71-0·96) for bronchiectasis to 0.73 (0.59-0.89) for Parkinson’s disease. Among women, there was no apparent association of ever-smoking with cancer of the breast (n=74/2,646 cases in ever-/never-smokers; 0·94 [0·74-1·20]) or corpus uteri (mostly endometrium; 15/562; 0·97 [0·56-1·68]). The HRs for all disease-specific morbidity under each ICD-10 chapter examined are shown in eFigures 3-20. These associations were unaltered in sensitivity analyses with additional adjustment.

Of the 85 mortality endpoints examined, ever-smoking was significantly associated with higher risks of 28 causes of death, with 22 associations remaining significant after FDR adjustment (men 17, women 9), including eight circulatory diseases, five respiratory diseases and seven cancers (Table 1 and eFigure 21). Of these 22 mortality endpoints, 16 showed significant, albeit generally more modest, positive associations with smoking in the morbidity analyses (eFigure 21, Figure 1, eTable 6). For the six other endpoints, all morbidity analyses showed directionally consistent, though non-significant after FDR-adjustment, positive associations. For major causes of death, there were generally similar HRs between male and female smokers (eFigure 21). After FDR adjustment no single cause of death showed lower risk among smokers. Additional adjustments for potential confounders did not alter the HRs for all the diseases showing either positive or inverse associations with smoking (eFigure 22).

For the five leading diseases, male smokers who had started smoking at younger ages or smoked more cigarettes (or equivalents) per day had consistently higher HRs for both morbidity and mortality in a dose-response manner (eTable 7) and also separately in urban and rural men (Table 2). Moreover, the HRs were typically higher in urban than in rural men (Table 2) and for mortality than morbidity (eTable 8). Similar, albeit less extreme, findings were also evidence in women (eTable 7). Sensitivity analyses with exclusion of individuals with a prior history of specific diseases yielded similar results (eTables 9 and 10).

For overall mortality, the adjusted HRs were 1·33 (1·29-1·37) in male ever-smokers, higher in urban than in rural areas (1·50 [1·42-1·58] versus 1·25 [1·20-1·30]) (eTables 2, 3, 11). Among women it was 1·44 (1·36-1·52) (eTable 12), again higher in urban than rural areas (1·53 [1·41-1·66] versus 1·38 [1·29-1·48]). These HRs were not materially altered after exclusion of participants with prior cancer at baseline (eTables 2, 3, 11, 12).

**Figure 2:**
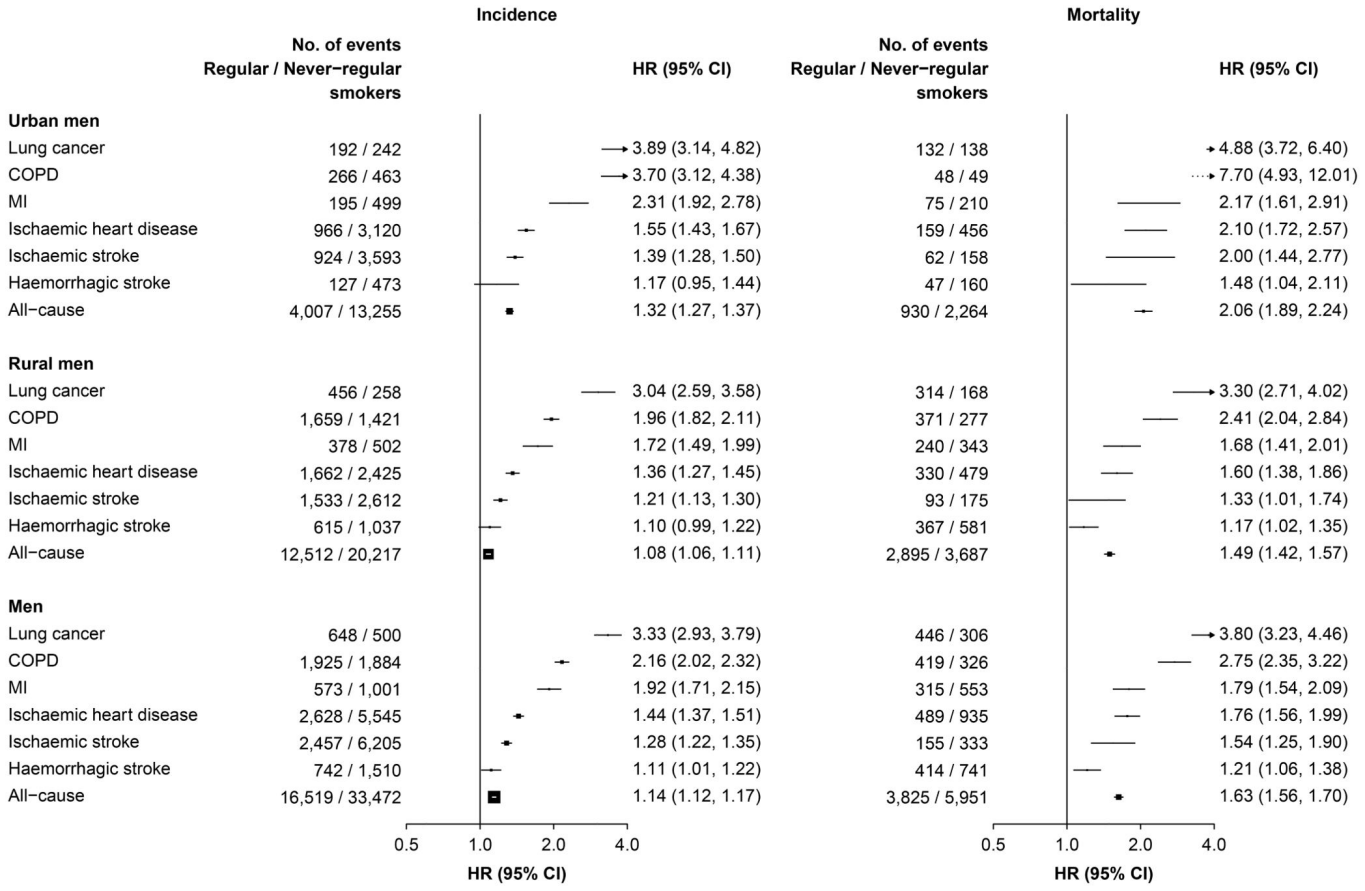
Adjusted HRs for risks of selected major disease incidence and mortality in urban and rural men who started smoking before 20 years old Convention as in Figure 1.

Moreover, regular-smokers who started smoking regularly at younger age had higher risks for major disease morbidity and mortality, particularly in urban areas (Figure 2). Among urban men who began smoking at <18 years, the HRs were 2·06 (1·89-2·24) for overall mortality and 1·32 (1·27-1·37) for any disease incidence (Figure 2), increasing to 2·35 (2·07-2·67) and 1·40 (1·30-1·50), respectively, among those who began at <15 years.

Compared to never-smokers, ever-smokers in either sex had worse survival starting at age of 55 years, with smokers reaching 50% survival ~3.5 years earlier (eFigure 23). After accounting for excess mortality (i.e. competing risk), ever- smokers had significantly higher total expected hospitalisations and (to a lesser extent) longer stay in hospital compared with never-smokers (eFigure 24), especially in men and for those attributed to cancer and respiratory disease (eFigure 25). The differences broadly increased along with age-at-risk, with apparent divergence beginning at around 55 to 60 years of age.

Among male ex-smokers who had quitted due to illness, there were significant excess risks of overall mortality (1·61, 1·55-1·66) and morbidity (1·25, 1·22-1·27) (eTable 11), which, although decreasing gradually, persisted beyond 15 years after quitting at baseline (eFigure 26). In contrast, those who had stopped voluntarily (i.e. before developing major diseases) only had small excess risks for overall mortality (1·06, 1·01-1·11) and morbidity (1·05, 1·03-1·08), with the risks approaching those among never smokers after about 5-10 years of quitting. Similarly contrasting differences between the two groups of ex-smokers were also observed for the top-five disease incidence and mortality (eTables 11-12).

**Figure 3:**
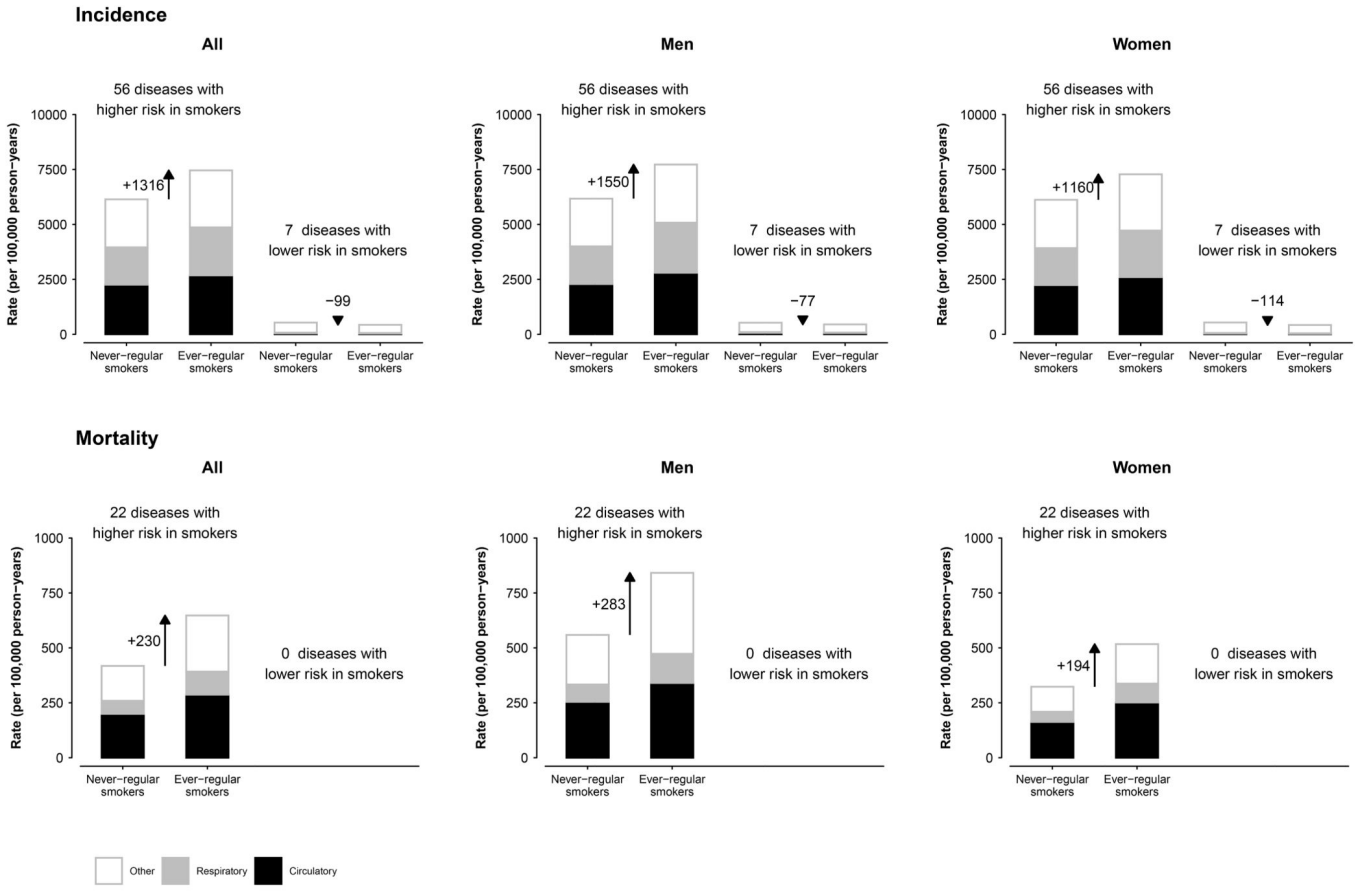
Incidence and mortality rates from all diseases found to have a FDR-adjusted significant association with ever-regular smoking The bar diagrams indicate the overall absolute morbidity and mortality rates per 100,000 person-years at age-at-risk 35-84 years in never- versus ever-regular smokers, overall and in men and women separately. Within each bar diagram, separate rates were also shown for circulatory (black), respiratory (grey) and other diseases (white). The morbidity analyses included 56 diseases showing positive associations with smoking and seven showing inverse associations. The mortality analyses were based on the 22 causes of death showing significant positive associations with smoking.

When considering all FDR-adjusted significant positive (n=56) and inverse (n=7) associations simultaneously, smoking was associated with an additional 13 (men 20; women 10) events for each one event prevented, corresponding to a net absolute excess of 1,217 (men 1,473; women 1,046) events per 100,000 person-years (Figure 3). For mortality, smoking was associated with a net excess of 230 (men 283; women 194) deaths per 100,000 person-years. Overall in this population, 19·6% of deaths in men (24·3% urban, 16·2% rural) and 2·8% of deaths in women at ages 35-84 years could be attributed to smoking if all the FDR-adjusted significant associations were causal.

## Discussion

This study provided the first comprehensive assessment of the long-term health effects of tobacco smoking on a wide range of diseases in adult men and women in China. Overall, smoking was significantly associated with higher risks of 22 causes of death and 56 individual diseases across all major organ systems, as well as more episodes and longer durations of hospitalisation. The associations were stronger in urban than in rural areas, and in those who started at younger age and smoked larger amount of tobacco. Although the relative risks associated with smoking were still relatively modest for most diseases, among urban men who started smoking before 18 years of age, the HR for overall mortality already approached those observed in high-income Western populations where most smokers started during young adulthood. Furthermore, we also showed that stopping smoking before the onset of major illness is remarkably beneficial.

Decades of epidemiological studies in many Western countries such as the UK and USA have demonstrated substantial hazards of tobacco smoking.^5,34^ With a few exceptions^35–37^, however, most previous studies were unable to investigate simultaneously the associations with a broad range of diseases. By synthesising evidence from various sources, the 2014 US Surgeon General Report concluded that 26 diseases were likely causally associated with smoking.^5^ More recently, the Global Burden of Disease Study (GBD) concluded 39 diseases (and nine causes of injuries) were likely associated with smoking (see eTable 13).^34^ For many other diseases (e.g. ICH, heart failure, influenza, or gastrointestinal conditions), the existing evidence is less conclusive, coming mainly from case-control or small cohort studies.^9,38,39^ In China and other LMICs, most existing prospective evidence was mainly on mortality from several major diseases.^6,11–13,40^ In this large prospective study, we found smoking to be associated with increased mortality from 22 causes and increased morbidity from 56 conditions, including many that are still largely under-studied in LMICs (e.g. ICH, heart failure, aortic aneurysm, pulmonary embolism, peptic ulcer).^5^ Most of these associations overlap with those reported consistently in previous cohort studies of Western populations.

Long-term prospective studies in Western countries have also demonstrated clearly the long delay between widespread uptake of cigarette smoking in young adult population, first in men then women, and rising morbidity and mortality risks subsequently.^41^ In these Western populations where the smoking epidemic has matured, most adult smokers started at a young age, smoked a large amount, and persistently consumed manufactured cigarette (as opposed to traditional tobacco products) for decades, which are the hallmark of “high risk smoking patterns”. Therefore, contemporary studies in those populations could reliably capture the “full effects” of prolonged cigarette smoking.^35,42^ In contrast, as the widespread uptake and peak of cigarette smoking in China was much more recent, adult smokers in CKB who were born mainly before 1970s had a later starting age, consumed fewer cigarettes daily, and used more traditional tobacco products (which are less harmful than cigarettes) compared to Western smokers^6,11^ or younger smokers (i.e. those born after 1970s) in China. These differences in smoking patterns likely explain the generally weaker associations in CKB than those reported in Western studies.^35,36^ Our findings are, however, highly consistent with previous studies in China^6,11,12^ and other LMICs where the widespread use of cigarette is relatively recent.^11,12,14^

Similarly, the greater excess risks for most diseases in urban than rural men smokers likely reflect more advanced smoking epidemic in urban than rural China, as cigarettes had been less available and affordable in rural areas until recent decades.^43^ These may also partly explain the lack of apparent associations in the present study for certain conditions known to be linked, albeit modestly, to smoking (e.g. prostate cancer, colorectal cancer, dementia). Likewise, the elevated background disease risk for many diseases, especially in rural areas, due to greater exposure to traditional risk factors (e.g. household air pollution, chronic infection) may also play a role. For certain diseases (e.g. ICH) that are much less common in Western populations, previous studies have reported mixed findings.^38^ With a much larger number of well-characterised ICH cases (>10,000; >80% confirmed by imaging), we found significantly elevated risks of ICH mortality and incidence, particularly among urban men.

Among women, despite the low smoking prevalence (<3%) and intensity among smokers, we found significant excess risks of 20 distinct conditions. With a few exceptions (e.g. lung cancer, COPD), the HRs for most disease-specific morbidities, but not necessarily mortalities, were more modest in female than male smokers. The lack of apparent sex difference in HRs for lung cancer and COPD, corroborates to previous findings,^36,44^ suggesting that women’s respiratory system appears more vulnerable to the harm of smoking. This may partly reflect the potential protective role of oestrogen in these diseases and the anti-oestrogenic effects of tobacco smoke^45^ as well as genetic predisposition of early onset COPD and lung cancer in women.^44^ For breast cancer we did not find any significant associations, consistent with previous Chinese studies,^46^ but not with those in Western populations, which generally showed positive association.^5,35^

However, the recorded case numbers among smokers in this and other Chinese studies were very small.

Previous Western studies have also reported lower risks of certain diseases among smokers, particularly Parkinson’s disease and endometrium cancer.^5^ For Parkinson’s disease, we have also found a significant 27% lower risk among smokers. For the other conditions, we found no clear associations with smoking, but the case numbers were small (e.g. only 15 corpus uteri cancer cases in female smokers). For six other conditions, however, we found lower risks among smokers, but there was very limited previous evidence. Among these, varicose veins, inguinal hernia, gonarthrosis, and other arthrosis are all strongly linked to family history that cannot be controlled for in CKB;^47–51^ and all except inguinal hernia have been associated with higher BMI,^47–51^ while smoking is known to have a weight reduction effect. Further adjustment for BMI, occupation and self-rated health, however, did not change the associations materially, suggesting no strong residual confounding by these factors. Bronchiectasis is associated with severe lung infections, asthma and cystic fibrosis but not known to be linked to smoking.^47^ Reverse causation bias can arise from the inclusion of diseased individuals who were less likely to start or more likely to quit early in life. Similar bias may also apply to conjunctiva disorders, which tend to develop during early adulthood with recurrent irritating symptoms or even vision impairment. Sensitivity analyses to exclude the first five years of follow-up, however, did not alter the results materially (eTable 14). Nonetheless, the inverse associations could also represent chance findings, given the large number of tests conducted. With the lack of prior evidence, our findings could only be considered as hypothesis-generating, which require further verifications in other studies.

Our study has several strengths, including large sample size, reliable assessment of smoking exposure, completeness of follow-up, and the broad range of diseases included. However, it also has limitations. First, for rare conditions (e.g. leukaemia, peripheral artery disease) the case numbers remain small, especially for women. Second, we only assessed prior medical history of ~20 major diseases, so we were unable to account fully for reverse causality for many other outcomes. Third, the use of FDR adjustment may have obscured any true but modest associations for certain conditions (e.g. hyperthyroidism) that have been associated with smoking. Fourth, although the baseline smoking habits had been objectively validated,^23^ subsequent changes in smoking patterns (e.g. quitting) could yield underestimated HRs. In the 2013-14 resurvey of a random subset of ~25,000 surviving participants, we found that about one-fifth of baseline smokers stopped subsequently. Despite this, we could not directly assess the likely resulting underestimation of risks associated with changes in smoking patterns. Fifth, the record linkage may miss milder (e.g. influenza) or underdiagnosed conditions (e.g. COPD, dementia), resulting in underestimation of the disease burden associated with smoking. Sixth, CKB was not nationally representative, and the study participation was voluntary, so healthy volunteer bias is inevitable. However, the large sample size, diverse areas covered, heterogeneity of exposure, and highly consistent smoking patterns with those reported in other representative surveys in China^6,18^ means that our HRs for smoking could still be largely generalisable to the Chinese population.^52,53^ Finally, unlike for mortality, we were not able to estimate the total disease morbidity burden attributed to smoking in China, due to the lack of nationally representative disease incidence data and regional variations in accessing health service.

In recent decades various estimates have been made about the smoking-attributed mortality burden in China. We showed that smoking now accounted for 20% and 3% of male and female deaths respectively at age 35-84 years. This would translate into >1 million total deaths per year in China, in line with our previous projection,^6^ but much lower than that estimated in the 2019 GBD study (2·1 million men and 0·3 million women).^34^ The GBD estimates would suggest that about half of all adult male deaths in China (~4·5 million in 2019) could be attributed to smoking. The GBD report used relative risk estimates for 36 causes of death (as opposed to all-cause mortality in this study) derived from pooled analyses of mainly Western cohort and case-control studies.^34^ Although various adjustments were made,^34^ it did not appear to fully account for the delayed effects and large urban-rural differences of recent cigarette uptake in China. Nevertheless, our study provided reliable evidence that if the current trends in smoking persist, the future smoking attributed disease burden in Chinese men is likely to increase markedly, whereas that in women it will likely remain low and continue to decline.^6^

In summary, the present study demonstrated substantial hazards of smoking from a wide range of conditions among Chinese men and women. Given the delayed effects, the future risk per individual smoker and overall disease burden attributed to smoking among adult Chinese men will be much greater, especially in those who were born in the 1970s-1980s, who have reached adulthood when nationwide cigarette consumption was high. As demonstrated in the present and many previous studies,^6,35^ stopping smoking before the onset of major illness is remarkably beneficial, and widespread smoking cessation, facilitated through increased tobacco tax, effective package warnings, and cessation clinics and helplines,^54^ offer China one of the most effective strategies to control the rising burden of chronic diseases over the next few decades.

## Supplementary Material

etables, efigures

## Figures and Tables

**Table 1:** Number of morbidity and mortality events by ICD-10 chapter and their overall associations with ever-regular smoking, for men and women combined

						No. of significant associations							
						Without FDR adjustment				With FDR adjustment			
		No. of diseases		No. of cases		Positive		Inverse		Positive		Inverse	
ICD-10 Chapter		Incidence	Death	Incidence	Death	Incidence	Death	Incidence	Death	Incidence	Death	Incidence	Death
I	Infectious and parasitic	21	4	28,572	624	3	1	0	0	1	0	0	0
II	Neoplasms	72	22	59,716	15,811	18	9	0	0	14	7	0	0
III	Blood and immune-related	7	1	6,146	90	0	0	1	0	0	0	0	0
IV	Endocrine, nutritional and metabolic	13	2	47,809	1,397	5	0	0	0	3	0	0	0
V	Mental and behavioural	12	4	8,109	140	0	0	2	0	0	0	0	0
VI	Nerve-related	21	3	23,065	375	1	1	1	1	0	0	1	0
VII	Eye and adnexa	24	-	43,182	-	2	-	1	-	2	-	1	-
VIII	Ear and mastoid process	5	-	7,317	-	0	-	0	-	0	-	0	-
IX	Circulatory	41	19	279,306	19,836	14	9	1	0	10	8	1	0
X	Respiratory	21	10	175,637	4,564	14	5	1	0	14	5	1	0
XI	Digestive	51	3	132,817	1,038	10	1	4	0	5	1	1	0
XII	Skin and subcutaneous tissue	11	1	12,273	29	2	0	1	0	0	0	0	0
XIII	Musculoskeletal	34	1	99,111	125	1	0	5	0	0	0	2	0
XIV	Genitourinary	54	2	78,577	552	2	0	5	0	0	0	0	0
XV	Pregnancy-related	6	1	3,745	1	1	0	0	0	0	0	0	0
XVIII	Other symptoms, signs and abnormal findings	35	3	87,254	831	10	2	0	0	7	1	0	0
XIX	Injury, poisoning and other external causes	38	1	41,073	79	4	0	0	0	0	0	0	0
XX	External causes	10	8	3,894	3,268	0	0	1	1	0	0	0	0
	**Total**	**476**	**85**	**1,137,603**	**48,760**	**87**	**28**	**23**	**2**	**56**	**22**	**7**	**0**

**Table 2:** Adjusted HRs for incident risks of five major diseases associated with smoking, among urban and rural men

Smoking category	Lung cancer		Ischaemic heart disease		Ischaemic stroke		Haemorrhagic stroke		COPD	
	Number of events	HR (95% CI)^†^	Number of events	HR (95% CI)^†^	Number of events	HR (95% CI)^†^	Number of events	HR (95% CI)^†^	Number of events	HR (95% CI)^†^
**Urban men**										
Never-regular smoker	242	1.00 (0.88, 1.14)	3,120	1.00 (0.96, 1.04)	3,593	1.00 (0.97, 1.03)	473	1.00 (0.91, 1.10)	463	1.00 (0.91, 1.10)
Ever-regular smoker	1,227	2.58 (2.24, 2.98)	7,220	1.27 (1.22, 1.33)	7,286	1.14 (1.09, 1.19)	996	1.14 (1.02, 1.28)	1,576	2.26 (2.02, 2.51)
Ex-smoker (by choice)^‡^	134	1.48 (1.25, 1.76)	1,139	1.10 (1.03, 1.16)	1,201	1.01 (0.96, 1.07)	152	1.01 (0.86, 1.19)	205	1.40 (1.22, 1.61)
Regular smoker	1,093	2.87 (2.70, 3.06)	6,081	1.31 (1.28, 1.35)	6,085	1.17 (1.14, 1.20)	844	1.17 (1.09, 1.26)	1,371	2.50 (2.37, 2.64)
Ex-smoker (ill health)	190	2.35 (2.03, 2.71)	1,288	1.55 (1.46, 1.63)	1,162	1.16 (1.10, 1.23)	180	1.37 (1.18, 1.58)	415	3.13 (2.84, 3.45)
Current smoker	903	3.04 (2.83, 3.26)	4,793	1.26 (1.22, 1.30)	4,923	1.17 (1.13, 1.20)	664	1.12 (1.03, 1.22)	956	2.28 (2.13, 2.44)
Age began smoking (years)^*^										
25+	306	2.23 (1.99, 2.49)	1,954	1.23 (1.18, 1.29)	2,020	1.09 (1.04, 1.14)	278	1.13 (1.01, 1.27)	436	1.95 (1.77, 2.14)
18-24	595	3.14 (2.89, 3.41)	3,161	1.33 (1.28, 1.38)	3,141	1.18 (1.14, 1.23)	439	1.19 (1.08, 1.31)	669	2.67 (2.47, 2.88)
<18	192	3.64 (3.15, 4.21)	966	1.55 (1.45, 1.65)	924	1.33 (1.25, 1.42)	127	1.19 (0.99, 1.42)	266	3.73 (3.30, 4.22)
No. smoked (cig/day)^*^										
<15	335	2.17 (1.94, 2.42)	2,280	1.20 (1.15, 1.25)	2,337	1.09 (1.05, 1.14)	316	1.12 (1.00, 1.25)	478	2.14 (1.95, 2.35)
15-24	525	3.06 (2.81, 3.34)	2,722	1.33 (1.28, 1.38)	2,747	1.19 (1.15, 1.24)	387	1.18 (1.07, 1.30)	630	2.56 (2.37, 2.77)
25+	233	4.03 (3.54, 4.59)	1,079	1.65 (1.56, 1.76)	1,001	1.29 (1.21, 1.37)	141	1.26 (1.06, 1.49)	263	3.11 (2.75, 3.51)
**Rural men**										
Never-regular smoker	258	1.00 (0.88, 1.13)	2,425	1.00 (0.96, 1.04)	2,612	1.00 (0.96, 1.04)	1,037	1.00 (0.94, 1.06)	1,421	1.00 (0.95, 1.05)
Ever-regular smoker	2,215	2.23 (1.96, 2.55)	9,668	1.19 (1.14, 1.25)	9,429	1.11 (1.06, 1.16)	3,649	0.99 (0.92, 1.06)	7,799	1.52 (1.44, 1.61)
Ex-smoker (by choice)^‡^	137	1.56 (1.32, 1.84)	723	1.11 (1.03, 1.20)	738	0.99 (0.92, 1.07)	271	0.95 (0.85, 1.08)	470	1.14 (1.04, 1.25)
Regular smoker	2,078	2.30 (2.20, 2.41)	8,945	1.20 (1.18, 1.23)	8,691	1.12 (1.09, 1.14)	3,378	0.99 (0.96, 1.03)	7,329	1.56 (1.52, 1.59)
Ex-smoker (ill health)	219	1.97 (1.72, 2.25)	1,187	1.51 (1.42, 1.59)	1,160	1.31 (1.24, 1.39)	432	1.18 (1.08, 1.30)	1,218	2.25 (2.12, 2.38)
Current smoker	1,859	2.35 (2.24, 2.47)	7,758	1.16 (1.14, 1.19)	7,531	1.09 (1.07, 1.12)	2,946	0.97 (0.93, 1.00)	6,111	1.46 (1.43, 1.50)
Age began smoking (years)^*^										
25+	526	1.71 (1.57, 1.86)	2,824	1.09 (1.05, 1.14)	2,802	1.06 (1.02, 1.10)	1,151	0.94 (0.89, 1.00)	1,926	1.22 (1.17, 1.28)
18-24	1,096	2.42 (2.28, 2.57)	4,459	1.22 (1.18, 1.26)	4,356	1.13 (1.09, 1.16)	1,612	0.99 (0.94, 1.04)	3,744	1.65 (1.60, 1.70)
<18	456	3.13 (2.85, 3.43)	1,662	1.37 (1.31, 1.44)	1,533	1.21 (1.15, 1.27)	615	1.09 (1.01, 1.18)	1,659	1.97 (1.87, 2.07)
No. smoked (cig/day)^*^										
<15	524	1.70 (1.55, 1.85)	3,459	1.16 (1.12, 1.20)	3,531	1.08 (1.05, 1.12)	1,420	1.04 (0.99, 1.10)	2,234	1.43 (1.37, 1.49)
15-24	994	2.40 (2.26, 2.56)	3,798	1.20 (1.16, 1.24)	3,614	1.12 (1.09, 1.16)	1,342	0.92 (0.88, 0.98)	3,313	1.61 (1.55, 1.67)
25+	560	3.27 (3.00, 3.57)	1,688	1.31 (1.25, 1.38)	1,546	1.21 (1.15, 1.28)	616	1.01 (0.93, 1.10)	1,782	1.68 (1.60, 1.76)

† All analyses were stratified by study area and 5-year age-at-risk group and adjusted for education and alcohol drinking.

‡ Only include those who had quit voluntarily (i.e. not due to ill health).

* Include current smokers and ex-smokers who had quit due to ill health.

## Data Availability

The China Kadoorie Biobank (CKB) is a global resource for the investigation of lifestyle, environmental, blood biochemical and genetic factors as determinants of common diseases. The CKB study group is committed to making the cohort data available to the scientific community in China, the UK and worldwide to advance knowledge about the causes, prevention and treatment of disease. For detailed information on what data is currently available to open access users and how to apply for it, visit: http://www.ckbiobank.org/site/Data+Access. Researchers who are interested in obtaining the raw data from the China Kadoorie Biobank study that underlines this paper should contact ckbaccess@ndph.ox.ac.uk. A research proposal will be requested to ensure that any analysis is performed by bona fide researchers and - where data is not currently available to open access researchers - is restricted to the topic covered in this paper.

## Research in context

### Evidence before this study

We searched the PubMed database for relevant prospective cohort studies in China published in English before 1 Jan 2022, using the terms “smoking OR tobacco” AND “China” AND “mortality OR death OR incidence OR risk”. The majority of the previous studies were conducted before 2000 and focused only on mortality from several major diseases such as cancer, cardiovascular disease, and chronic respiratory diseases. A few large prospective cohort studies (each with >100,000 participants) in China, including our earlier reports based on the China Kadoorie Biobank (2004-2008) also demonstrated significant associations of smoking with incidence of several major diseases including site-specific cancer, and diabetes. Additionally, a recent pooled analysis of six prospective cohorts with >330,000 primarily Chinese adults found significantly higher risk of nasopharyngeal cancer associated with smoking. However, there is no report in China assessing systematically the associations of smoking with a broader range of fatal and non-fatal diseases. Outside of China, only a few large prospective studies, such as the UK Million Women Study (1·3 million women) and pooled data from several US cohort studies (involving 0·5-1 million adults), have examined the associations of smoking with risks of mortality, but not incidence, from a wide range of disease outcomes simultaneously. Overall, these large UK and US studies reported significant associations of smoking with 23 and 35 causes of deaths, respectively.

### Added value of this study

To our knowledge, this is the first large nationwide prospective cohort study in China to systematically examine the associations of smoking with diseases affecting all body systems. After accounting for multiple-testing, smoking was significantly associated with higher disease incidence from 56 (man 50, women 24) specific diseases (10 cardiovascular, 14 respiratory, 14 cancer, five digestive, 13 others), and with higher risks of death from 22 (men 17, women 9) specific causes. This study provides important new evidence on the health effects of smoking beyond mortality from major diseases in China. It also provides additional evidence demonstrating greater relative risk of morbidity and mortality among urban male smokers, especially among those who started young. Furthermore, for the first time, we estimated the excess total and cause-specific (i.e. cardiovascular, respiratory and cancer) hospitalisation episodes and duration of hospitalisation associated with smoking in China. Apart from hazards of smoking, this study also demonstrated that stopping smoking before the onset of major illness is remarkably beneficial.

### Implications of all the available evidence

Decades of epidemiological research have produced strong evidence on the adverse health effects of smoking on an extensive range of major diseases. In China and many other low- and middle-income countries, the future disease morbidity and mortality burden attributed to smoking will likely increase. Large prospective cohort studies with reliable electronic linkage not only to mortality but also to hospital records in diverse populations could help to assess and monitor the evolution of the tobacco epidemic by providing population-specific estimates on the current and future disease burden attributed to smoking. Such evidence is crucial to inform appropriate policy action in tobacco control nationally and globally.

## References

[1] Eriksen M, Mackay J, Schluger N, Gomeshtapeh FI, Drope J (2015). The Tobacco Atlast.

[2] Chinese Center for Disease Control and Prevention China Adult Tobacco Survey Report 2018: Chinese Center for Disease Control and Prevention, 2019.

[3] Doll R, Peto R (1981). The causes of cancer: quantitative estimates of avoidable risks of cancer in the United States today. J Natl Cancer Inst.

[4] Wald N, Nicolaides-Bouman A (1991). UK Smoking Statistics.

[5] US Department of Health and Human Services (2014). The health consequences of smoking - 50 years of progress: a report of the Surgeon General.

[6] Chen Z, Peto R, Zhou M (2015). Contrasting male and female trends in tobacco-attributed mortality in China: evidence from successive nationwide prospective cohort studies. Lancet.

[7] Scott JG, Matuschka L, Niemela S, Miettunen J, Emmerson B, Mustonen A (2018). Evidence of a Causal Relationship Between Smoking Tobacco and Schizophrenia Spectrum Disorders. Front Psychiatry.

[8] Xia J, Wang L, Ma Z (2017). Cigarette smoking and chronic kidney disease in the general population: a systematic review and meta-analysis of prospective cohort studies. Nephrol Dial Transplant.

[9] Lawrence H, Hunter A, Murray R, Lim WS, McKeever T (2019). Cigarette smoking and the occurrence of influenza - Systematic review. J Infect.

[10] Barbhaiya M, Tedeschi SK, Lu B (2018). Cigarette smoking and the risk of systemic lupus erythematosus, overall and by anti-double stranded DNA antibody subtype, in the Nurses’ Health Study cohorts. Ann Rheum Dis.

[11] Yang JJ, Yu D, Wen W (2019). Tobacco Smoking and Mortality in Asia: A Pooled Meta-analysis. JAMA Netw Open.

[12] Yang JJ, Yu D, Shu XO (2021). Quantifying the association of low-intensity and late initiation of tobacco smoking with total and cause-specific mortality in Asia. Tob Control.

[13] Thomson B, Rojas NA, Lacey B (2020). Association of childhood smoking and adult mortality: prospective study of 120 000 Cuban adults. Lancet Glob Health.

[14] Sathish T, Teo KK, Britz-McKibbin P (2022). Variations in risks from smoking between high-income, middle-income, and low-income countries: an analysis of data from 179 000 participants from 63 countries. The Lancet Global Health.

[15] Wang B, Xiao D, Wang C (2015). Smoking and chronic obstructive pulmonary disease in Chinese population: a meta-analysis. Clin Respir J.

[16] Yu K, Zhu S, He M (2021). Epidemiological characteristics of 561 cases of intracerebral hemorrhage in Chengdu, China. Medicine (Baltimore).

[17] Zhang R, Li H, Li N (2021). Risk factors for gastric cancer: a large-scale, population-based case-control study. Chin Med J (Engl).

[18] Wang M, Luo X, Xu S (2019). Trends in smoking prevalence and implication for chronic diseases in China: serial national cross-sectional surveys from 2003 to 2013. Lancet Resp Med.

[19] Pan A, Wang Y, Talaei M, Hu FB, Wu T (2015). Relation of active, passive, and quitting smoking with incident type 2 diabetes: a systematic review and meta-analysis. Lancet Diabetes Endocrinol.

[20] Chen Z, Lee L, Chen J (2005). Cohort profile: the Kadoorie Study of Chronic Disease in China (KSCDC). Int J Epidemiol.

[21] Chen Z, Chen J, Collins R (2011). China Kadoorie Biobank of 0.5 million people: survey methods, baseline characteristics and long-term follow-up. Int J Epidemiol.

[22] Yang GH, Stroup DF, Thacker SB (1997). National public health surveillance in China: implications for public health in China and the United States. Biomed Environ Sci.

[23] Zhang Q, Li L, Smith M (2013). Exhaled carbon monoxide and its associations with smoking, indoor household air pollution and chronic respiratory diseases among 512,000 Chinese adults. Int J Epidemiol.

[24] Murray CJL, Aravkin AY, Zheng P (2020). Global burden of 87 risk factors in 204 countries and territories, 1990-2019: a systematic analysis for the Global Burden of Disease Study 2019. Lancet.

[25] Lau B, Cole SR, Gange SJ (2009). Competing Risk Regression Models for Epidemiologic Data. Am J Epidemiol.

[26] Plummer M (2004). Improved estimates of floating absolute risk. Stat Med.

[27] Grambsch PM, Therneau TM (1994). Proportional hazards tests and diagnostics based on weighted residuals. Biometrika.

[28] Dong H, Robison LL, Leisenring WM, Martin LJ, Armstrong GT, Yasui Y (2015). Estimating the Burden of Recurrent Events in the Presence of Competing Risks: The Method of Mean Cumulative Count. Am J Epidemiol.

[29] Ghosh D, Lin DY (2000). Nonparametric analysis of recurrent events and death. Biometrics.

[30] Cook RJ, Lawless JF (1997). Marginal analysis of recurrent events and a terminating event. Stat Med.

[31] Benjamini Y, Hochberg Y (1995). Controlling the False Discovery Rate: A Practical and Powerful Approach to Multiple Testing. J R Stat Soc Series B Stat Methodol.

[32] Braun JM, Kalloo G, Kingsley SL, Li N (2019). Using phenome-wide association studies to examine the effect of environmental exposures on human health. Environment International.

[33] Glickman ME, Rao SR, Schultz MR (2014). False discovery rate control is a recommended alternative to Bonferroni-type adjustments in health studies. J Clin Epidemiol.

[34] Reitsma MB, Kendrick PJ, Ababneh E (2021). Spatial, temporal, and demographic patterns in prevalence of smoking tobacco use and attributable disease burden in 204 countries and territories, 1990–2019: a systematic analysis from the Global Burden of Disease Study 2019. Lancet.

[35] Pirie K, Peto R, Reeves GK, Green J, Beral V (2013). The 21st century hazards of smoking and benefits of stopping: a prospective study of one million women in the UK. Lancet.

[36] Carter BD, Abnet CC, Feskanich D (2015). Smoking and mortality--beyond established causes. N Engl J Med.

[37] Lariscy JT, Hummer RA, Rogers RG (2018). Cigarette Smoking and All-Cause and Cause-Specific Adult Mortality in the United States. Demography.

[38] Cho S, Rehni AK, Dave KR (2021). Tobacco Use: A Major Risk Factor of Intracerebral Hemorrhage. J Stroke.

[39] Lee H, Son YJ (2019). Influence of Smoking Status on Risk of Incident Heart Failure: A Systematic Review and Meta-Analysis of Prospective Cohort Studies. Int J Environ Res Public Health.

[40] Gajalakshmi V, Peto R, Kanimozhi VC, Whitlock G, Veeramani D (2007). Cohort profile: The Chennai Prospective Study of Mortality among 500 000 adults in Tamil Nadu, South India. Int J Epidemiol.

[41] Thun MJ, Carter BD, Feskanich D (2013). 50-year trends in smoking-related mortality in the United States. N Engl J Med.

[42] Di Cicco ME, Ragazzo V, Jacinto T (2016). Mortality in relation to smoking: the British Doctors Study. Breathe (Sheff).

[43] Huang X (2006). The China tobacco history.

[44] Gut-Gobert C, Cavailles A, Dixmier A (2019). Women and COPD: do we need more evidence?. Eur Respir Rev.

[45] Tankó LB, Christiansen C (2004). An update on the antiestrogenic effect of smoking: a literature review with implications for researchers and practitioners. Menopause (New York, NY).

[46] Chen C, Huang YB, Liu XO (2014). Active and passive smoking with breast cancer risk for Chinese females: a systematic review and meta-analysis. Chin J Cancer.

[47] Chalmers JD, Chang AB, Chotirmall SH, Dhar R, McShane PJ (2018). Bronchiectasis. Nat Rev Dis Primers.

[48] Fukaya E, Flores AM, Lindholm D (2018). Clinical and Genetic Determinants of Varicose Veins. Circulation.

[49] Sorensen LT, Friis E, Jorgensen T (2002). Smoking is a risk factor for recurrence of groin hernia. World J Surg.

[50] Hemberg A, Holmberg H, Norberg M, Nordin P (2017). Tobacco use is not associated with groin hernia repair, a population-based study. Hernia.

[51] Veronese N, Maggi S, Michel J-P (2019). Prevention of Chronic Diseases and Age-Related Disability.

[52] Manolio TA, Collins R (2010). Enhancing the feasibility of large cohort studies. JAMA.

[53] Rothman KJ, Gallacher JE, Hatch EE (2013). Why representativeness should be avoided. Int J Epidemiol.

[54] US Department of Health and Human Services (2020). Smoking cessation: a report of the Surgeon General.

