# Supplementary material for: Tobacco smoking and risks of >470 diseases in China: a prospective cohort study
Source: Lancet Public Health. Author manuscript; Available in PMC 2022 Dec 14. (PMC7613927; doi:10.1016/S2468-2667(22)00227-4)
Supplement: etables, efigures [file EMS154406-supplement-etables__efigures.docx]

**Supplementary Appendix**

**Tobacco smoking and risks of >470 diseases in China: a prospective cohort study**

Ka Hung Chan^1,2^*, DPhil; Neil Wright^1^*, PhD; Prof Dan Xiao^3,4^, MD; Yu Guo^5^, MSc; Yiping Chen^1,6^, DPhil; Huaidong Du^1,6^, PhD; Ling Yang^1,6^, PhD; Iona Millwood^1,6^, DPhil; Pei Pei^7^, BSc; Junzheng Wang^8^, BSc; Iain Turnbull^1^, MRCP; Simon Gilbert ^1,6^, MSc; Daniel Avery^1^, MSc; Christiana Kartsonaki^1,6^, DPhil; Canqin Yu^7,9^, PhD; Prof Junshi Chen^10^, MD; Prof Jun Lv^7,9^, PhD; Prof Robert Clarke^1,6^, MD; Prof Rory Collins^1^, FRS; Prof Richard Peto^1^, FRS; Prof Liming Li^7,9^*^†^*, MPH; Prof Chen Wang^3,4^*^†^*, MD; Prof Zhengming Chen^1,6^*^†^*, DPhil

^1^ Clinical Trial Service Unit and Epidemiological Studies Unit, Nuffield Department of Population Health, University of Oxford

^2^ Oxford British Heart Foundation Centre of Research Excellence, University of Oxford

^3^ WHO Collaborating Center for Tobacco Cessation and Respiratory Diseases Prevention, China-Japan Friendship Hospital, Beijing, China

^4^ Institute of Respiratory Medicine, Chinese Academy of Medical Sciences, Beijing, China

^5^ National Center for Cardiovascular Disease Fuwai Hospital, Chinese Academy of Medical Science, Beijing, China

^6^ MRC Population Health Research Unit, Nuffield Department of Population Health, University of Oxford

^7^ Peking University Center for Public Health and Epidemic Preparedness & Response

^8^ Licang Center of Disease Control and Prevention, Qingdao, China

^9^ Department of Epidemiology and Biostatistics, School of Public Health, Peking University, Beijing, China

^10^ China National Center for Food Safety Risk Assessment, Beijing, China

**Contributed equally; ^†^Joint corresponding authors*

**Address for correspondence**

Professor Liming Li, Department of Epidemiology and Biostatistics, School of Public Health, Peking University, Beijing, China; email: [lmleeph@vip.163.com](mailto:lmleeph@vip.163.com);

Professor Chen Wang, Institute of Respiratory Medicine, Chinese Academy of Medical Sciences, Beijing 100730, China; Email: [wangchen@pumc.edu.cn](mailto:wangchen@pumc.edu.cn);

Professor Zhengming Chen, Nuffield Dept. of Population Health, University of Oxford, Old Road Campus, OX3 7LF, Oxford, UK; Email: [zhengming.chen@ndph.ox.ac.uk](mailto:zhengming.chen@ndph.ox.ac.uk).

Table of Content

[eTable 1: Selected baseline characteristics by smoking status in men and women 3](#_Toc106107985)

[eFigure 1: Adjusted HRs for ICD-10 chapter-specific incidence associated with ever-regular smoking 4](#_Toc106107986)

[eFigure 2: Adjusted HRs for ICD-10 chapter-specific mortality associated with ever-regular smoking 5](#_Toc106107987)

[eTable 2: Adjusted HRs for risks of any disease incidence and overall mortality associated with smoking with and without excluding those with prior cancer reported at baseline, in rural men 6](#_Toc106107988)

[eTable 3: Adjusted HRs for risks of any disease incidence and overall mortality associated with smoking with and without excluding those with prior cancer reported at baseline, in urban men 7](#_Toc106107989)

[eTable 4: Number of morbidity and mortality events by ICD-10 chapter and their overall associations with ever-regular smoking, in men 8](#_Toc106107990)

[eTable 5: Number of morbidity and mortality events by ICD-10 chapter and their overall associations with ever-regular smoking, in women 9](#_Toc106107991)

[eFigure 3: Adjusted HRs for incidence of specific types of infectious and parasitic diseases associated with ever-regular smoking in men and women combined 10](#_Toc106107992)

[eFigure 4: Adjusted HRs for incidence of specific types of neoplasms associated with ever-regular smoking in men and women combined 11](#_Toc106107993)

[eFigure 5: Adjusted HRs for incidence of specific types of blood and immune-related diseases associated with ever-regular smoking in men and women combined 12](#_Toc106107994)

[eFigure 6: Adjusted HRs for incidence of specific types of endocrine, nutritional and metabolic diseases associated with ever-regular smoking in men and women combined 13](#_Toc106107995)

[eFigure 7: Adjusted HRs for incidence of specific types of mental and behavioural disorders associated with ever-regular smoking in men and women combined 14](#_Toc106107996)

[eFigure 8: Adjusted HRs for incidence of specific types of nerve-related diseases associated with ever-regular smoking in men and women combined 15](#_Toc106107997)

[eFigure 9: Adjusted HRs for incidence of specific types of eye and adnexa diseases associated with ever-regular smoking in men and women combined 16](#_Toc106107998)

[eFigure 10: Adjusted HRs for incidence of specific types of ear and mastoid process diseases associated with ever-regular smoking in men and women combined 17](#_Toc106107999)

[eFigure 11: Adjusted HRs for incidence of specific types of circulatory diseases associated with ever-regular smoking in men and women combined 18](#_Toc106108000)

[eFigure 12: Adjusted HRs for incidence of specific types of respiratory diseases associated with ever-regular smoking in men and women combined 19](#_Toc106108001)

[eFigure 13: Adjusted HRs for incidence of specific types of digestive diseases associated with ever-regular smoking in men and women combined 20](#_Toc106108002)

[eFigure 14: Adjusted HRs for incidence of specific types of skin and subcutaneous tissue diseases associated with ever-regular smoking in men and women combined 21](#_Toc106108003)

[eFigure 15: Adjusted HRs for incidence of specific types of musculoskeletal diseases associated with ever-regular smoking in men and women combined 22](#_Toc106108004)

[eFigure 16: Adjusted HRs for incidence of specific types of genitourinary diseases associated with ever-regular smoking in men and women combined 23](#_Toc106108005)

[eFigure 17: Adjusted HRs for incidence of specific types of pregnancy-related diseases associated with ever-regular smoking in men and women combined 24](#_Toc106108006)

[eFigure 18: Adjusted HRs for incidence of other symptoms, signs and abnormal findings associated with ever-regular smoking in men and women combined 25](#_Toc106108007)

[eFigure 19: Adjusted HRs for incidence of specific types of injury, poisoning and other external causes associated with ever-regular smoking in men and women combined 26](#_Toc106108008)

[eFigure 20: Adjusted HRs for incidence of specific external causes associated with ever-regular smoking in men and women combined 27](#_Toc106108009)

[eFigure 21: Adjusted HRs for cause-specific mortality significantly associated with ever-regular smoking 28](#_Toc106108010)

[eTable 6: Comparison of FDR-adjusted significant positive associations with ever-regular smoking between morbidity and mortality analyses, overall and by sex 29](#_Toc106108011)

[eFigure 22: Adjusted HRs for cause-specific incidence significantly associated with ever-regular smoking after further adjustment 31](#_Toc106108012)

[eTable 7: Adjusted HRs for incident risks of five major diseases associated with smoking, among men and women 32](#_Toc106108013)

[eTable 8: Adjusted HRs for mortality risks of five major diseases associated with smoking, among urban and rural men 33](#_Toc106108014)

[eTable 9: Adjusted HRs for incident risks of five major diseases associated with smoking after exclusion of relevant prior disease reported at baseline, among men and women 34](#_Toc106108015)

[eTable 10: Adjusted HRs for incident risks of five major diseases associated with smoking after exclusion of relevant prior disease reported at baseline, among urban and rural men 35](#_Toc106108016)

[eTable 11: Adjusted HRs for risks of any disease incidence and overall mortality associated with smoking with and without excluding those with prior cancer reported at baseline, in men 36](#_Toc106108017)

[eTable 12: Adjusted HRs for risks of any disease incidence and overall mortality associated with smoking with and without excluding those with prior cancer reported at baseline, in women 37](#_Toc106108018)

[eFigure 23: Kaplan-Meier curves for overall survival from age-at-risk of 35, among men and women 38](#_Toc106108019)

[eFigure 24: Total expected hospitalisations and days in hospital from any causes from age-at-risk of 35 years among men and women 39](#_Toc106108020)

[eFigure 25: Total expected hospitalisations and days in hospital due to cancer, cardiovascular disease, respiratory disease and other conditions from age-at-risk of 35 years among men 40](#_Toc106108021)

[eFigure 26: Adjusted HRs for all-cause mortality and all disease incidence by years stopped smoking and reason stopped, among men 41](#_Toc106108022)

[eTable 13: Comparison of clinical conditions considered as likely causally related to smoking in the 2014 US Surgeon General Report and 2019 Global Burden of Disease Study† 42](#_Toc106108023)

[eTable 14: Adjusted HRs for diseases significantly (after FDR adjustment) inversely associated with smoking, after further adjustment and after excluding first five years of follow-up 43](#_Toc106108024)

# eTable 1: Selected baseline characteristics by smoking status in men and women

|  | **Men** | | | | **Women** | | | |
| --- | --- | --- | --- | --- | --- | --- | --- | --- |
|  | **Never-regular smokers (n = 53,917)** | **Ex-smokers (by choice) (n = 14,079)^†^** | **Regular smokers (n = 142,205)^‡^** | **All (n = 210,201)** | **Never-regular smokers (n = 292,718)** | **Ex-smokers (by choice) (n = 1,201)^†^** | **Regular smokers (n = 8,596)^‡^** | **All (n = 302,515)** |
| Age at baseline, years, n (%) |  |  |  |  |  |  |  |  |
| 30 - 39 | 9,296 (17.3) | 1,062 (8.0) | 19,205 (13.6) | 29,563 (14.1) | 47,701 (16.3) | 18 (3.6) | 331 (6.9) | 48,050 (15.9) |
| 40 - 49 | 14,035 (26.4) | 3,050 (21.6) | 42,133 (29.6) | 59,218 (28.0) | 92,244 (31.6) | 103 (11.3) | 1,204 (15.0) | 93,551 (31.0) |
| 50 - 59 | 14,183 (26.7) | 4,382 (32.0) | 45,186 (31.6) | 63,751 (30.3) | 91,180 (31.2) | 268 (19.7) | 2,417 (22.5) | 93,865 (31.0) |
| 60 - 69 | 11,168 (20.5) | 3,698 (26.1) | 26,474 (18.6) | 41,340 (19.8) | 46,637 (15.9) | 513 (44.1) | 3,263 (36.7) | 50,413 (16.6) |
| 70 - 79 | 5,235 (9.1) | 1,887 (12.3) | 9,207 (6.6) | 16,329 (7.8) | 14,956 (5.1) | 299 (21.3) | 1,381 (18.8) | 16,636 (5.5) |
| Mean (SD) | 52.8 (11.7) | 56.1 (10.9) | 52.5 (10.5) | 52.9 (10.9) | 51.2 (10.4) | 61.9 (8.9) | 59.5 (10.0) | 51.4 (10.5) |
| Urban, n (%) | 23,196 (42.5) | 6,033 (40.3) | 39,762 (28.0) | 68,991 (32.7) | 100,207 (34.6) | 630 (43.9) | 3,094 (36.9) | 103,931 (34.6) |
| Education, n (%) |  |  |  |  |  |  |  |  |
| No formal school | 3,599 (7.4) | 1,206 (7.1) | 13,856 (8.8) | 18,661 (8.3) | 73,095 (25.9) | 421 (32.4) | 2,999 (26.7) | 76,515 (26.1) |
| Primary school | 14,657 (29.6) | 4,515 (31.2) | 50,940 (33.6) | 70,112 (32.4) | 91,044 (31.5) | 494 (32.3) | 3,535 (35.0) | 95,073 (31.6) |
| Middle or high school | 27,996 (51.2) | 7,005 (53.1) | 69,890 (51.4) | 104,891 (51.2) | 115,274 (38.1) | 261 (33.4) | 1,954 (36.4) | 117,489 (37.9) |
| College or university | 7,665 (11.8) | 1,353 (8.5) | 7,519 (6.2) | 16,537 (8.1) | 13,305 (4.5) | 25 (1.9) | 108 (1.9) | 13,438 (4.4) |
| Alcohol drinking, n (%) |  |  |  |  |  |  |  |  |
| Never | 14,016 (26.4) | 2,499 (17.7) | 26,262 (17.8) | 42,777 (19.9) | 187,976 (64.1) | 530 (37.1) | 3,824 (45.0) | 192,330 (63.7) |
| Occasional | 25,688 (46.1) | 4,827 (36.6) | 48,715 (35.1) | 79,230 (38.2) | 97,630 (33.3) | 473 (45.4) | 3,180 (42.2) | 101,283 (33.4) |
| Regular | 14,213 (27.5) | 6,753 (45.7) | 67,228 (47.1) | 88,194 (41.9) | 7,112 (2.6) | 198 (17.5) | 1,592 (12.7) | 8,902 (3.0) |
| Body Mass Index, kg/m² | 23.8 (3.2) | 24.3 (3.2) | 23.2 (3.2) | 23.5 (3.2) | 23.8 (3.4) | 24.6 (3.7) | 23.9 (3.9) | 23.8 (3.5) |
| SBP, mmHg | 132.8 (19.7) | 134.1 (20.2) | 132.0 (20.1) | 132.3 (20.0) | 130.5 (22.0) | 128.1 (21.5) | 127.4 (23.0) | 130.4 (22.0) |
| Self-reported disease history, n (%) |  |  |  |  |  |  |  |  |
| CHD | 1,656 (2.5) | 557 (2.4) | 3,504 (2.6) | 5,717 (2.6) | 9,110 (3.3) | 115 (5.4) | 530 (3.7) | 9,755 (3.3) |
| Stroke or TIA | 1,318 (2.0) | 373 (1.7) | 3,220 (2.4) | 4,911 (2.2) | 3,657 (1.4) | 45 (5.4) | 271 (2.2) | 3,973 (1.4) |
| Rheumatic heart disease | 56 (0.1) | 17 (0.1) | 137 (0.1) | 210 (0.1) | 681 (0.2) | 6 (0.2) | 39 (0.1) | 726 (0.2) |
| Emphysema/bronchitis | 1,595 (3.0) | 444 (2.6) | 4,503 (3.0) | 6,542 (2.9) | 6,234 (2.2) | 62 (2.0) | 450 (2.7) | 6,746 (2.3) |
| Asthma | 380 (0.7) | 80 (0.5) | 754 (0.6) | 1,214 (0.6) | 1,509 (0.5) | 13 (1.5) | 70 (1.0) | 1,592 (0.5) |
| Diabetes | 1,856 (3.1) | 633 (3.3) | 3,635 (2.8) | 6,124 (2.9) | 9,486 (3.4) | 86 (3.9) | 466 (4.5) | 10,038 (3.4) |
| Values are mean (SD) unless otherwise stated. Means and percentages are directly standardised to age and study area structure of the CKB study population. Abbreviations: BMI = Body mass index; SBP = Systolic blood pressure; CHD = Coronary heart disease; TIA = Transient Ischaemic Attack. ^†^ Include only those who had quit voluntarily (i.e. not due to ill health). ^‡^ Include current smokers and ex-smokers who had quit due to ill health. | | | | | | | | |

# eFigure 1: Adjusted HRs for ICD-10 chapter-specific incidence associated with ever-regular smoking


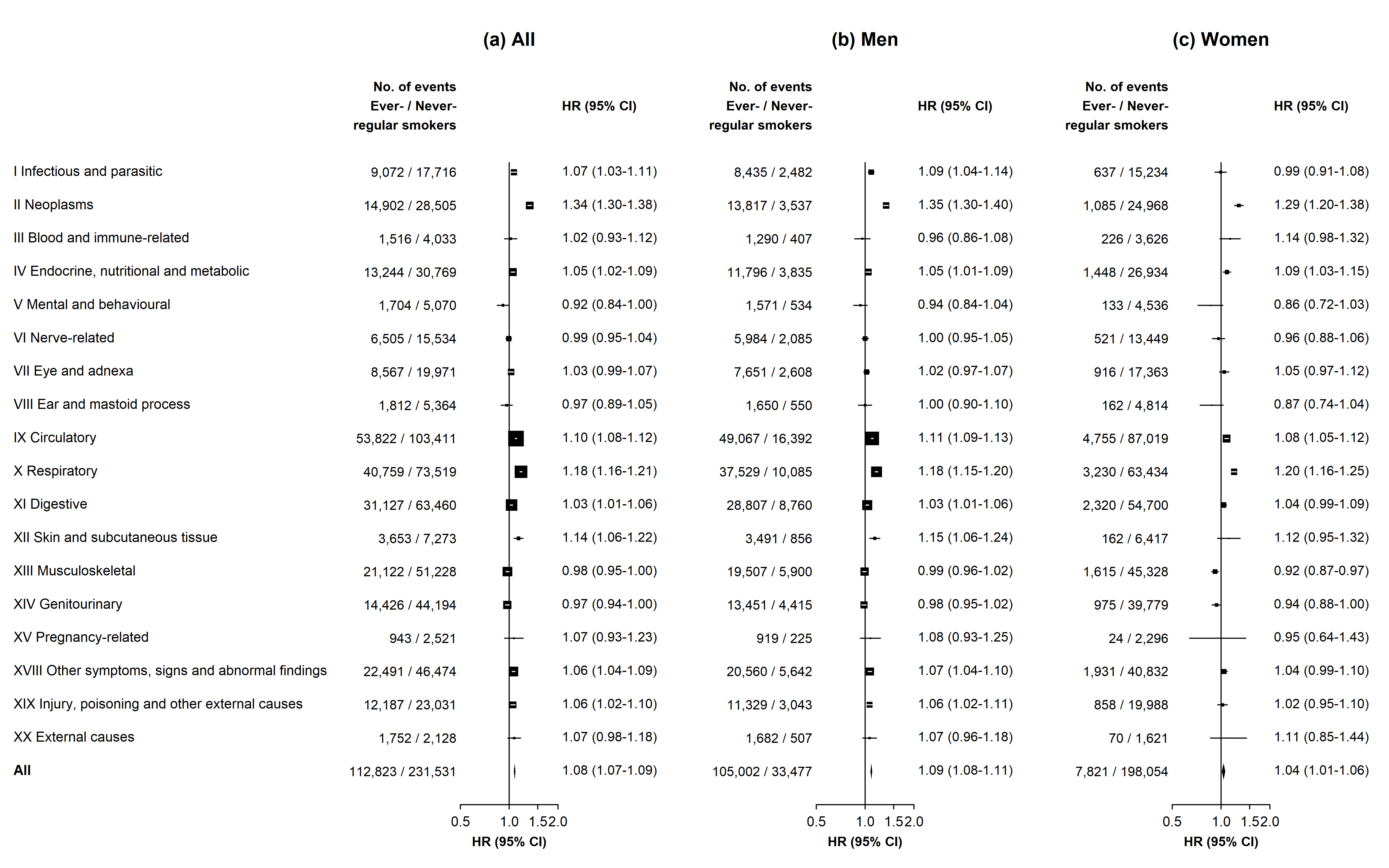


# eFigure 2: Adjusted HRs for ICD-10 chapter-specific mortality associated with ever-regular smoking


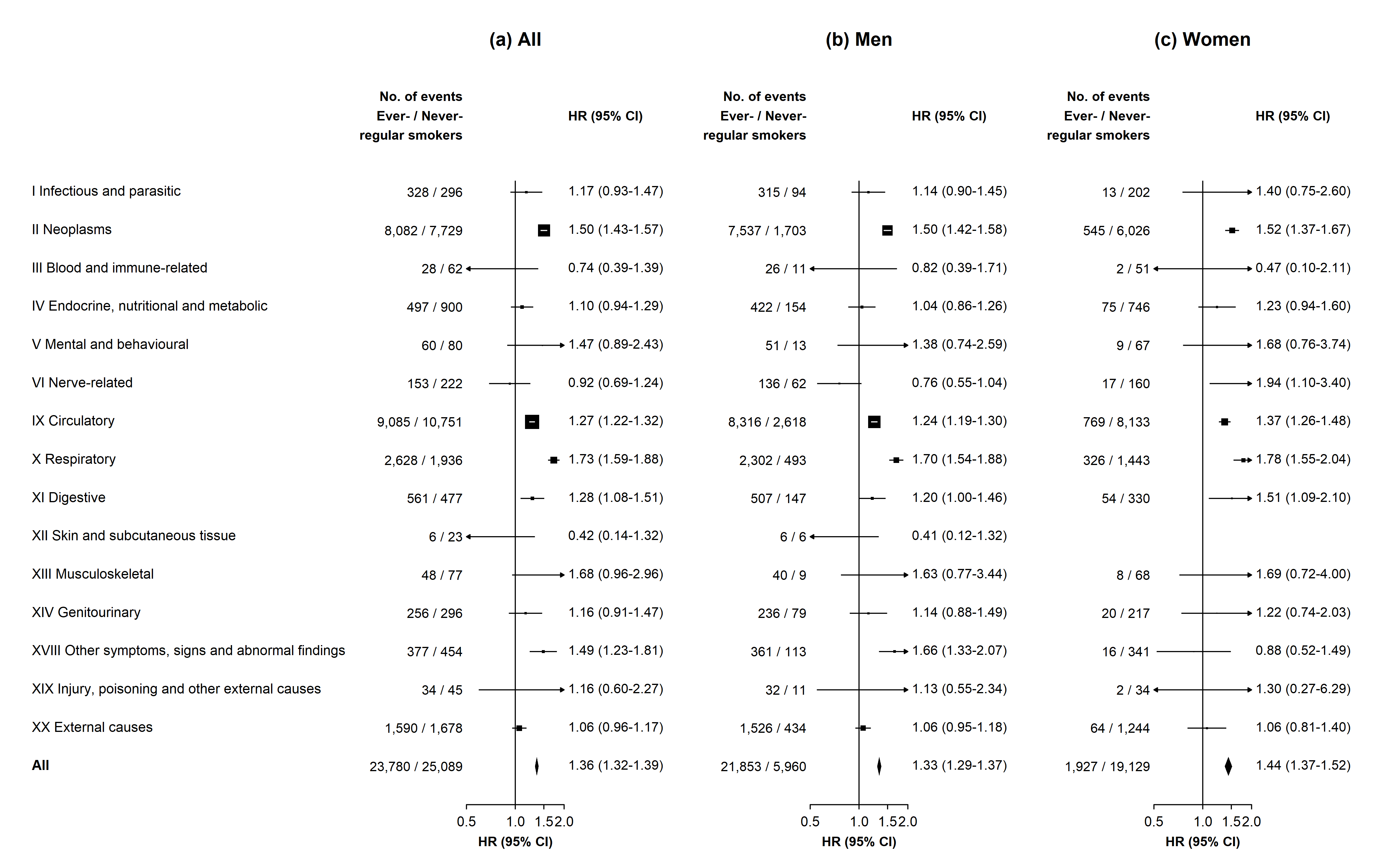


# eTable 2: Adjusted HRs for risks of any disease incidence and overall mortality associated with smoking with and without excluding those with prior cancer reported at baseline, in rural men

|  | **All disease incidence** | | | | **All-cause mortality** | | | |
| --- | --- | --- | --- | --- | --- | --- | --- | --- |
|  | **Prior cancer excluded** | | **Prior cancer not excluded** | | **Prior cancer excluded** | | **Prior cancer not excluded** | |
| **Smoking category** | **Number of events** | **HR (95% CI)^†^** | **Number of events** | **HR (95% CI)^†^** | **Number of events** | **HR (95% CI)^†^** | **Number of events** | **HR (95% CI)^†^** |
| Never-regular smoker | 20,102 | 1.00 (0.99, 1.01) | 20,217 | 1.00 (0.99, 1.01) | 3,627 | 1.00 (0.97, 1.03) | 3,687 | 1.00 (0.97, 1.03) |
| Ever-regular smoker | 76,219 | 1.05 (1.03, 1.06) | 76,625 | 1.05 (1.03, 1.06) | 15,540 | 1.25 (1.20, 1.30) | 15,764 | 1.25 (1.20, 1.30) |
| Ex-smoker (by choice)^‡^ | 5,634 | 1.05 (1.02, 1.08) | 5,662 | 1.05 (1.02, 1.08) | 1,129 | 1.07 (1.01, 1.13) | 1,143 | 1.07 (1.01, 1.13) |
| Regular smoker | 70,585 | 1.05 (1.04, 1.06) | 70,963 | 1.05 (1.04, 1.06) | 14,411 | 1.27 (1.25, 1.29) | 14,621 | 1.27 (1.25, 1.29) |
| Ex-smoker (ill health) | 6,857 | 1.20 (1.17, 1.23) | 7,066 | 1.20 (1.18, 1.23) | 2,105 | 1.53 (1.46, 1.60) | 2,231 | 1.56 (1.50, 1.63) |
| Current smoker | 63,728 | 1.03 (1.02, 1.04) | 63,897 | 1.03 (1.02, 1.04) | 12,306 | 1.23 (1.21, 1.25) | 12,390 | 1.22 (1.20, 1.25) |
| Age began smoking (years)^*^ |  |  |  |  |  |  |  |  |
| 25+ | 21,225 | 1.01 (0.99, 1.02) | 21,312 | 1.01 (0.99, 1.02) | 4,518 | 1.10 (1.06, 1.13) | 4,564 | 1.09 (1.06, 1.13) |
| 18-24 | 36,928 | 1.06 (1.05, 1.07) | 37,139 | 1.06 (1.05, 1.07) | 7,036 | 1.31 (1.28, 1.34) | 7,162 | 1.31 (1.28, 1.34) |
| <18 | 12,432 | 1.09 (1.07, 1.11) | 12,512 | 1.09 (1.07, 1.11) | 2,857 | 1.52 (1.46, 1.58) | 2,895 | 1.51 (1.46, 1.57) |
| No. smoked (cig/day)^*^ |  |  |  |  |  |  |  |  |
| <15 | 24,298 | 1.02 (1.01, 1.04) | 24,431 | 1.02 (1.01, 1.04) | 5,887 | 1.27 (1.24, 1.30) | 5,965 | 1.27 (1.24, 1.30) |
| 15-24 | 32,222 | 1.05 (1.04, 1.06) | 32,405 | 1.05 (1.04, 1.06) | 5,814 | 1.23 (1.20, 1.27) | 5,912 | 1.24 (1.20, 1.27) |
| 25+ | 14,065 | 1.09 (1.07, 1.10) | 14,127 | 1.09 (1.07, 1.10) | 2,710 | 1.33 (1.28, 1.38) | 2,744 | 1.33 (1.28, 1.38) |
| ^†^ All analyses were stratified by study area and 5-year age-at-risk group and adjusted for education and alcohol drinking. ^‡^ Only include those who had quit voluntarily (i.e. not due to ill health). ^*^ Include current smokers and ex-smokers who had quit due to ill health. | | | | | | | | |

# eTable 3: Adjusted HRs for risks of any disease incidence and overall mortality associated with smoking with and without excluding those with prior cancer reported at baseline, in urban men

|  | **All disease incidence** | | | | **All-cause mortality** | | | |
| --- | --- | --- | --- | --- | --- | --- | --- | --- |
|  | **Prior cancer excluded** | | **Prior cancer not excluded** | | **Prior cancer excluded** | | **Prior cancer not excluded** | |
| **Smoking category** | **Number of events** | **HR (95% CI)^†^** | **Number of events** | **HR (95% CI)^†^** | **Number of events** | **HR (95% CI)^†^** | **Number of events** | **HR (95% CI)^†^** |
| Never-regular smoker | 13,165 | 1.00 (0.98, 1.02) | 13,255 | 1.00 (0.98, 1.02) | 2,229 | 1.00 (0.96, 1.04) | 2,264 | 1.00 (0.96, 1.04) |
| Ever-regular smoker | 28,139 | 1.17 (1.15, 1.20) | 28,356 | 1.17 (1.15, 1.20) | 5,950 | 1.50 (1.42, 1.57) | 6,054 | 1.50 (1.42, 1.58) |
| Ex-smoker (by choice)^‡^ | 4,031 | 1.06 (1.03, 1.10) | 4,063 | 1.06 (1.03, 1.09) | 828 | 1.06 (0.99, 1.14) | 841 | 1.06 (0.99, 1.14) |
| Regular smoker | 24,108 | 1.20 (1.18, 1.21) | 24,293 | 1.20 (1.18, 1.21) | 5,122 | 1.61 (1.57, 1.66) | 5,213 | 1.62 (1.57, 1.66) |
| Ex-smoker (ill health) | 3,716 | 1.31 (1.26, 1.35) | 3,817 | 1.32 (1.28, 1.36) | 1,140 | 1.63 (1.54, 1.73) | 1,198 | 1.67 (1.58, 1.77) |
| Current smoker | 20,392 | 1.18 (1.16, 1.19) | 20,476 | 1.17 (1.16, 1.19) | 3,982 | 1.61 (1.55, 1.66) | 4,015 | 1.60 (1.55, 1.65) |
| Age began smoking (years)^*^ |  |  |  |  |  |  |  |  |
| 25+ | 7,209 | 1.12 (1.10, 1.15) | 7,274 | 1.13 (1.10, 1.15) | 1,654 | 1.41 (1.34, 1.48) | 1,687 | 1.42 (1.35, 1.49) |
| 18-24 | 12,925 | 1.21 (1.19, 1.23) | 13,012 | 1.21 (1.19, 1.23) | 2,552 | 1.65 (1.59, 1.72) | 2,596 | 1.66 (1.59, 1.73) |
| <18 | 3,974 | 1.31 (1.27, 1.36) | 4,007 | 1.32 (1.28, 1.36) | 916 | 2.05 (1.92, 2.20) | 930 | 2.05 (1.92, 2.19) |
| No. smoked (cig/day)^*^ |  |  |  |  |  |  |  |  |
| <15 | 8,629 | 1.12 (1.09, 1.14) | 8,690 | 1.12 (1.10, 1.14) | 1,845 | 1.43 (1.37, 1.50) | 1,875 | 1.43 (1.37, 1.50) |
| 15-24 | 11,384 | 1.21 (1.19, 1.23) | 11,474 | 1.21 (1.19, 1.23) | 2,357 | 1.67 (1.60, 1.74) | 2,397 | 1.67 (1.60, 1.74) |
| 25+ | 4,095 | 1.34 (1.30, 1.38) | 4,129 | 1.34 (1.30, 1.38) | 920 | 1.93 (1.80, 2.06) | 941 | 1.94 (1.81, 2.06) |
| ^†^ All analyses were stratified by study area and 5-year age-at-risk group and adjusted for education and alcohol drinking. ^‡^ Only include those who had quit voluntarily (i.e. not due to ill health). ^*^ Include current smokers and ex-smokers who had quit due to ill health. | | | | | | | | |

# eTable 4: Number of morbidity and mortality events by ICD-10 chapter and their overall associations with ever-regular smoking, in men

|  | | | | | | **No. of significant associations** | | | | | | | |
| --- | --- | --- | --- | --- | --- | --- | --- | --- | --- | --- | --- | --- | --- |
|  | | | | | | **Without FDR adjustment** | | | | **With FDR adjustment** | | | |
|  | | **No. of diseases** | | **No. of cases** | | **Positive** | | **Inverse** | | **Positive** | | **Inverse** | |
| **ICD-10 Chapter** | | **Incidence** | **Death** | **Incidence** | **Death** | **Incidence** | **Death** | **Incidence** | **Death** | **Incidence** | **Death** | **Incidence** | **Death** |
| I | Infectious and parasitic | 15 | 4 | 11,729 | 409 | 3 | 0 | 0 | 0 | 2 | 0 | 0 | 0 |
| II | Neoplasms | 46 | 16 | 24,599 | 9,240 | 17 | 8 | 0 | 0 | 14 | 6 | 0 | 0 |
| III | Blood and immune-related | 6 | 1 | 1,904 | 37 | 0 | 0 | 0 | 0 | 0 | 0 | 0 | 0 |
| IV | Endocrine, nutritional and metabolic | 11 | 2 | 16,705 | 576 | 0 | 0 | 0 | 0 | 0 | 0 | 0 | 0 |
| V | Mental and behavioural | 9 | 4 | 2,506 | 64 | 0 | 0 | 0 | 0 | 0 | 0 | 0 | 0 |
| VI | Nerve-related | 13 | 2 | 8,445 | 198 | 1 | 0 | 1 | 1 | 0 | 0 | 1 | 0 |
| VII | Eye and adnexa | 15 | - | 15,565 | - | 2 | - | 1 | - | 2 | - | 1 | - |
| VIII | Ear and mastoid process | 4 | - | 2,241 | - | 0 | - | 0 | - | 0 | - | 0 | - |
| IX | Circulatory | 40 | 17 | 120,010 | 10,934 | 12 | 7 | 1 | 1 | 12 | 6 | 1 | 0 |
| X | Respiratory | 21 | 9 | 74,960 | 2,795 | 13 | 3 | 0 | 0 | 13 | 3 | 0 | 0 |
| XI | Digestive | 45 | 2 | 52,206 | 654 | 8 | 1 | 4 | 0 | 2 | 0 | 1 | 0 |
| XII | Skin and subcutaneous tissue | 8 | 1 | 4,901 | 12 | 1 | 0 | 0 | 0 | 0 | 0 | 0 | 0 |
| XIII | Musculoskeletal | 26 | 1 | 33,722 | 49 | 0 | 0 | 4 | 0 | 0 | 0 | 2 | 0 |
| XIV | Genitourinary | 27 | 2 | 22,622 | 315 | 1 | 0 | 3 | 0 | 1 | 0 | 2 | 0 |
| XV | Pregnancy-related | 4 | - | 1,243 | - | 0 | - | 0 | - | 0 | - | 0 | - |
| XVIII | Other symptoms, signs and abnormal findings | 30 | 3 | 32,679 | 474 | 9 | 2 | 0 | 0 | 4 | 2 | 0 | 0 |
| XIX | Injury, poisoning and other external causes | 30 | 1 | 16,768 | 43 | 4 | 0 | 0 | 0 | 0 | 0 | 0 | 0 |
| XX | External causes | 8 | 7 | 2,191 | 1,960 | 0 | 1 | 1 | 1 | 0 | 0 | 0 | 0 |
|  | **Total** | **358** | **72** | **444,996** | **27,760** | **71** | **22** | **15** | **3** | **50** | **17** | **8** | **0** |

# eTable 5: Number of morbidity and mortality events by ICD-10 chapter and their overall associations with ever-regular smoking, in women

|  | | | | | | **No. of significant associations** | | | | | | | |
| --- | --- | --- | --- | --- | --- | --- | --- | --- | --- | --- | --- | --- | --- |
|  | | | | | | **Without FDR adjustment** | | | | **With FDR adjustment** | | | |
|  | | **No. of diseases** | | **No. of cases** | | **Positive** | | **Inverse** | | **Positive** | | **Inverse** | |
| **ICD-10 Chapter** | | **Incidence** | **Death** | **Incidence** | **Death** | **Incidence** | **Death** | **Incidence** | **Death** | **Incidence** | **Death** | **Incidence** | **Death** |
| I | Infectious and parasitic | 18 | 4 | 16,831 | 215 | 1 | 1 | 0 | 0 | 0 | 0 | 0 | 0 |
| II | Neoplasms | 59 | 19 | 34,961 | 6,571 | 4 | 2 | 0 | 0 | 1 | 1 | 0 | 0 |
| III | Blood and immune-related | 7 | 1 | 4,241 | 53 | 0 | 0 | 0 | 0 | 0 | 0 | 0 | 0 |
| IV | Endocrine, nutritional and metabolic | 13 | 2 | 31,104 | 821 | 4 | 0 | 0 | 0 | 4 | 0 | 0 | 0 |
| V | Mental and behavioural | 10 | 3 | 5,598 | 76 | 0 | 1 | 1 | 0 | 0 | 0 | 0 | 0 |
| VI | Nerve-related | 18 | 2 | 14,593 | 177 | 0 | 1 | 0 | 0 | 0 | 0 | 0 | 0 |
| VII | Eye and adnexa | 20 | - | 27,595 | - | 0 | - | 1 | - | 0 | - | 0 | - |
| VIII | Ear and mastoid process | 5 | - | 5,076 | - | 0 | - | 0 | - | 0 | - | 0 | - |
| IX | Circulatory | 41 | 16 | 159,296 | 8,902 | 9 | 6 | 1 | 0 | 6 | 5 | 0 | 0 |
| X | Respiratory | 19 | 7 | 100,674 | 1,769 | 11 | 3 | 0 | 0 | 11 | 3 | 0 | 0 |
| XI | Digestive | 47 | 2 | 80,599 | 384 | 4 | 0 | 1 | 0 | 1 | 0 | 0 | 0 |
| XII | Skin and subcutaneous tissue | 8 | 1 | 7,360 | 17 | 0 | 0 | 0 | 0 | 0 | 0 | 0 | 0 |
| XIII | Musculoskeletal | 32 | 1 | 65,380 | 76 | 0 | 0 | 3 | 0 | 0 | 0 | 0 | 0 |
| XIV | Genitourinary | 44 | 2 | 55,923 | 237 | 2 | 0 | 3 | 0 | 0 | 0 | 0 | 0 |
| XV | Pregnancy-related | 6 | 1 | 2,498 | 1 | 0 | 0 | 0 | 0 | 0 | 0 | 0 | 0 |
| XVIII | Other symptoms, signs and abnormal findings | 31 | 2 | 54,566 | 357 | 3 | 0 | 1 | 0 | 1 | 0 | 0 | 0 |
| XIX | Injury, poisoning and other external causes | 34 | 1 | 24,292 | 36 | 1 | 0 | 0 | 0 | 0 | 0 | 0 | 0 |
| XX | External causes | 7 | 5 | 1,698 | 1,308 | 2 | 1 | 0 | 0 | 0 | 0 | 0 | 0 |
|  | **Total** | **419** | **69** | **692,285** | **21,000** | **41** | **15** | **11** | **0** | **24** | **9** | **0** | **0** |

# eFigure 3: Adjusted HRs for incidence of specific types of infectious and parasitic diseases associated with ever-regular smoking in men and women combined


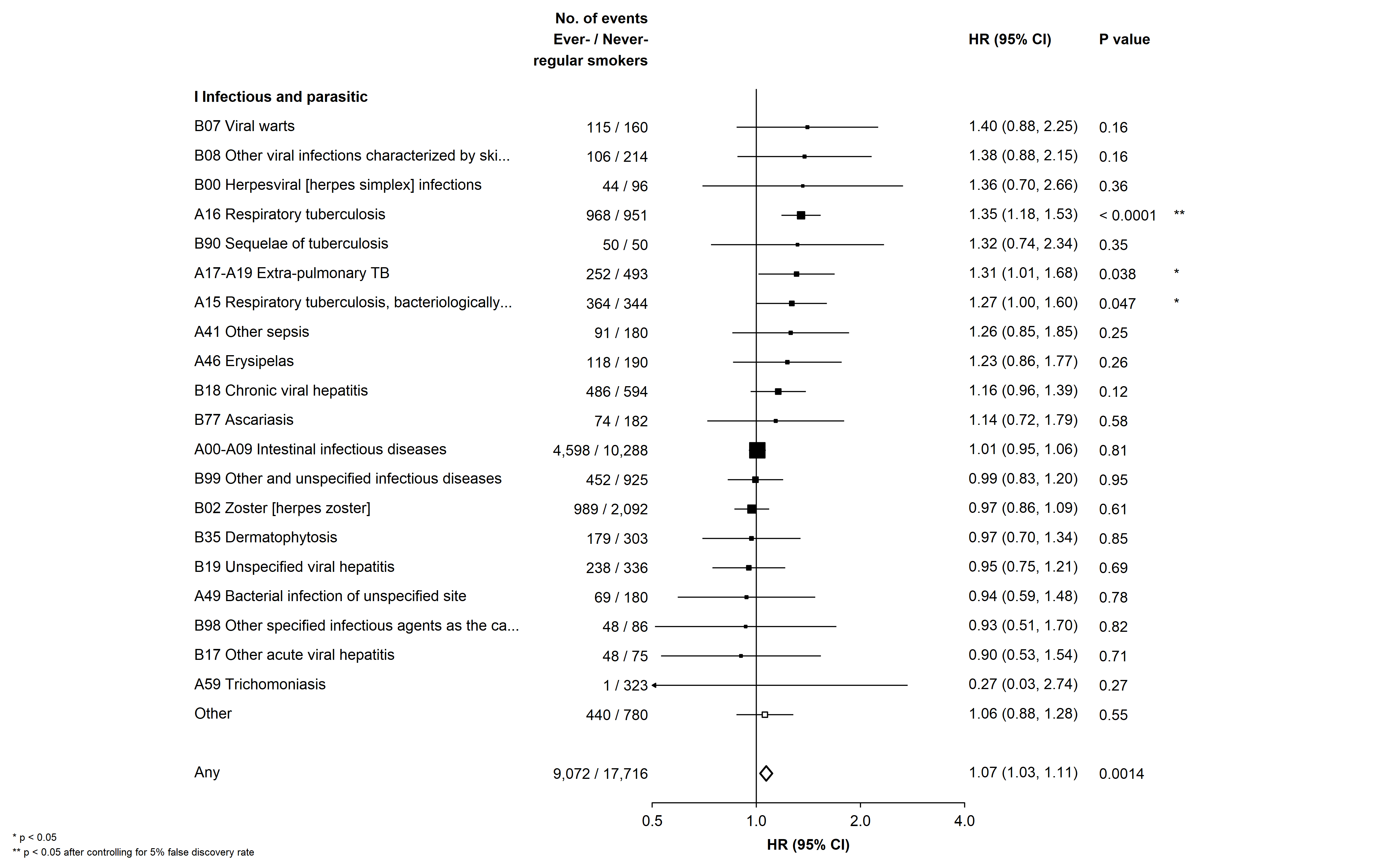


# eFigure 4: Adjusted HRs for incidence of specific types of neoplasms associated with ever-regular smoking in men and women combined


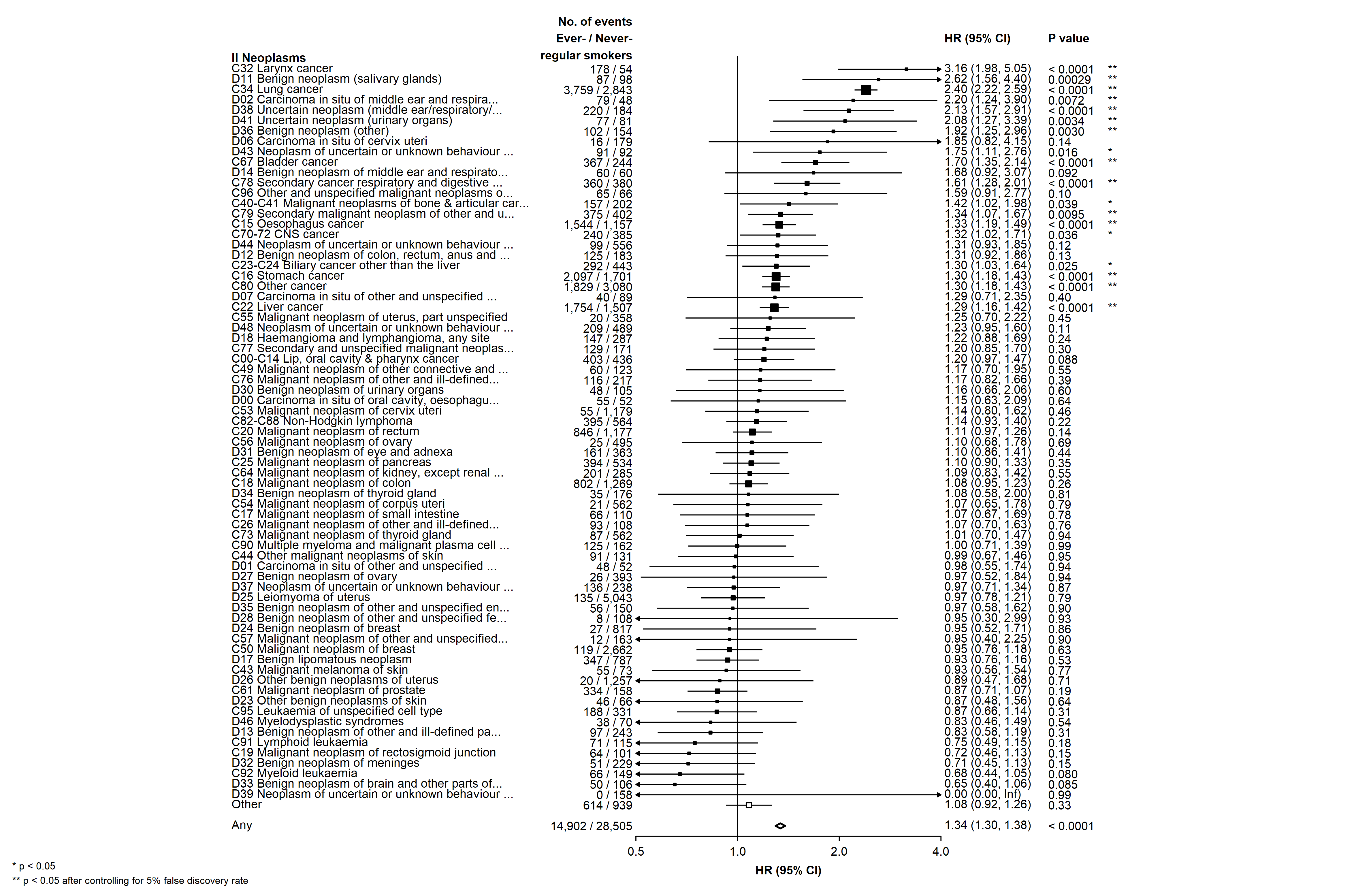


# eFigure 5: Adjusted HRs for incidence of specific types of blood and immune-related diseases associated with ever-regular smoking in men and women combined


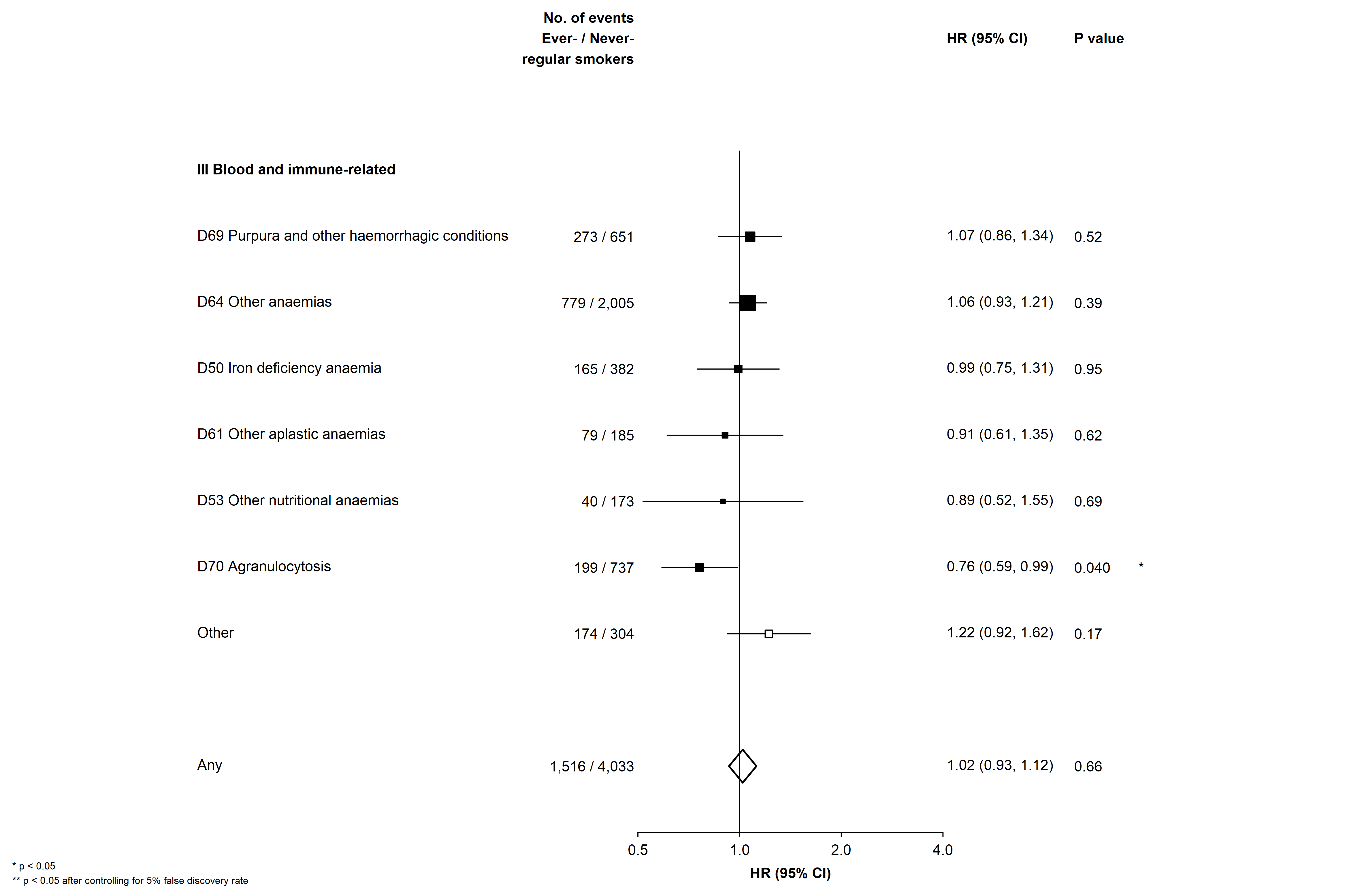


# eFigure 6: Adjusted HRs for incidence of specific types of endocrine, nutritional and metabolic diseases associated with ever-regular smoking in men and women combined


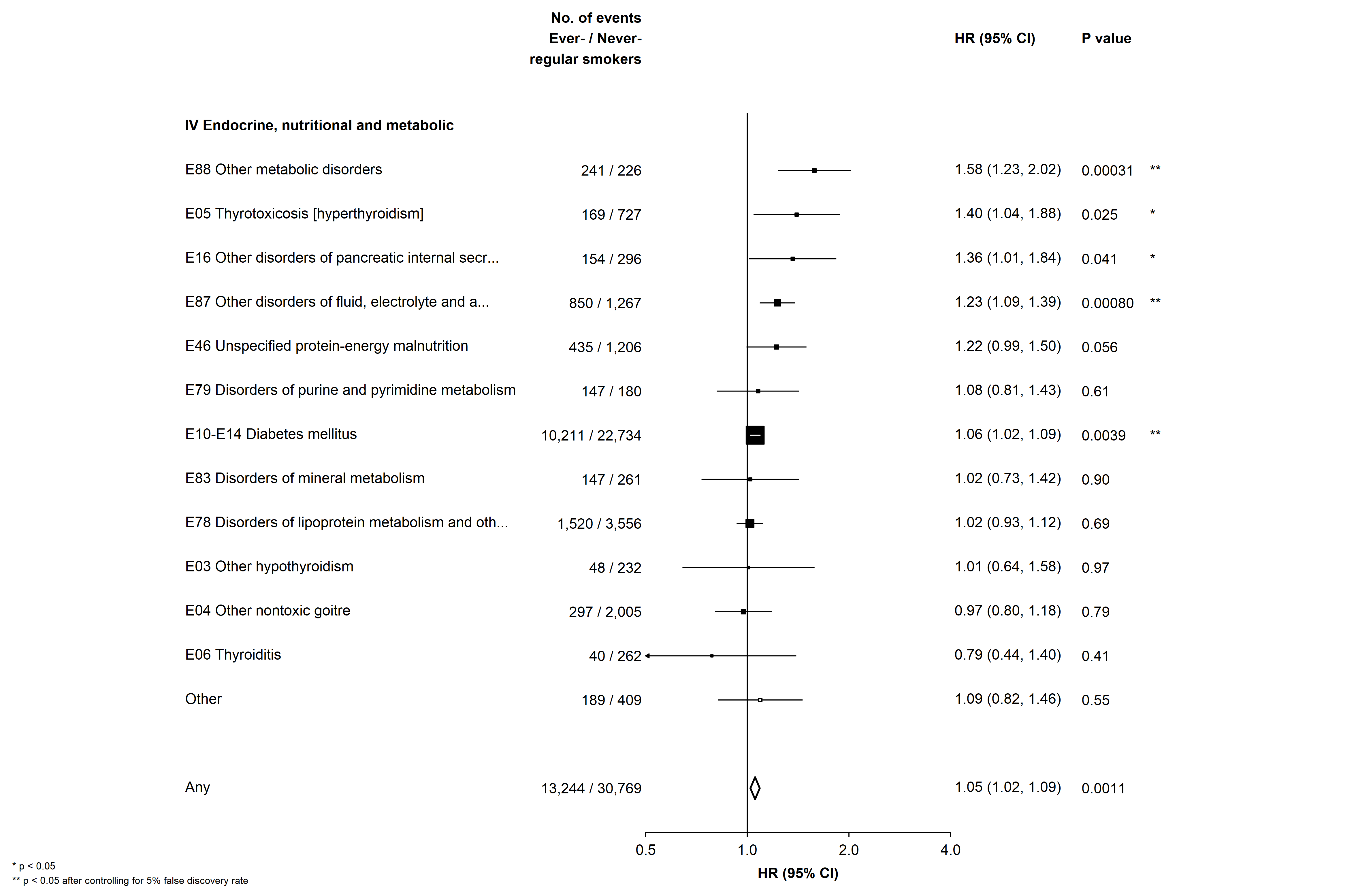


# eFigure 7: Adjusted HRs for incidence of specific types of mental and behavioural disorders associated with ever-regular smoking in men and women combined


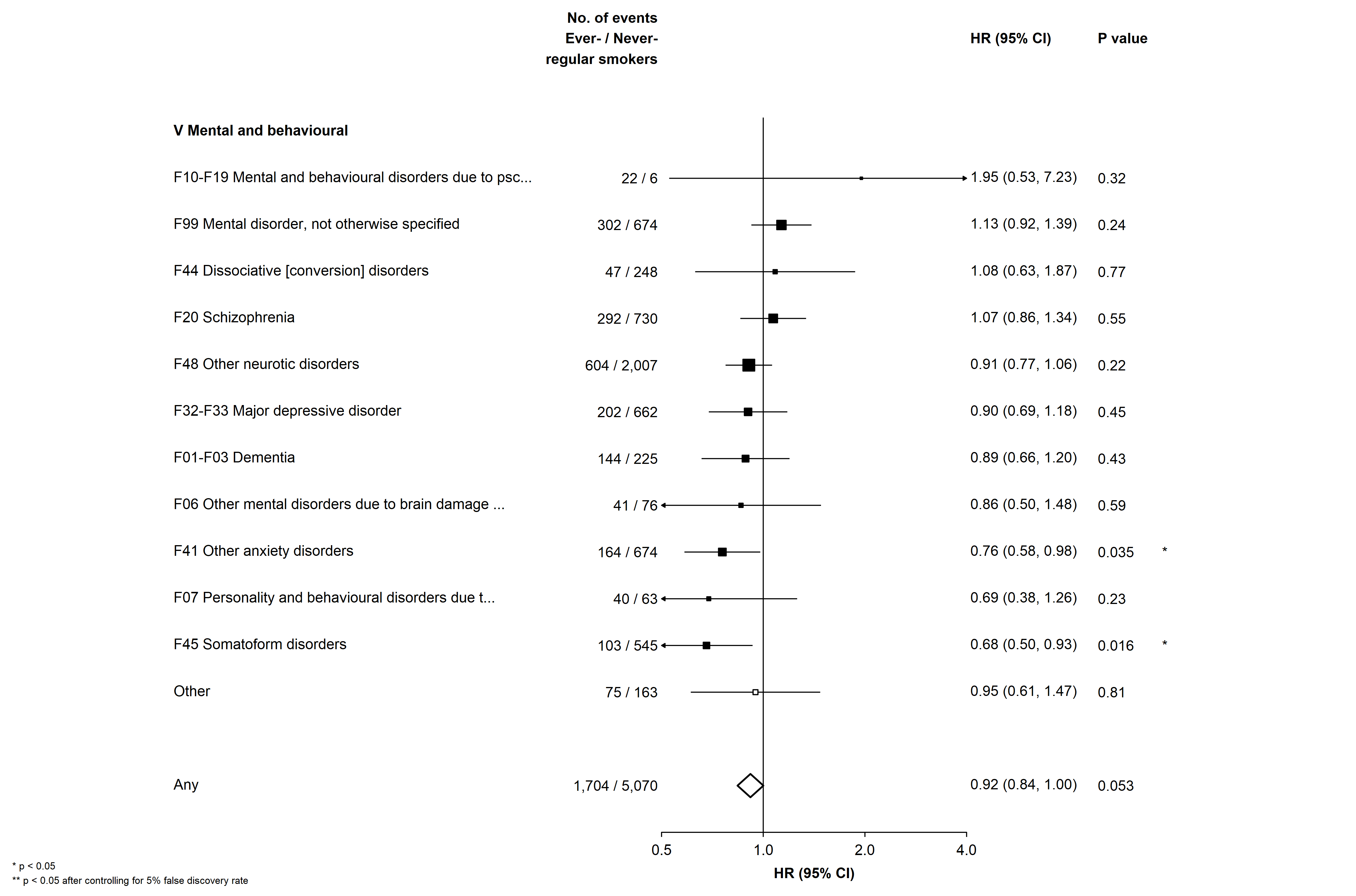


# eFigure 8: Adjusted HRs for incidence of specific types of nerve-related diseases associated with ever-regular smoking in men and women combined


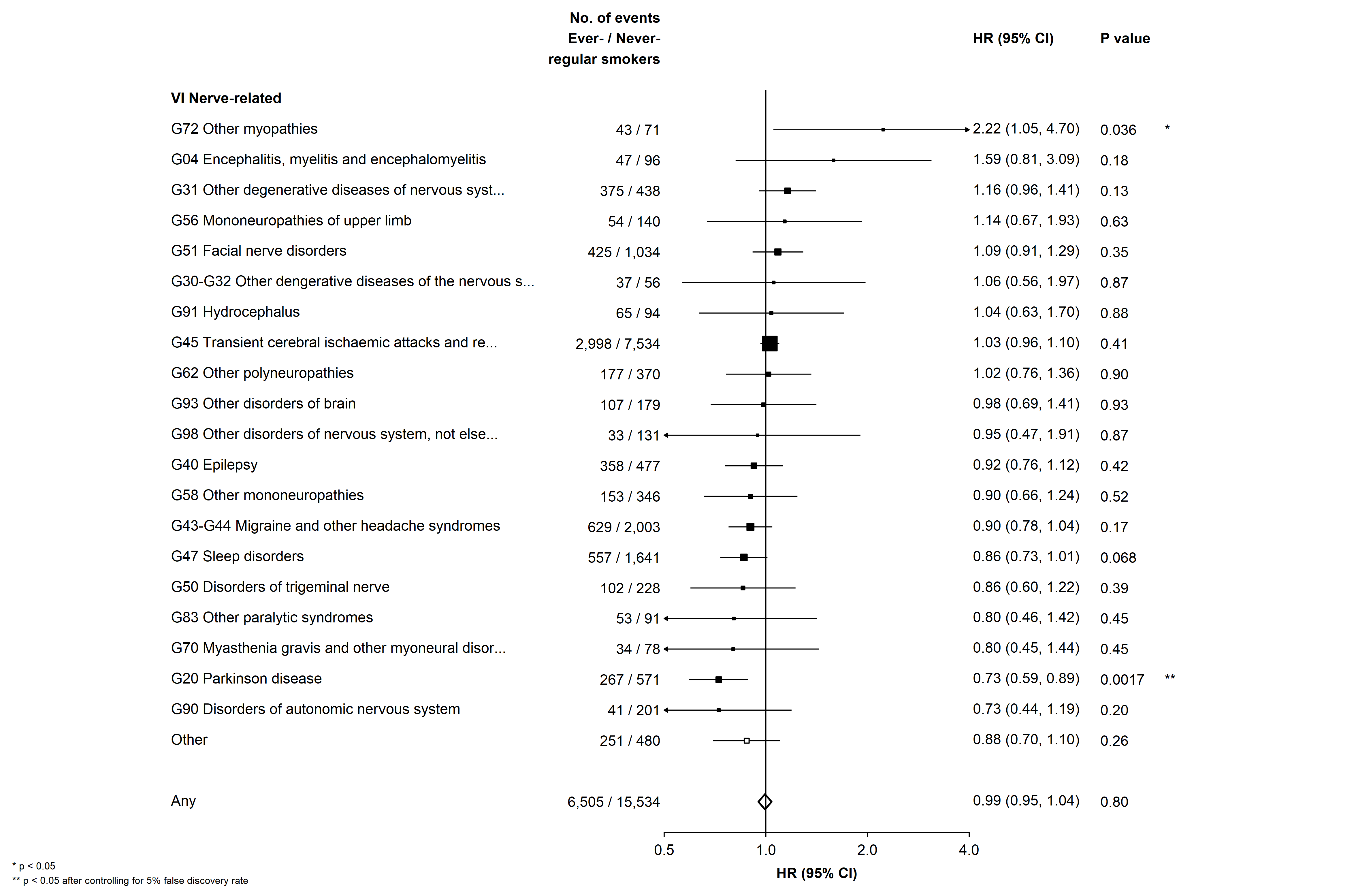


# eFigure 9: Adjusted HRs for incidence of specific types of eye and adnexa diseases associated with ever-regular smoking in men and women combined


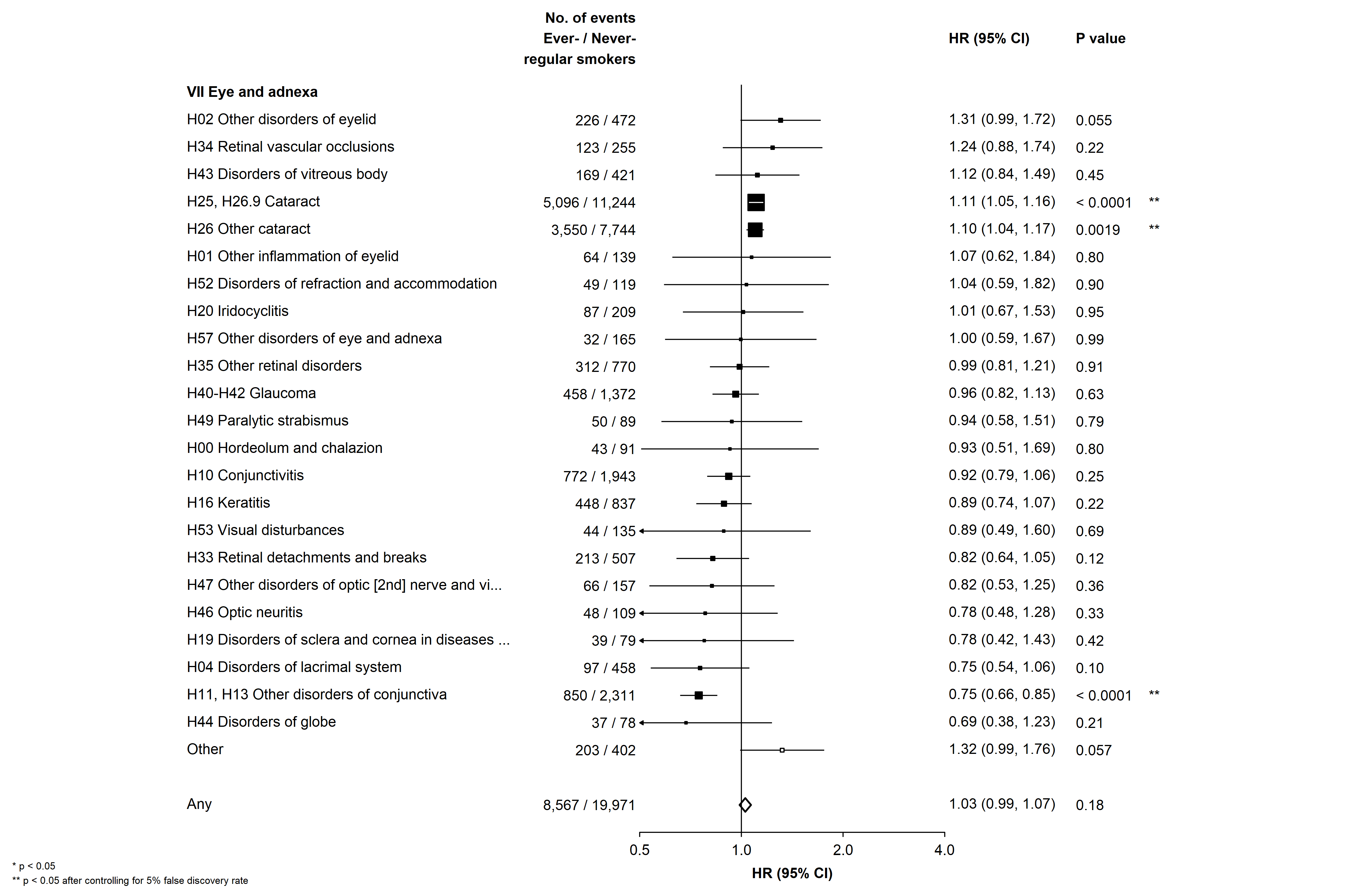


# eFigure 10: Adjusted HRs for incidence of specific types of ear and mastoid process diseases associated with ever-regular smoking in men and women combined


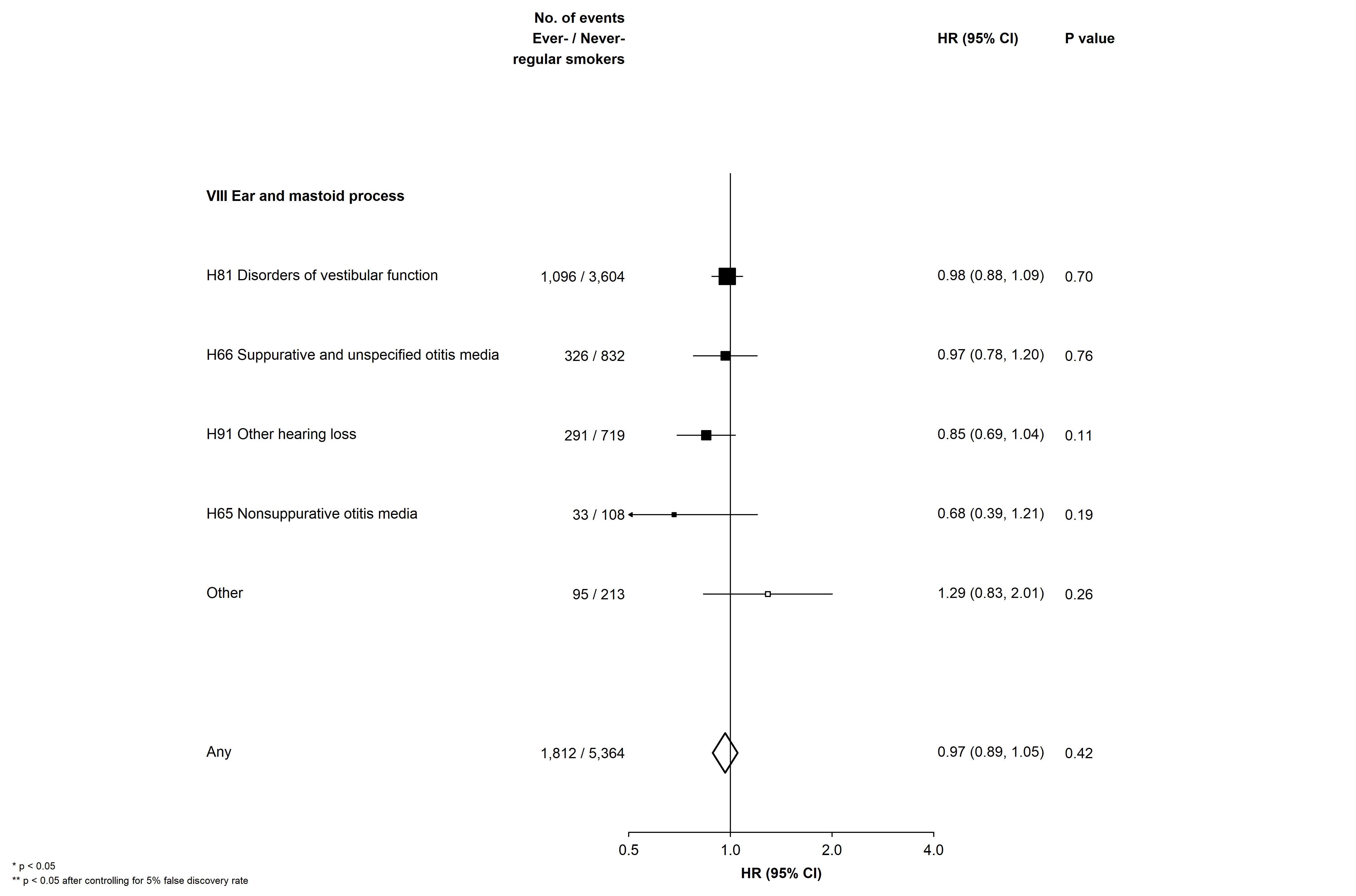


# eFigure 11: Adjusted HRs for incidence of specific types of circulatory diseases associated with ever-regular smoking in men and women combined


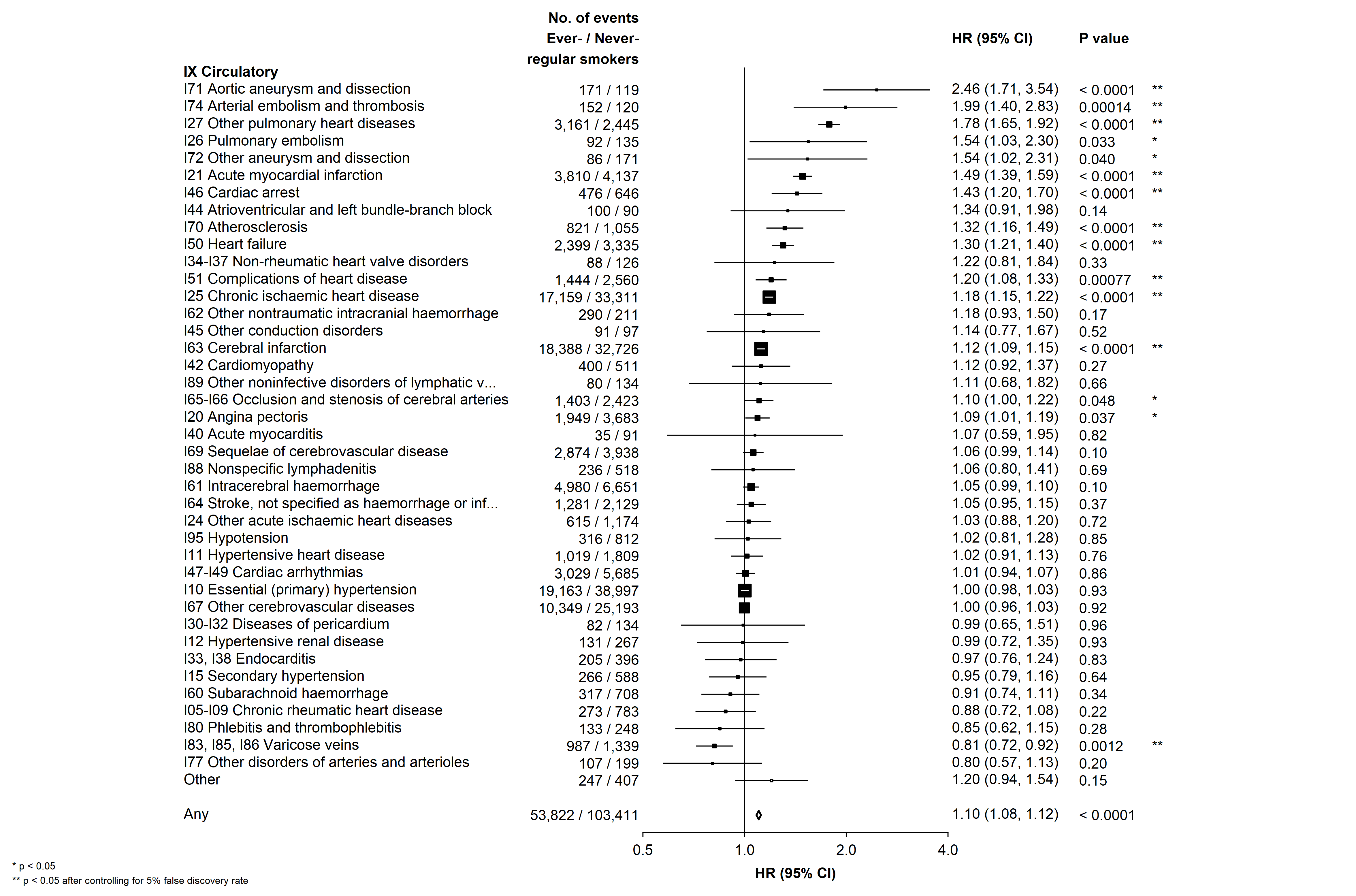


# eFigure 12: Adjusted HRs for incidence of specific types of respiratory diseases associated with ever-regular smoking in men and women combined


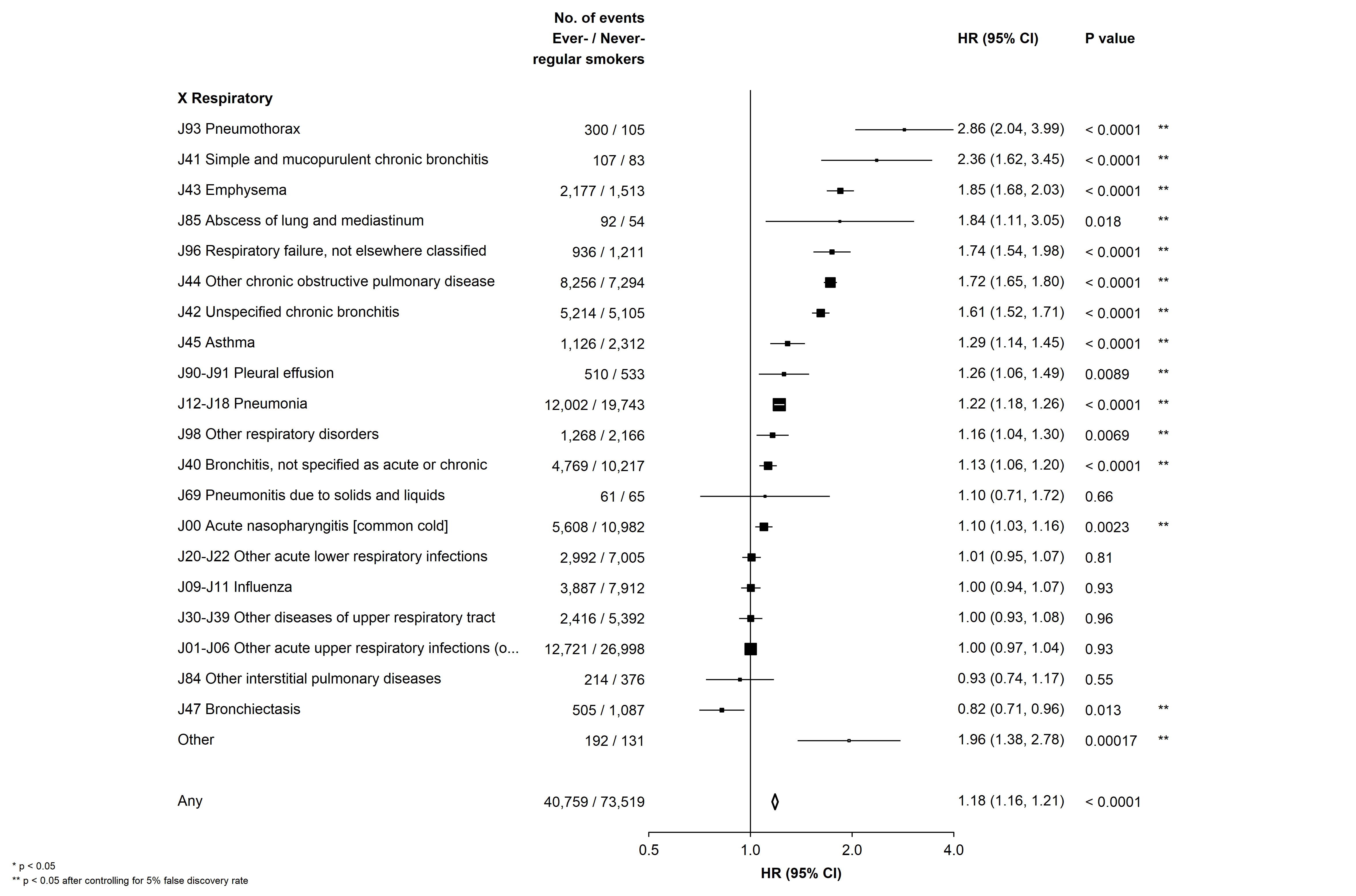


# eFigure 13: Adjusted HRs for incidence of specific types of digestive diseases associated with ever-regular smoking in men and women combined


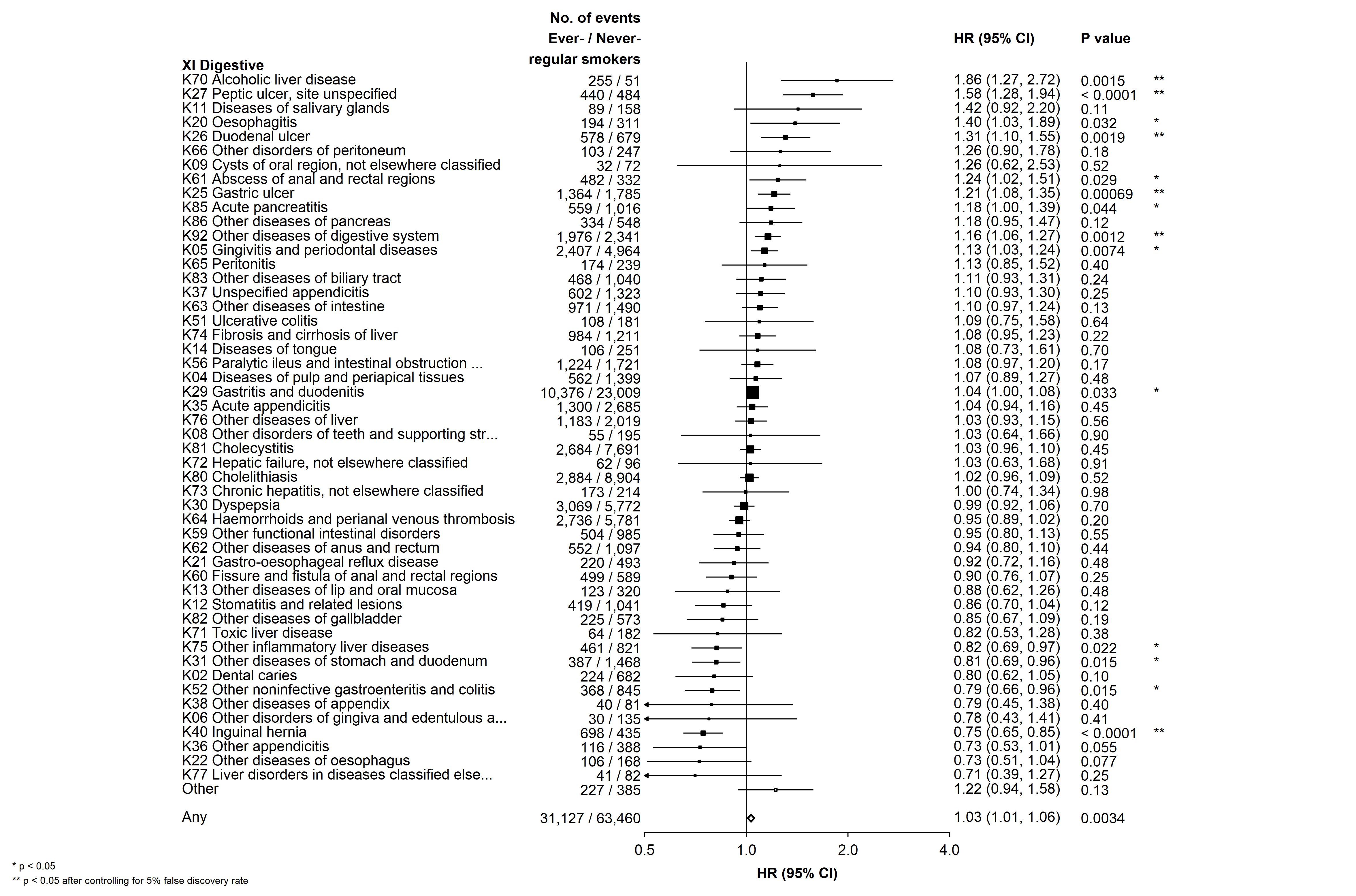


# eFigure 14: Adjusted HRs for incidence of specific types of skin and subcutaneous tissue diseases associated with ever-regular smoking in men and women combined


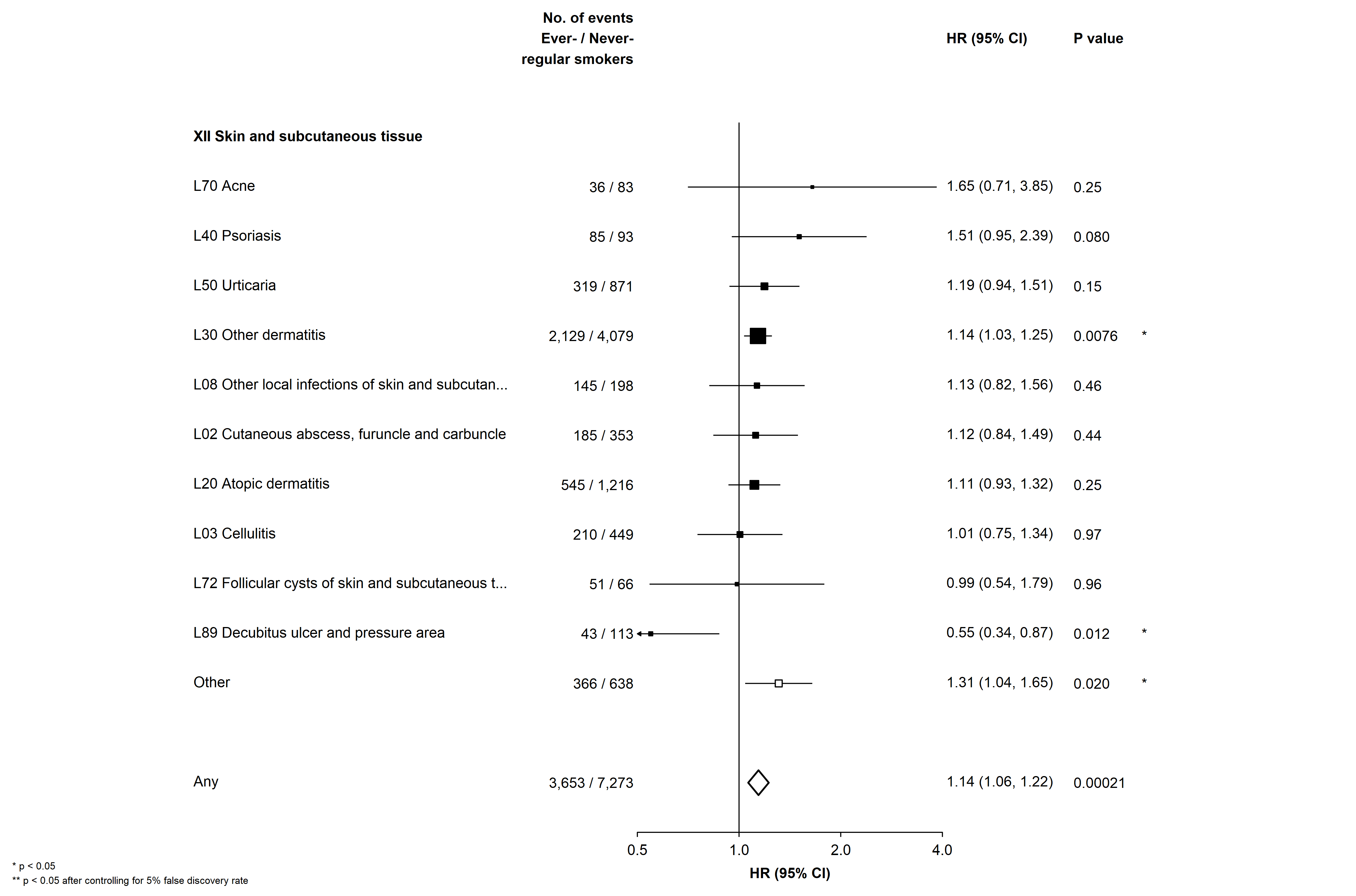


# eFigure 15: Adjusted HRs for incidence of specific types of musculoskeletal diseases associated with ever-regular smoking in men and women combined


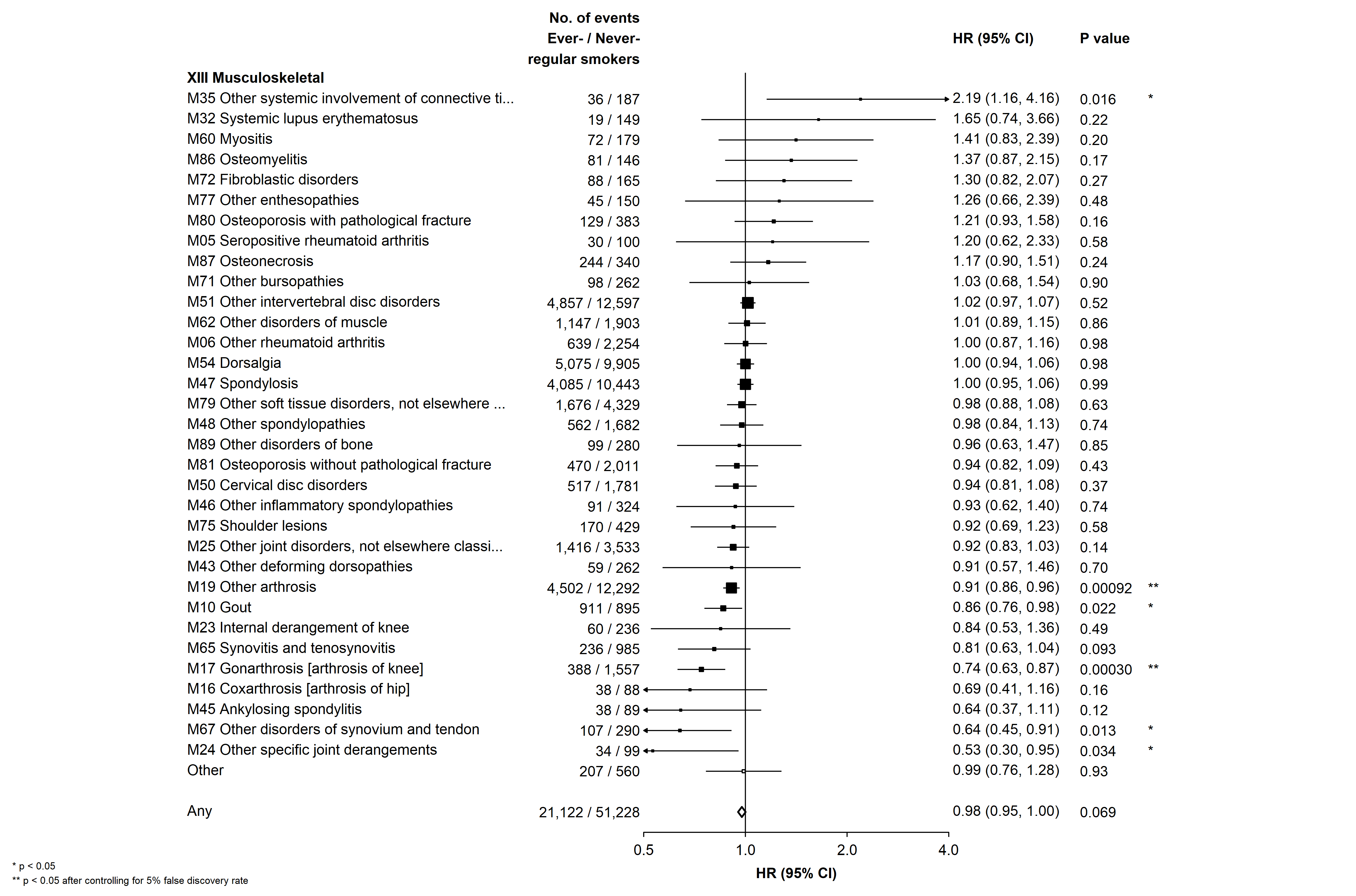


# eFigure 16: Adjusted HRs for incidence of specific types of genitourinary diseases associated with ever-regular smoking in men and women combined


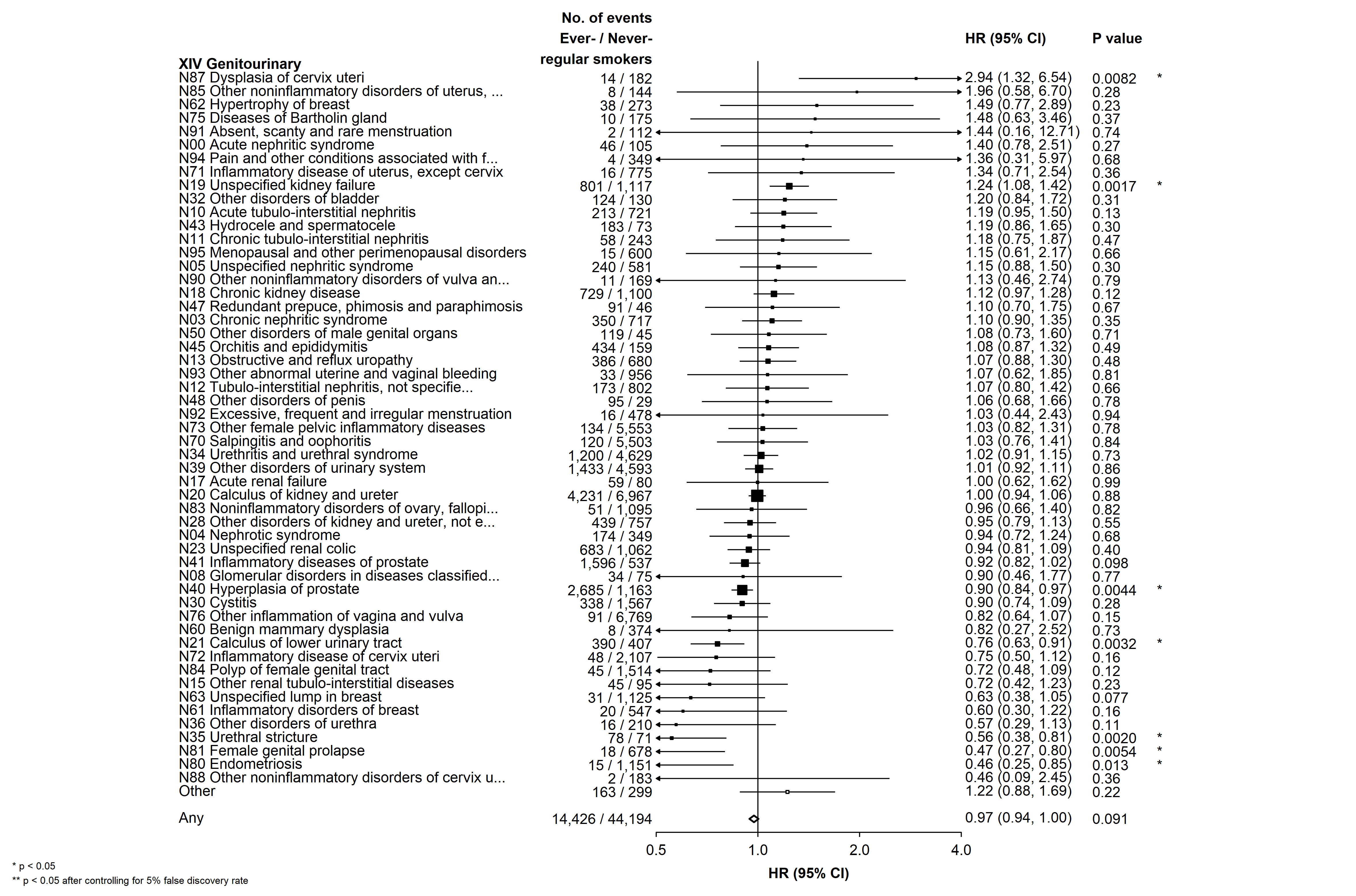


# eFigure 17: Adjusted HRs for incidence of specific types of pregnancy-related diseases associated with ever-regular smoking in men and women combined


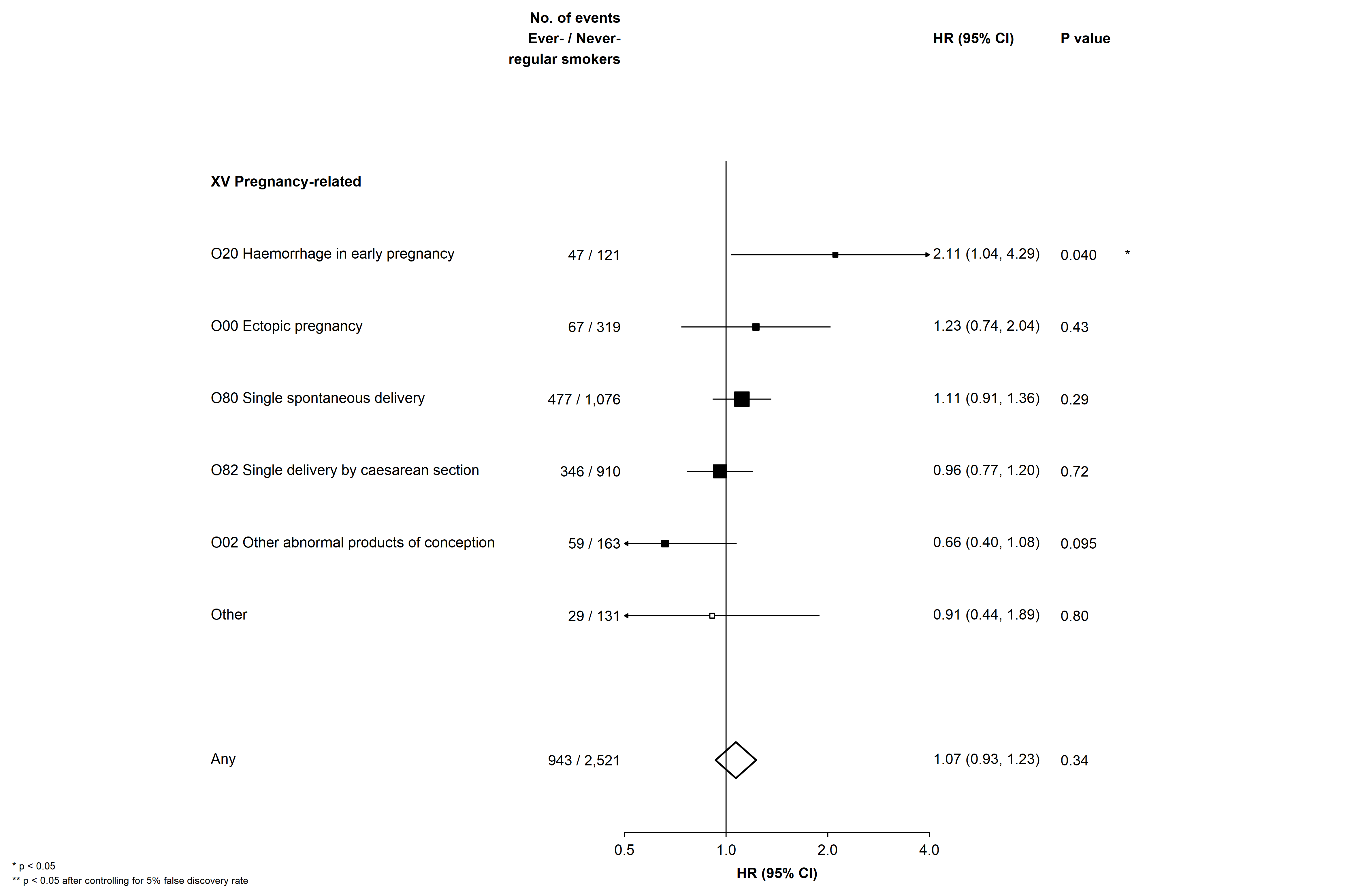


# eFigure 18: Adjusted HRs for incidence of other symptoms, signs and abnormal findings associated with ever-regular smoking in men and women combined


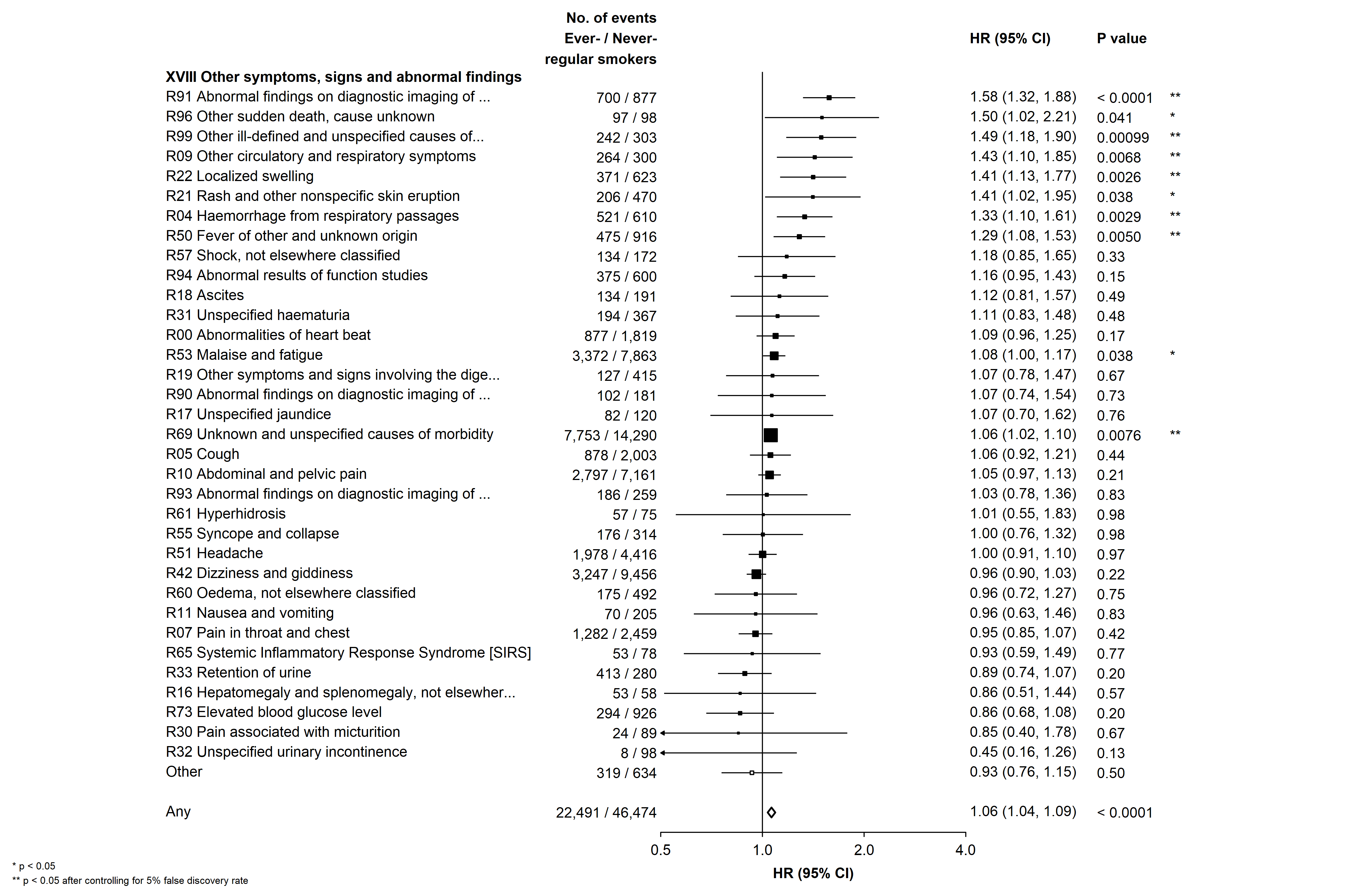


# eFigure 19: Adjusted HRs for incidence of specific types of injury, poisoning and other external causes associated with ever-regular smoking in men and women combined


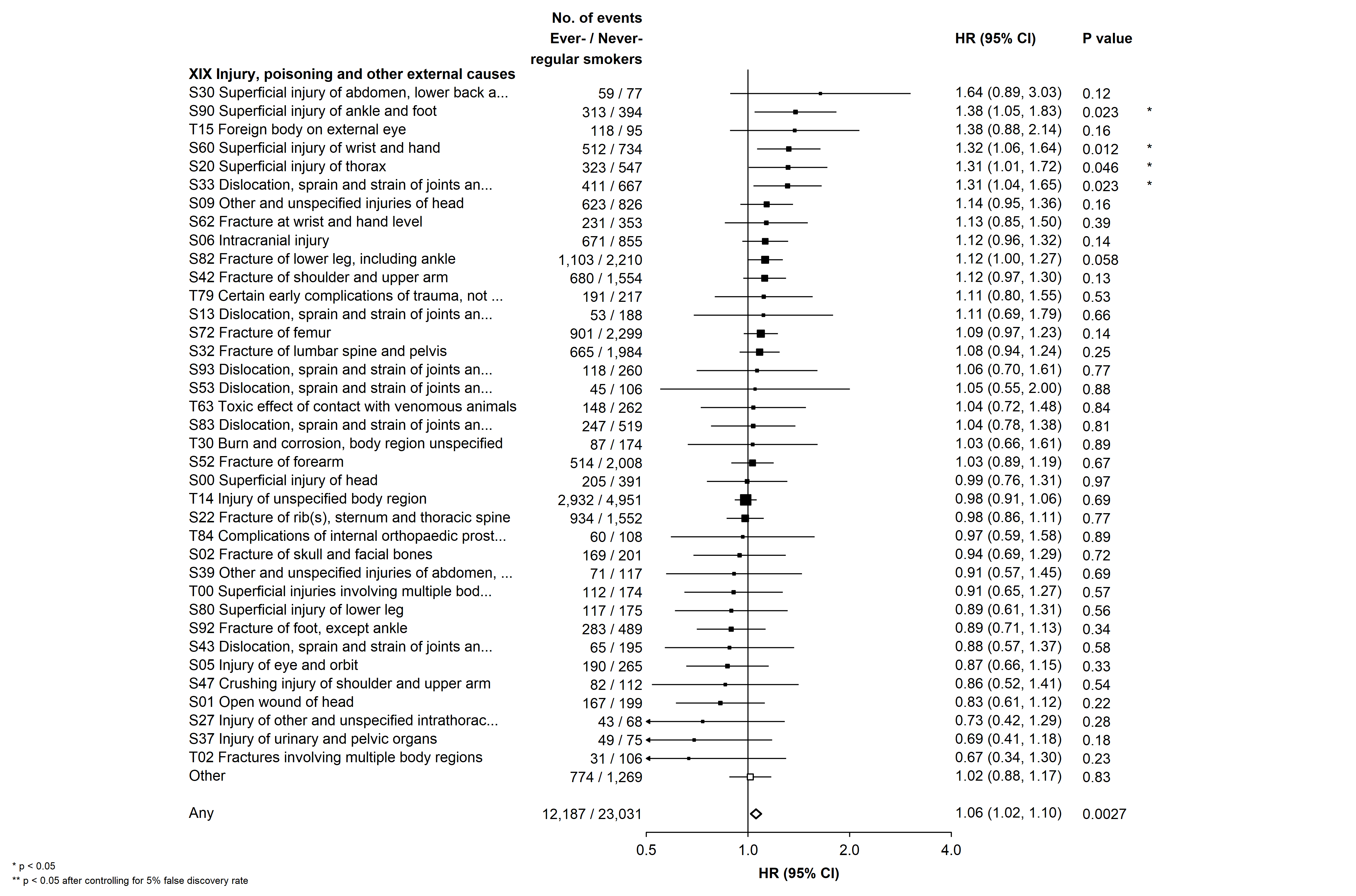


# eFigure 20: Adjusted HRs for incidence of specific external causes associated with ever-regular smoking in men and women combined


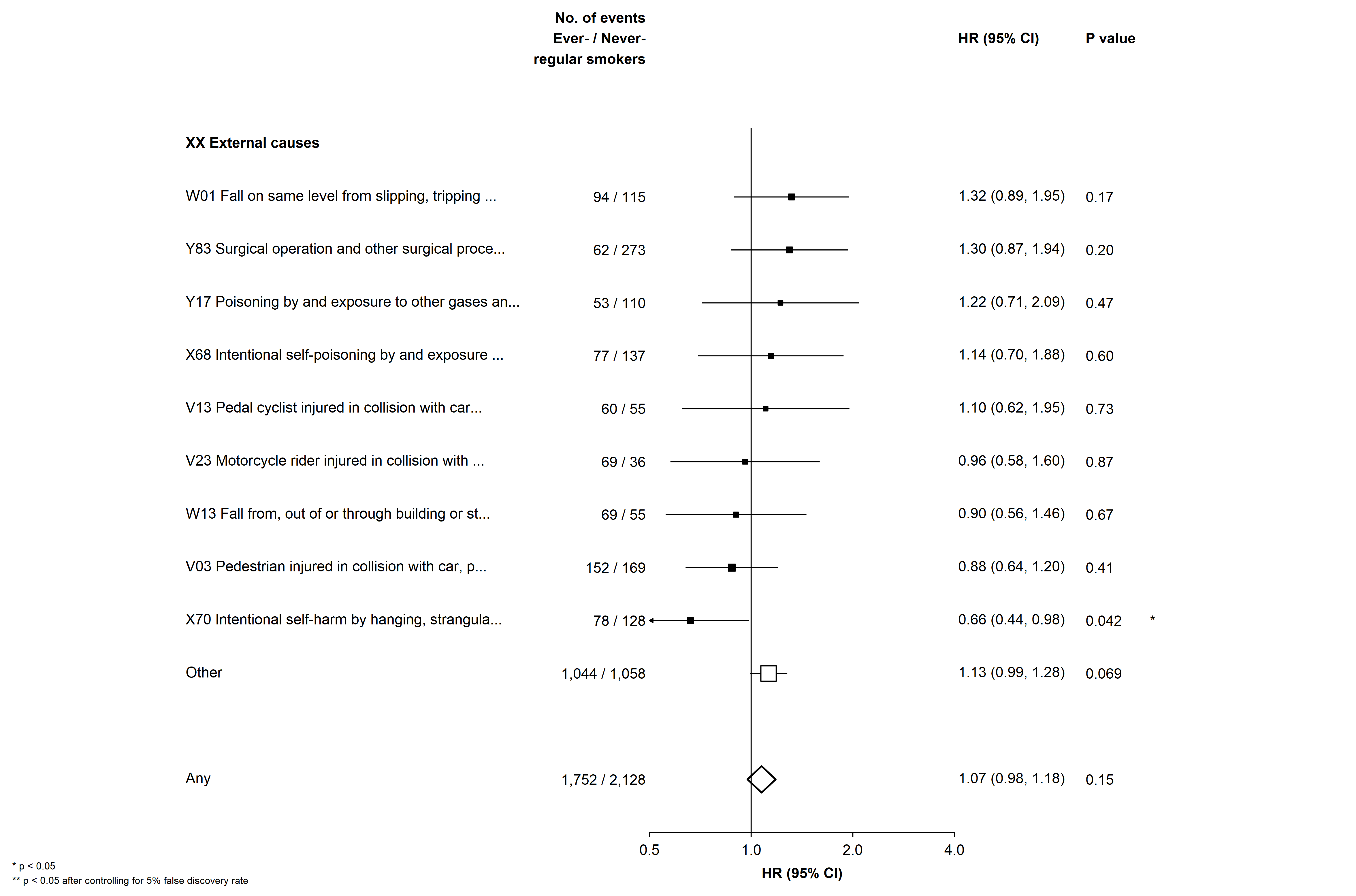


# eFigure 21: Adjusted HRs for cause-specific mortality significantly associated with ever-regular smoking


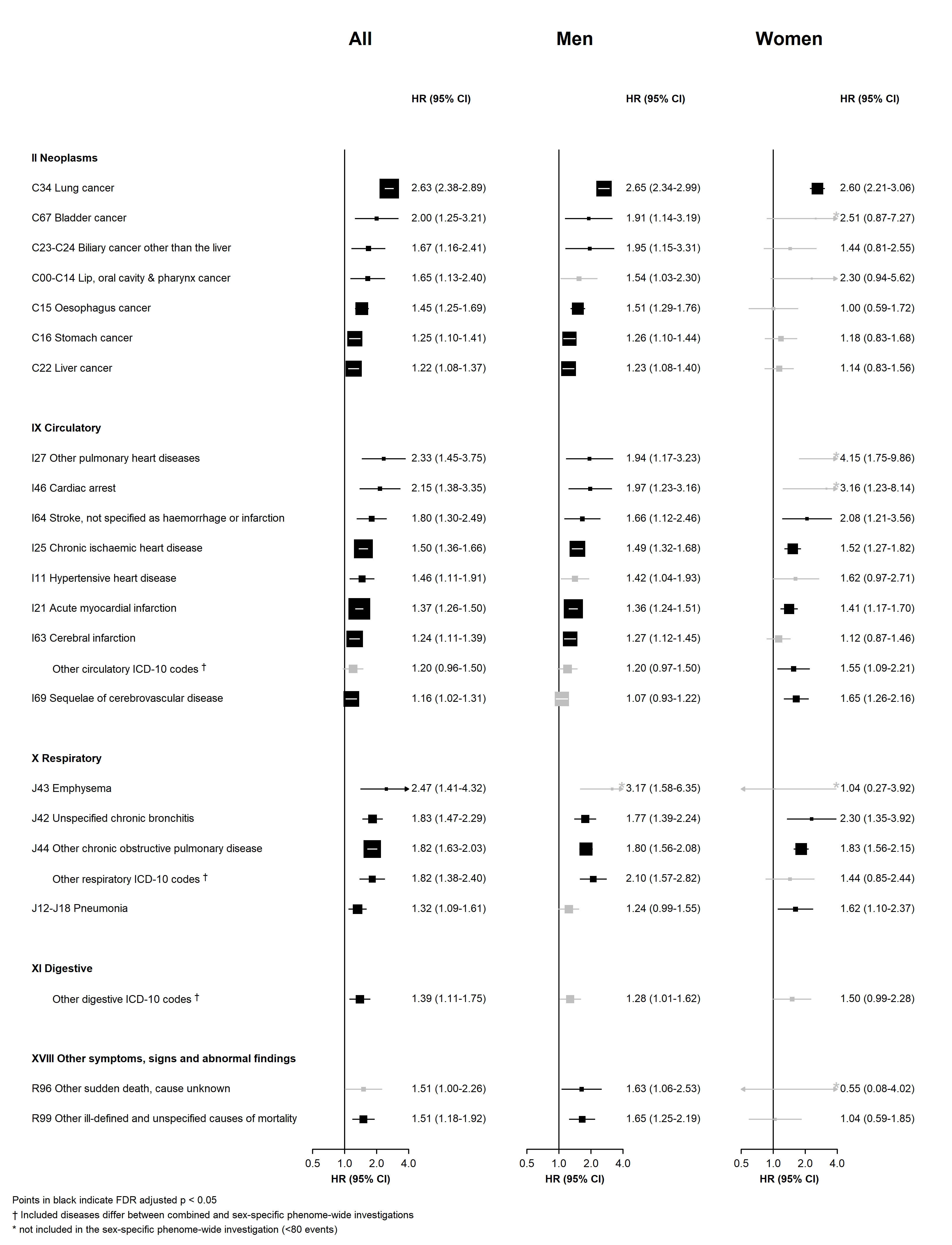


# eTable 6: Comparison of FDR-adjusted significant positive associations with ever-regular smoking between morbidity and mortality analyses, overall and by sex

|  | **Incidence** | | | **Mortality** | | |
| --- | --- | --- | --- | --- | --- | --- |
|  | **All** | **Men** | **Women** | **All** | **Men** | **Women** |
| **I Infectious and parasitic** |  |  |  |  |  |  |
| A16 Respiratory tuberculosis | Yes | Yes |  |  |  |  |
| A17-A19 Extra-pulmonary TB |  | Yes |  |  |  |  |
| **II Neoplasms** |  |  |  |  |  |  |
| C00-C14 Lip, oral cavity & pharynx cancer |  |  |  | Yes |  |  |
| C15 Oesophagus cancer | Yes | Yes |  | Yes | Yes |  |
| C16 Stomach cancer | Yes | Yes |  | Yes | Yes |  |
| C22 Liver cancer | Yes | Yes |  | Yes | Yes |  |
| C23-C24 Biliary cancer other than the liver |  | Yes |  | Yes | Yes |  |
| C32 Larynx cancer | Yes | Yes |  |  |  |  |
| C34 Lung cancer | Yes | Yes | Yes | Yes | Yes | Yes |
| C67 Bladder cancer | Yes | Yes |  | Yes | Yes |  |
| C78 Secondary cancer respiratory and digestive organs | Yes | Yes |  |  |  |  |
| C79 Secondary malignant neoplasm of other and unspecified... | Yes | Yes |  |  |  |  |
| C80 Other cancer | Yes | Yes |  |  |  |  |
| D02 Carcinoma in situ of middle ear and respiratory system | Yes | Yes |  |  |  |  |
| D11 Benign neoplasm (salivary glands) | Yes | Yes |  |  |  |  |
| D36 Benign neoplasm (other) | Yes |  |  |  |  |  |
| D38 Uncertain neoplasm (middle ear/respiratory/intrathoracic) | Yes | Yes |  |  |  |  |
| D41 Uncertain neoplasm (urinary organs) | Yes | Yes |  |  |  |  |
| **IV Endocrine, nutritional and metabolic** |  |  |  |  |  |  |
| E10-E14 Diabetes mellitus | Yes |  | Yes |  |  |  |
| E16 Other disorders of pancreatic internal secretion |  |  | Yes |  |  |  |
| E87 Other disorders of fluid, electrolyte and acid-base b... | Yes |  | Yes |  |  |  |
| E88 Other metabolic disorders | Yes |  | Yes |  |  |  |
| **IX Circulatory** |  |  |  |  |  |  |
| I11 Hypertensive heart disease |  |  |  | Yes |  |  |
| I20 Angina pectoris |  | Yes |  |  |  |  |
| I21 Acute myocardial infarction | Yes | Yes | Yes | Yes | Yes | Yes |
| I25 Chronic ischaemic heart disease | Yes | Yes | Yes | Yes | Yes | Yes |
| I26 Pulmonary embolism |  | Yes |  |  |  |  |
| I27 Other pulmonary heart diseases | Yes | Yes | Yes | Yes | Yes |  |
| I46 Cardiac arrest | Yes | Yes |  | Yes | Yes |  |
| I50 Heart failure | Yes | Yes | Yes |  |  |  |
| I51 Complications of heart disease | Yes | Yes |  |  |  |  |
| I63 Cerebral infarction | Yes | Yes | Yes | Yes | Yes |  |
| I64 Stroke, not specified as haemorrhage or infarction |  |  |  | Yes | Yes | Yes |
| I69 Sequelae of cerebrovascular disease |  |  | Yes | Yes |  | Yes |
| I70 Atherosclerosis | Yes | Yes |  |  |  |  |
| I71 Aortic aneurysm and dissection | Yes | Yes |  |  |  |  |
| I74 Arterial embolism and thrombosis | Yes | Yes |  |  |  |  |
| Other circulatory ICD-10 codes |  |  |  |  |  | Yes |
| **VII Eye and adnexa** |  |  |  |  |  |  |
| H25, H26.9 Cataract | Yes | Yes |  |  |  |  |
| H26 Other cataract | Yes | Yes |  |  |  |  |
| **X Respiratory** |  |  |  |  |  |  |
| J00 Acute nasopharyngitis [common cold] | Yes | Yes | Yes |  |  |  |
| J12-J18 Pneumonia | Yes | Yes | Yes | Yes |  | Yes |
| J40 Bronchitis, not specified as acute or chronic | Yes | Yes |  |  |  |  |
| J41 Simple and mucopurulent chronic bronchitis | Yes | Yes | Yes |  |  |  |
| J42 Unspecified chronic bronchitis | Yes | Yes | Yes | Yes | Yes | Yes |
| J43 Emphysema | Yes | Yes | Yes | Yes |  |  |
| J44 Other chronic obstructive pulmonary disease | Yes | Yes | Yes | Yes | Yes | Yes |
| J45 Asthma | Yes | Yes | Yes |  |  |  |
| J85 Abscess of lung and mediastinum | Yes | Yes |  |  |  |  |
| J90-J91 Pleural effusion | Yes |  | Yes |  |  |  |
| J93 Pneumothorax | Yes | Yes | Yes |  |  |  |
| J96 Respiratory failure, not elsewhere classified | Yes | Yes | Yes |  |  |  |
| J98 Other respiratory disorders | Yes | Yes |  |  |  |  |
| Other respiratory ICD-10 codes | Yes | Yes | Yes | Yes | Yes |  |
| **XI Digestive** |  |  |  |  |  |  |
| K25 Gastric ulcer | Yes |  |  |  |  |  |
| K26 Duodenal ulcer | Yes |  |  |  |  |  |
| K27 Peptic ulcer, site unspecified | Yes | Yes |  |  |  |  |
| K70 Alcoholic liver disease | Yes | Yes |  |  |  |  |
| K74 Fibrosis and cirrhosis of liver |  |  | Yes |  |  |  |
| K92 Other diseases of digestive system | Yes |  |  |  |  |  |
| Other digestive ICD-10 codes |  |  |  | Yes |  |  |
| **XIV Genitourinary** |  |  |  |  |  |  |
| N19 Unspecified kidney failure |  | Yes |  |  |  |  |
| **XVIII Other symptoms, signs and abnormal findings** |  |  |  |  |  |  |
| R04 Haemorrhage from respiratory passages | Yes |  |  |  |  |  |
| R09 Other circulatory and respiratory symptoms | Yes |  |  |  |  |  |
| R22 Localized swelling | Yes | Yes |  |  |  |  |
| R50 Fever of other and unknown origin | Yes | Yes |  |  |  |  |
| R69 Unknown and unspecified causes of morbidity | Yes |  |  |  |  |  |
| R91 Abnormal findings on diagnostic imaging of lung | Yes | Yes | Yes |  |  |  |
| R96 Other sudden death, cause unknown |  |  |  |  | Yes |  |
| R99 Other ill-defined and unspecified causes of mortality | Yes | Yes |  | Yes | Yes |  |

# eFigure 22: Adjusted HRs for cause-specific incidence significantly associated with ever-regular smoking after further adjustment


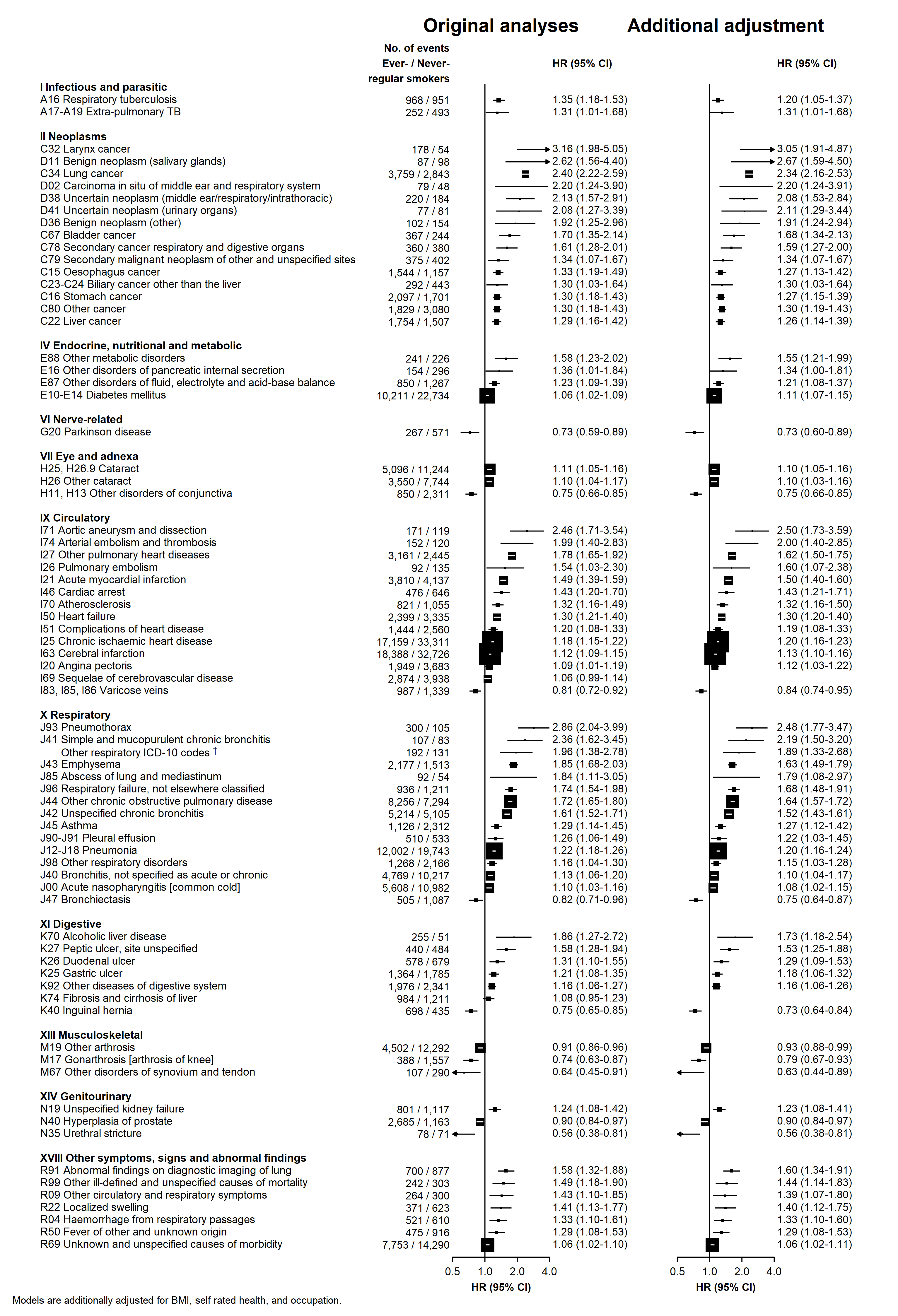


# eTable 7: Adjusted HRs for incident risks of five major diseases associated with smoking, among men and women

|  | **Lung cancer** | | **Ischaemic heart disease** | | **Ischaemic stroke** | | **Haemorrhagic stroke** | | **COPD** | |
| --- | --- | --- | --- | --- | --- | --- | --- | --- | --- | --- |
| **Smoking category** | **Number of events** | **HR (95% CI)^†^** | **Number of events** | **HR (95% CI)^†^** | **Number of events** | **HR (95% CI)^†^** | **Number of events** | **HR (95% CI)^†^** | **Number of events** | **HR (95% CI)^†^** |
| **Men** |  |  |  |  |  |  |  |  |  |  |
| Never-regular smoker | 500 | 1.00 (0.91, 1.09) | 5,545 | 1.00 (0.97, 1.03) | 6,205 | 1.00 (0.97, 1.03) | 1,510 | 1.00 (0.95, 1.05) | 1,884 | 1.00 (0.96, 1.05) |
| Ever-regular smoker | 3,442 | 2.40 (2.18, 2.64) | 16,888 | 1.23 (1.19, 1.27) | 16,715 | 1.12 (1.09, 1.16) | 4,645 | 1.03 (0.97, 1.09) | 9,375 | 1.67 (1.59, 1.76) |
| Ex-smoker (by choice)^‡^ | 271 | 1.52 (1.35, 1.71) | 1,862 | 1.10 (1.05, 1.15) | 1,939 | 1.00 (0.96, 1.05) | 423 | 0.97 (0.88, 1.07) | 675 | 1.20 (1.11, 1.29) |
| Regular smoker | 3,171 | 2.55 (2.45, 2.65) | 15,026 | 1.25 (1.23, 1.28) | 14,776 | 1.14 (1.12, 1.16) | 4,222 | 1.04 (1.00, 1.07) | 8,700 | 1.74 (1.70, 1.78) |
| Ex-smoker (ill health) | 409 | 2.15 (1.95, 2.37) | 2,475 | 1.52 (1.46, 1.59) | 2,322 | 1.23 (1.18, 1.28) | 612 | 1.24 (1.14, 1.34) | 1,633 | 2.45 (2.33, 2.57) |
| Current smoker | 2,762 | 2.64 (2.53, 2.75) | 12,551 | 1.20 (1.18, 1.23) | 12,454 | 1.13 (1.10, 1.15) | 3,610 | 1.00 (0.97, 1.04) | 7,067 | 1.61 (1.57, 1.65) |
| Age began smoking (years)^*^ |  |  |  |  |  |  |  |  |  |  |
| 25+ | 832 | 1.94 (1.81, 2.07) | 4,778 | 1.16 (1.12, 1.19) | 4,822 | 1.08 (1.05, 1.11) | 1,429 | 0.99 (0.94, 1.04) | 2,362 | 1.37 (1.32, 1.43) |
| 18-24 | 1,691 | 2.74 (2.61, 2.87) | 7,620 | 1.27 (1.24, 1.30) | 7,497 | 1.16 (1.13, 1.18) | 2,051 | 1.04 (0.99, 1.09) | 4,413 | 1.85 (1.80, 1.91) |
| <18 | 648 | 3.41 (3.15, 3.69) | 2,628 | 1.45 (1.39, 1.51) | 2,457 | 1.26 (1.21, 1.31) | 742 | 1.12 (1.04, 1.21) | 1,925 | 2.24 (2.14, 2.35) |
| No. smoked (cig/day)^*^ |  |  |  |  |  |  |  |  |  |  |
| <15 | 859 | 1.91 (1.78, 2.04) | 5,739 | 1.19 (1.15, 1.22) | 5,868 | 1.09 (1.06, 1.12) | 1,736 | 1.07 (1.02, 1.12) | 2,712 | 1.58 (1.52, 1.64) |
| 15-24 | 1,519 | 2.69 (2.56, 2.83) | 6,520 | 1.26 (1.23, 1.29) | 6,361 | 1.16 (1.13, 1.19) | 1,729 | 0.98 (0.94, 1.03) | 3,943 | 1.80 (1.74, 1.85) |
| 25+ | 793 | 3.63 (3.37, 3.90) | 2,767 | 1.45 (1.40, 1.51) | 2,547 | 1.25 (1.20, 1.30) | 757 | 1.07 (0.99, 1.15) | 2,045 | 1.90 (1.82, 1.99) |
| **Women** |  |  |  |  |  |  |  |  |  |  |
| Never-regular smoker | 2,346 | 1.00 (0.93, 1.08) | 30,107 | 1.00 (0.98, 1.02) | 26,521 | 1.00 (0.98, 1.02) | 5,141 | 1.00 (0.94, 1.06) | 8,687 | 1.00 (0.96, 1.04) |
| Ever-regular smoker | 318 | 2.43 (2.12, 2.78) | 2,082 | 1.11 (1.06, 1.16) | 1,673 | 1.10 (1.05, 1.16) | 335 | 1.12 (0.99, 1.27) | 1,557 | 1.68 (1.58, 1.78) |
| Ex-smoker (by choice)^‡^ | 37 | 1.85 (1.34, 2.56) | 301 | 1.03 (0.92, 1.15) | 256 | 1.02 (0.90, 1.15) | 48 | 1.25 (0.94, 1.66) | 137 | 1.23 (1.04, 1.45) |
| Regular smoker | 281 | 2.53 (2.25, 2.84) | 1,781 | 1.12 (1.07, 1.18) | 1,417 | 1.12 (1.06, 1.18) | 287 | 1.11 (0.99, 1.24) | 1,420 | 1.74 (1.65, 1.83) |
| Ex-smoker (ill health) | 49 | 2.31 (1.74, 3.07) | 371 | 1.32 (1.19, 1.46) | 279 | 1.26 (1.12, 1.42) | 63 | 1.21 (0.95, 1.56) | 384 | 2.24 (2.02, 2.48) |
| Current smoker | 232 | 2.57 (2.27, 2.92) | 1,410 | 1.09 (1.03, 1.14) | 1,138 | 1.09 (1.03, 1.16) | 224 | 1.08 (0.95, 1.23) | 1,036 | 1.61 (1.52, 1.71) |
| Age began smoking (years)^*^ |  |  |  |  |  |  |  |  |  |  |
| 25+ | 109 | 1.92 (1.60, 2.31) | 937 | 1.11 (1.05, 1.19) | 758 | 1.10 (1.03, 1.18) | 147 | 1.13 (0.97, 1.32) | 548 | 1.56 (1.43, 1.69) |
| 18-24 | 90 | 2.90 (2.35, 3.57) | 493 | 1.13 (1.03, 1.23) | 390 | 1.15 (1.04, 1.27) | 79 | 1.05 (0.84, 1.31) | 480 | 1.82 (1.66, 1.99) |
| <18 | 82 | 3.77 (3.02, 4.70) | 351 | 1.16 (1.04, 1.28) | 269 | 1.13 (1.00, 1.28) | 61 | 1.08 (0.84, 1.40) | 392 | 1.99 (1.80, 2.20) |
| No. smoked (cig/day)^*^ |  |  |  |  |  |  |  |  |  |  |
| <15 | 213 | 2.47 (2.16, 2.82) | 1,365 | 1.11 (1.05, 1.17) | 1,081 | 1.10 (1.03, 1.16) | 209 | 1.04 (0.91, 1.19) | 1,049 | 1.68 (1.58, 1.78) |
| 15+ | 68 | 2.73 (2.15, 3.47) | 416 | 1.17 (1.06, 1.28) | 336 | 1.20 (1.08, 1.34) | 78 | 1.29 (1.03, 1.61) | 371 | 1.94 (1.75, 2.15) |
| ^†^ All analyses were stratified by study area and 5-year age-at-risk group and adjusted for education and alcohol drinking. ^‡^ Only include those who had quit voluntarily (i.e. not due to ill health). ^*^ Include current smokers and ex-smokers who had quit due to ill health. | | | | | | | | | | |

# eTable 8: Adjusted HRs for mortality risks of five major diseases associated with smoking, among urban and rural men

|  | **Lung cancer mortality** | | **Ischaemic heart disease mortality** | | **Ischaemic stroke mortality** | | **Haemorrhagic stroke mortality** | | **COPD mortality** | |
| --- | --- | --- | --- | --- | --- | --- | --- | --- | --- | --- |
| **Smoking category** | **Number of events** | **HR (95% CI)^†^** | **Number of events** | **HR (95% CI)^†^** | **Number of events** | **HR (95% CI)^†^** | **Number of events** | **HR (95% CI)^†^** | **Number of events** | **HR (95% CI)^†^** |
| **Urban men** |  |  |  |  |  |  |  |  |  |  |
| Never-regular smoker | 138 | 1.00 (0.84, 1.19) | 456 | 1.00 (0.91, 1.10) | 158 | 1.00 (0.85, 1.18) | 160 | 1.00 (0.85, 1.18) | 49 | 1.00 (0.75, 1.33) |
| Ever-regular smoker | 825 | 3.07 (2.55, 3.70) | 1,164 | 1.49 (1.33, 1.66) | 367 | 1.40 (1.15, 1.70) | 341 | 1.24 (1.02, 1.51) | 240 | 3.57 (2.60, 4.89) |
| Ex-smoker (by choice)^‡^ | 80 | 1.48 (1.19, 1.84) | 177 | 1.08 (0.93, 1.25) | 50 | 0.90 (0.68, 1.19) | 53 | 1.06 (0.81, 1.40) | 22 | 1.42 (0.93, 2.15) |
| Regular smoker | 745 | 3.53 (3.27, 3.81) | 987 | 1.61 (1.50, 1.72) | 317 | 1.55 (1.38, 1.74) | 288 | 1.29 (1.14, 1.45) | 218 | 4.26 (3.71, 4.89) |
| Ex-smoker (ill health) | 131 | 2.70 (2.27, 3.20) | 253 | 1.75 (1.55, 1.99) | 88 | 1.74 (1.41, 2.15) | 70 | 1.58 (1.24, 2.00) | 87 | 5.95 (4.82, 7.36) |
| Current smoker | 614 | 3.82 (3.50, 4.17) | 734 | 1.55 (1.44, 1.68) | 229 | 1.48 (1.29, 1.69) | 218 | 1.20 (1.04, 1.39) | 131 | 3.55 (2.97, 4.24) |
| Age began smoking (years)^*^ |  |  |  |  |  |  |  |  |  |  |
| 25+ | 203 | 2.59 (2.25, 2.98) | 327 | 1.42 (1.27, 1.58) | 117 | 1.45 (1.21, 1.74) | 94 | 1.17 (0.95, 1.43) | 70 | 3.25 (2.57, 4.12) |
| 18-24 | 410 | 3.99 (3.61, 4.40) | 501 | 1.70 (1.56, 1.87) | 138 | 1.41 (1.19, 1.67) | 147 | 1.30 (1.10, 1.54) | 100 | 4.56 (3.74, 5.56) |
| <18 | 132 | 4.61 (3.87, 5.49) | 159 | 2.00 (1.70, 2.34) | 62 | 2.23 (1.73, 2.88) | 47 | 1.44 (1.08, 1.93) | 48 | 7.60 (5.67, 10.19) |
| No. smoked (cig/day)^*^ |  |  |  |  |  |  |  |  |  |  |
| <15 | 247 | 2.77 (2.44, 3.14) | 353 | 1.36 (1.22, 1.51) | 130 | 1.52 (1.28, 1.81) | 108 | 1.22 (1.01, 1.48) | 65 | 2.93 (2.29, 3.76) |
| 15-24 | 344 | 3.71 (3.34, 4.13) | 449 | 1.71 (1.56, 1.88) | 136 | 1.50 (1.27, 1.78) | 130 | 1.26 (1.06, 1.49) | 108 | 5.05 (4.18, 6.09) |
| 25+ | 154 | 5.01 (4.27, 5.88) | 185 | 2.17 (1.88, 2.51) | 51 | 1.61 (1.22, 2.13) | 50 | 1.39 (1.05, 1.84) | 45 | 6.33 (4.71, 8.50) |
| **Rural men** |  |  |  |  |  |  |  |  |  |  |
| Never-regular smoker | 168 | 1.00 (0.86, 1.17) | 479 | 1.00 (0.91, 1.10) | 175 | 1.00 (0.86, 1.16) | 581 | 1.00 (0.92, 1.09) | 277 | 1.00 (0.89, 1.13) |
| Ever-regular smoker | 1,484 | 2.32 (1.98, 2.73) | 1,949 | 1.36 (1.23, 1.51) | 594 | 1.17 (0.99, 1.39) | 2,109 | 1.03 (0.94, 1.13) | 1,564 | 1.60 (1.40, 1.82) |
| Ex-smoker (by choice)^‡^ | 82 | 1.44 (1.16, 1.79) | 136 | 1.17 (0.99, 1.39) | 50 | 1.03 (0.78, 1.35) | 142 | 0.94 (0.80, 1.11) | 108 | 1.33 (1.10, 1.61) |
| Regular smoker | 1,402 | 2.41 (2.29, 2.55) | 1,813 | 1.38 (1.31, 1.45) | 544 | 1.19 (1.09, 1.30) | 1,967 | 1.03 (0.99, 1.08) | 1,456 | 1.62 (1.54, 1.71) |
| Ex-smoker (ill health) | 150 | 2.01 (1.71, 2.37) | 266 | 1.77 (1.57, 2.00) | 109 | 1.64 (1.36, 1.98) | 261 | 1.27 (1.12, 1.43) | 352 | 2.91 (2.61, 3.23) |
| Current smoker | 1,252 | 2.47 (2.33, 2.63) | 1,547 | 1.32 (1.25, 1.39) | 435 | 1.10 (1.00, 1.22) | 1,706 | 1.00 (0.96, 1.06) | 1,104 | 1.41 (1.33, 1.50) |
| Age began smoking (years)^*^ |  |  |  |  |  |  |  |  |  |  |
| 25+ | 358 | 1.78 (1.60, 1.98) | 602 | 1.18 (1.09, 1.29) | 188 | 1.03 (0.89, 1.19) | 715 | 0.98 (0.91, 1.06) | 427 | 1.23 (1.12, 1.36) |
| 18-24 | 730 | 2.55 (2.37, 2.74) | 881 | 1.47 (1.37, 1.57) | 263 | 1.26 (1.12, 1.42) | 885 | 1.02 (0.95, 1.09) | 658 | 1.66 (1.53, 1.79) |
| <18 | 314 | 3.34 (2.99, 3.74) | 330 | 1.60 (1.43, 1.79) | 93 | 1.33 (1.08, 1.63) | 367 | 1.19 (1.07, 1.32) | 371 | 2.46 (2.22, 2.73) |
| No. smoked (cig/day)^*^ |  |  |  |  |  |  |  |  |  |  |
| <15 | 362 | 1.78 (1.60, 1.98) | 851 | 1.36 (1.27, 1.46) | 282 | 1.25 (1.10, 1.41) | 908 | 1.13 (1.06, 1.21) | 616 | 1.73 (1.59, 1.88) |
| 15-24 | 653 | 2.49 (2.31, 2.69) | 701 | 1.38 (1.28, 1.49) | 183 | 1.03 (0.89, 1.20) | 723 | 0.93 (0.86, 1.00) | 577 | 1.57 (1.45, 1.71) |
| 25+ | 387 | 3.59 (3.23, 3.99) | 261 | 1.42 (1.25, 1.61) | 79 | 1.29 (1.02, 1.63) | 336 | 1.02 (0.91, 1.14) | 263 | 1.47 (1.29, 1.68) |
| ^†^ All analyses were stratified by study area and 5-year age-at-risk group and adjusted for education and alcohol drinking. ^‡^ Only include those who had quit voluntarily (i.e. not due to ill health). ^*^ Include current smokers and ex-smokers who had quit due to ill health. | | | | | | | | | | |

# eTable 9: Adjusted HRs for incident risks of five major diseases associated with smoking after exclusion of relevant prior disease reported at baseline, among men and women

|  | **Lung cancer** | | **Ischaemic heart disease** | | **Ischaemic stroke** | | **Haemorrhagic stroke** | | **COPD** | |
| --- | --- | --- | --- | --- | --- | --- | --- | --- | --- | --- |
| **Smoking category** | **Number of events** | **HR (95% CI)^†^** | **Number of events** | **HR (95% CI)^†^** | **Number of events** | **HR (95% CI)^†^** | **Number of events** | **HR (95% CI)^†^** | **Number of events** | **HR (95% CI)^†^** |
| **Men** |  |  |  |  |  |  |  |  |  |  |
| Never-regular smoker | 488 | 1.00 (0.91, 1.09) | 4,490 | 1.00 (0.97, 1.03) | 5,143 | 1.00 (0.97, 1.03) | 1,263 | 1.00 (0.95, 1.06) | 1,047 | 1.00 (0.94, 1.06) |
| Ever-regular smoker | 3,365 | 2.39 (2.17, 2.64) | 14,126 | 1.24 (1.20, 1.28) | 14,164 | 1.13 (1.09, 1.17) | 3,994 | 1.04 (0.97, 1.11) | 5,066 | 1.67 (1.56, 1.79) |
| Ex-smoker (by choice)^‡^ | 260 | 1.49 (1.31, 1.68) | 1,482 | 1.10 (1.05, 1.16) | 1,624 | 1.04 (0.99, 1.09) | 351 | 0.97 (0.88, 1.08) | 368 | 1.21 (1.09, 1.34) |
| Regular smoker | 3,105 | 2.55 (2.45, 2.65) | 12,644 | 1.26 (1.24, 1.28) | 12,540 | 1.14 (1.12, 1.17) | 3,643 | 1.04 (1.01, 1.08) | 4,698 | 1.73 (1.68, 1.79) |
| Ex-smoker (ill health) | 364 | 1.98 (1.78, 2.19) | 1,648 | 1.41 (1.34, 1.48) | 1,550 | 1.13 (1.07, 1.19) | 423 | 1.12 (1.02, 1.23) | 568 | 1.82 (1.68, 1.98) |
| Current smoker | 2,741 | 2.67 (2.56, 2.79) | 10,996 | 1.24 (1.21, 1.26) | 10,990 | 1.15 (1.12, 1.17) | 3,220 | 1.03 (1.00, 1.07) | 4,130 | 1.72 (1.66, 1.78) |
| Age began smoking (years)^*^ |  |  |  |  |  |  |  |  |  |  |
| 25+ | 821 | 1.95 (1.82, 2.09) | 3,999 | 1.17 (1.13, 1.20) | 4,125 | 1.09 (1.06, 1.13) | 1,257 | 1.01 (0.96, 1.07) | 1,385 | 1.46 (1.39, 1.54) |
| 18-24 | 1,649 | 2.72 (2.59, 2.86) | 6,404 | 1.27 (1.24, 1.30) | 6,353 | 1.15 (1.12, 1.18) | 1,748 | 1.04 (0.99, 1.09) | 2,377 | 1.82 (1.75, 1.89) |
| <18 | 635 | 3.41 (3.15, 3.69) | 2,241 | 1.46 (1.40, 1.52) | 2,062 | 1.24 (1.18, 1.29) | 638 | 1.12 (1.04, 1.21) | 936 | 2.12 (1.99, 2.26) |
| No. smoked (cig/day)^*^ |  |  |  |  |  |  |  |  |  |  |
| <15 | 847 | 1.92 (1.79, 2.05) | 4,740 | 1.19 (1.15, 1.22) | 4,844 | 1.08 (1.05, 1.11) | 1,462 | 1.06 (1.01, 1.12) | 1,436 | 1.56 (1.48, 1.64) |
| 15-24 | 1,480 | 2.68 (2.55, 2.82) | 5,537 | 1.26 (1.23, 1.30) | 5,495 | 1.17 (1.14, 1.20) | 1,517 | 1.01 (0.96, 1.06) | 2,170 | 1.78 (1.71, 1.86) |
| 25+ | 778 | 3.63 (3.38, 3.91) | 2,367 | 1.45 (1.39, 1.52) | 2,201 | 1.26 (1.20, 1.31) | 664 | 1.08 (1.00, 1.17) | 1,092 | 1.94 (1.82, 2.06) |
| **Women** |  |  |  |  |  |  |  |  |  |  |
| Never-regular smoker | 2,301 | 1.00 (0.92, 1.08) | 25,219 | 1.00 (0.98, 1.03) | 22,717 | 1.00 (0.97, 1.03) | 4,509 | 1.00 (0.93, 1.07) | 5,586 | 1.00 (0.95, 1.05) |
| Ever-regular smoker | 311 | 2.39 (2.08, 2.74) | 1,665 | 1.13 (1.07, 1.20) | 1,299 | 1.11 (1.04, 1.17) | 282 | 1.08 (0.94, 1.24) | 769 | 1.64 (1.51, 1.79) |
| Ex-smoker (by choice)^‡^ | 36 | 1.81 (1.30, 2.51) | 239 | 1.13 (0.99, 1.28) | 188 | 1.02 (0.89, 1.18) | 42 | 1.31 (0.96, 1.77) | 65 | 1.11 (0.87, 1.42) |
| Regular smoker | 275 | 2.49 (2.21, 2.80) | 1,426 | 1.13 (1.08, 1.19) | 1,111 | 1.12 (1.06, 1.19) | 240 | 1.05 (0.92, 1.19) | 704 | 1.72 (1.59, 1.85) |
| Ex-smoker (ill health) | 47 | 2.24 (1.67, 2.99) | 274 | 1.31 (1.16, 1.48) | 194 | 1.23 (1.07, 1.42) | 53 | 1.20 (0.91, 1.57) | 132 | 1.74 (1.46, 2.07) |
| Current smoker | 228 | 2.54 (2.24, 2.89) | 1,152 | 1.10 (1.04, 1.17) | 917 | 1.10 (1.03, 1.17) | 187 | 1.02 (0.88, 1.17) | 572 | 1.71 (1.58, 1.85) |
| Age began smoking (years)^*^ |  |  |  |  |  |  |  |  |  |  |
| 25+ | 106 | 1.89 (1.56, 2.27) | 731 | 1.12 (1.05, 1.21) | 583 | 1.11 (1.02, 1.20) | 117 | 1.04 (0.87, 1.24) | 282 | 1.52 (1.36, 1.71) |
| 18-24 | 90 | 2.91 (2.36, 3.59) | 404 | 1.15 (1.04, 1.27) | 315 | 1.16 (1.04, 1.30) | 68 | 1.02 (0.80, 1.29) | 239 | 1.82 (1.60, 2.07) |
| <18 | 79 | 3.64 (2.90, 4.56) | 291 | 1.15 (1.03, 1.29) | 213 | 1.10 (0.96, 1.26) | 55 | 1.07 (0.82, 1.40) | 183 | 1.99 (1.72, 2.30) |
| No. smoked (cig/day)^*^ |  |  |  |  |  |  |  |  |  |  |
| <15 | 211 | 2.46 (2.15, 2.81) | 1,104 | 1.13 (1.07, 1.20) | 856 | 1.10 (1.03, 1.18) | 171 | 0.96 (0.83, 1.12) | 513 | 1.64 (1.51, 1.79) |
| 15+ | 64 | 2.60 (2.03, 3.32) | 322 | 1.14 (1.02, 1.27) | 255 | 1.18 (1.05, 1.34) | 69 | 1.30 (1.03, 1.65) | 191 | 1.97 (1.71, 2.27) |
| ^†^ All analyses were stratified by study area and 5-year age-at-risk group and adjusted for education and alcohol drinking. ^‡^ Only include those who had quit voluntarily (i.e. not due to ill health). ^*^ Include current smokers and ex-smokers who had quit due to ill health. For analyses of ischaemic heart disease and stroke types participants with a prior history of CVD reported at baseline were excluded. Likewise, for analyses of COPD and lung cancer, participants with a prior history of respiratory disease and cancer reported at baseline were excluded respectively. | | | | | | | | | | |

# eTable 10: Adjusted HRs for incident risks of five major diseases associated with smoking after exclusion of relevant prior disease reported at baseline, among urban and rural men

|  | **Lung cancer** | | **Ischaemic heart disease** | | **Ischaemic stroke** | | **Haemorrhagic stroke** | | **COPD** | |
| --- | --- | --- | --- | --- | --- | --- | --- | --- | --- | --- |
| **Smoking category** | **Number of events** | **HR (95% CI)^†^** | **Number of events** | **HR (95% CI)^†^** | **Number of events** | **HR (95% CI)^†^** | **Number of events** | **HR (95% CI)^†^** | **Number of events** | **HR (95% CI)^†^** |
| **Urban men** |  |  |  |  |  |  |  |  |  |  |
| Never-regular smoker | 233 | 1.00 (0.88, 1.14) | 2,362 | 1.00 (0.96, 1.04) | 2,841 | 1.00 (0.96, 1.04) | 363 | 1.00 (0.90, 1.11) | 310 | 1.00 (0.89, 1.12) |
| Ever-regular smoker | 1,196 | 2.61 (2.26, 3.02) | 5,592 | 1.30 (1.23, 1.36) | 5,769 | 1.15 (1.10, 1.21) | 773 | 1.16 (1.02, 1.32) | 897 | 1.96 (1.71, 2.24) |
| Ex-smoker (by choice)^‡^ | 128 | 1.47 (1.23, 1.75) | 865 | 1.13 (1.06, 1.21) | 958 | 1.05 (0.98, 1.12) | 111 | 0.99 (0.82, 1.19) | 123 | 1.31 (1.10, 1.57) |
| Regular smoker | 1,068 | 2.92 (2.74, 3.11) | 4,727 | 1.34 (1.30, 1.38) | 4,811 | 1.18 (1.14, 1.21) | 662 | 1.20 (1.10, 1.30) | 774 | 2.14 (1.98, 2.30) |
| Ex-smoker (ill health) | 174 | 2.25 (1.94, 2.61) | 761 | 1.45 (1.35, 1.56) | 692 | 1.06 (0.98, 1.14) | 99 | 1.16 (0.95, 1.41) | 152 | 1.97 (1.68, 2.32) |
| Current smoker | 894 | 3.13 (2.91, 3.36) | 3,966 | 1.31 (1.27, 1.36) | 4,119 | 1.20 (1.16, 1.24) | 563 | 1.21 (1.10, 1.32) | 622 | 2.18 (2.01, 2.38) |
| Age began smoking (years)^*^ |  |  |  |  |  |  |  |  |  |  |
| 25+ | 301 | 2.27 (2.03, 2.55) | 1,477 | 1.26 (1.19, 1.32) | 1,589 | 1.11 (1.05, 1.16) | 212 | 1.14 (1.00, 1.31) | 271 | 1.83 (1.62, 2.06) |
| 18-24 | 579 | 3.17 (2.91, 3.44) | 2,479 | 1.34 (1.29, 1.40) | 2,506 | 1.19 (1.14, 1.24) | 353 | 1.23 (1.11, 1.37) | 362 | 2.13 (1.92, 2.37) |
| <18 | 188 | 3.70 (3.20, 4.28) | 771 | 1.59 (1.48, 1.71) | 716 | 1.31 (1.21, 1.41) | 97 | 1.16 (0.95, 1.42) | 141 | 3.01 (2.54, 3.57) |
| No. smoked (cig/day)^*^ |  |  |  |  |  |  |  |  |  |  |
| <15 | 329 | 2.20 (1.98, 2.46) | 1,728 | 1.22 (1.16, 1.28) | 1,786 | 1.08 (1.03, 1.13) | 240 | 1.13 (1.00, 1.29) | 273 | 1.90 (1.69, 2.15) |
| 15-24 | 512 | 3.10 (2.85, 3.39) | 2,158 | 1.35 (1.29, 1.41) | 2,224 | 1.21 (1.16, 1.26) | 316 | 1.23 (1.10, 1.37) | 356 | 2.13 (1.92, 2.36) |
| 25+ | 227 | 4.09 (3.59, 4.67) | 841 | 1.68 (1.57, 1.80) | 801 | 1.31 (1.22, 1.41) | 106 | 1.22 (1.01, 1.48) | 145 | 2.50 (2.12, 2.95) |
| **Rural men** |  |  |  |  |  |  |  |  |  |  |
| Never-regular smoker | 255 | 1.00 (0.88, 1.13) | 2,128 | 1.00 (0.96, 1.04) | 2,302 | 1.00 (0.96, 1.04) | 900 | 1.00 (0.94, 1.07) | 737 | 1.00 (0.93, 1.08) |
| Ever-regular smoker | 2,169 | 2.20 (1.93, 2.51) | 8,534 | 1.19 (1.13, 1.25) | 8,395 | 1.11 (1.05, 1.16) | 3,221 | 1.00 (0.93, 1.08) | 4,169 | 1.58 (1.46, 1.71) |
| Ex-smoker (by choice)^‡^ | 132 | 1.51 (1.28, 1.80) | 617 | 1.09 (1.00, 1.18) | 666 | 1.02 (0.95, 1.10) | 240 | 0.98 (0.86, 1.11) | 245 | 1.17 (1.03, 1.33) |
| Regular smoker | 2,037 | 2.27 (2.18, 2.38) | 7,917 | 1.20 (1.17, 1.23) | 7,729 | 1.11 (1.09, 1.14) | 2,981 | 1.00 (0.96, 1.04) | 3,924 | 1.62 (1.56, 1.67) |
| Ex-smoker (ill health) | 190 | 1.74 (1.51, 2.01) | 887 | 1.38 (1.29, 1.47) | 858 | 1.19 (1.11, 1.27) | 324 | 1.10 (0.99, 1.23) | 416 | 1.76 (1.59, 1.93) |
| Current smoker | 1,847 | 2.35 (2.24, 2.47) | 7,030 | 1.18 (1.15, 1.21) | 6,871 | 1.10 (1.08, 1.13) | 2,657 | 0.99 (0.95, 1.03) | 3,508 | 1.60 (1.55, 1.66) |
| Age began smoking (years)^*^ |  |  |  |  |  |  |  |  |  |  |
| 25+ | 520 | 1.70 (1.56, 1.86) | 2,522 | 1.10 (1.06, 1.14) | 2,536 | 1.07 (1.03, 1.12) | 1,045 | 0.97 (0.91, 1.04) | 1,114 | 1.35 (1.27, 1.43) |
| 18-24 | 1,070 | 2.38 (2.25, 2.53) | 3,925 | 1.21 (1.18, 1.25) | 3,847 | 1.12 (1.08, 1.15) | 1,395 | 0.98 (0.93, 1.04) | 2,015 | 1.70 (1.63, 1.78) |
| <18 | 447 | 3.09 (2.81, 3.39) | 1,470 | 1.37 (1.30, 1.44) | 1,346 | 1.19 (1.13, 1.26) | 541 | 1.10 (1.01, 1.19) | 795 | 1.93 (1.79, 2.07) |
| No. smoked (cig/day)^*^ |  |  |  |  |  |  |  |  |  |  |
| <15 | 518 | 1.69 (1.54, 1.85) | 3,012 | 1.16 (1.12, 1.20) | 3,058 | 1.07 (1.03, 1.11) | 1,222 | 1.03 (0.97, 1.09) | 1,163 | 1.45 (1.36, 1.54) |
| 15-24 | 968 | 2.36 (2.21, 2.51) | 3,379 | 1.19 (1.15, 1.24) | 3,271 | 1.13 (1.09, 1.17) | 1,201 | 0.95 (0.89, 1.00) | 1,814 | 1.67 (1.59, 1.75) |
| 25+ | 551 | 3.24 (2.97, 3.54) | 1,526 | 1.31 (1.25, 1.39) | 1,400 | 1.21 (1.15, 1.28) | 558 | 1.04 (0.95, 1.13) | 947 | 1.79 (1.67, 1.92) |
| ^†^ All analyses were stratified by study area and 5-year age-at-risk group and adjusted for education and alcohol drinking. ^‡^ Only include those who had quit voluntarily (i.e. not due to ill health). ^*^ Include current smokers and ex-smokers who had quit due to ill health. For analyses of ischaemic heart disease and stroke types participants with a prior history of CVD reported at baseline were excluded. Likewise, for analyses of COPD and lung cancer, participants with a prior history of respiratory disease and cancer reported at baseline were excluded respectively. | | | | | | | | | | |

# eTable 11: Adjusted HRs for risks of any disease incidence and overall mortality associated with smoking with and without excluding those with prior cancer reported at baseline, in men

|  | **All disease incidence** | | | | **All-cause mortality** | | | |
| --- | --- | --- | --- | --- | --- | --- | --- | --- |
|  | **Prior cancer excluded** | | **Prior cancer not excluded** | | **Prior cancer excluded** | | **Prior cancer not excluded** | |
| **Smoking category** | **Number of events** | **HR (95% CI)^†^** | **Number of events** | **HR (95% CI)^†^** | **Number of events** | **HR (95% CI)^†^** | **Number of events** | **HR (95% CI)^†^** |
| Never-regular smoker | 33,267 | 1.00 (0.99, 1.01) | 33,472 | 1.00 (0.99, 1.01) | 5,856 | 1.00 (0.97, 1.03) | 5,951 | 1.00 (0.97, 1.03) |
| Ever-regular smoker | 104,358 | 1.09 (1.08, 1.11) | 104,981 | 1.09 (1.08, 1.11) | 21,490 | 1.33 (1.29, 1.37) | 21,818 | 1.33 (1.29, 1.37) |
| Ex-smoker (by choice)^‡^ | 9,665 | 1.05 (1.03, 1.07) | 9,725 | 1.05 (1.03, 1.07) | 1,957 | 1.06 (1.01, 1.10) | 1,984 | 1.05 (1.01, 1.10) |
| Regular smoker | 94,693 | 1.10 (1.09, 1.10) | 95,256 | 1.10 (1.09, 1.10) | 19,533 | 1.37 (1.35, 1.39) | 19,834 | 1.37 (1.35, 1.40) |
| Ex-smoker (ill health) | 10,573 | 1.24 (1.21, 1.26) | 10,883 | 1.25 (1.22, 1.27) | 3,245 | 1.57 (1.51, 1.62) | 3,429 | 1.61 (1.55, 1.66) |
| Current smoker | 84,120 | 1.08 (1.07, 1.09) | 84,373 | 1.08 (1.07, 1.09) | 16,288 | 1.33 (1.31, 1.36) | 16,405 | 1.33 (1.31, 1.35) |
| Age began smoking (years)^*^ |  |  |  |  |  |  |  |  |
| 25+ | 28,434 | 1.05 (1.04, 1.06) | 28,586 | 1.05 (1.04, 1.06) | 6,172 | 1.20 (1.17, 1.23) | 6,251 | 1.20 (1.17, 1.23) |
| 18-24 | 49,853 | 1.11 (1.10, 1.12) | 50,151 | 1.11 (1.10, 1.12) | 9,588 | 1.42 (1.39, 1.45) | 9,758 | 1.42 (1.40, 1.45) |
| <18 | 16,406 | 1.15 (1.14, 1.17) | 16,519 | 1.16 (1.14, 1.17) | 3,773 | 1.67 (1.62, 1.73) | 3,825 | 1.67 (1.61, 1.72) |
| No. smoked (cig/day)^*^ |  |  |  |  |  |  |  |  |
| <15 | 32,927 | 1.06 (1.05, 1.07) | 33,121 | 1.06 (1.05, 1.07) | 7,732 | 1.34 (1.30, 1.37) | 7,840 | 1.33 (1.30, 1.37) |
| 15-24 | 43,606 | 1.11 (1.09, 1.12) | 43,879 | 1.11 (1.10, 1.12) | 8,171 | 1.37 (1.34, 1.40) | 8,309 | 1.37 (1.34, 1.40) |
| 25+ | 18,160 | 1.16 (1.14, 1.17) | 18,256 | 1.16 (1.14, 1.17) | 3,630 | 1.50 (1.45, 1.55) | 3,685 | 1.50 (1.45, 1.55) |
| ^†^ All analyses were stratified by study area and 5-year age-at-risk group and adjusted for education and alcohol drinking. ^‡^ Only include those who had quit voluntarily (i.e. not due to ill health). ^*^ Include current smokers and ex-smokers who had quit due to ill health. | | | | | | | | |

# eTable 12: Adjusted HRs for risks of any disease incidence and overall mortality associated with smoking with and without excluding those with prior cancer reported at baseline, in women

|  | **All disease incidence** | | | | **All-cause mortality** | | | |
| --- | --- | --- | --- | --- | --- | --- | --- | --- |
|  | **Prior cancer excluded** | | **Prior cancer not excluded** | | **Prior cancer excluded** | | **Prior cancer not excluded** | |
| **Smoking category** | **Number of events** | **HR (95% CI)^†^** | **Number of events** | **HR (95% CI)^†^** | **Number of events** | **HR (95% CI)^†^** | **Number of events** | **HR (95% CI)^†^** |
| Never-regular smoker | 196,805 | 1.00 (0.99, 1.01) | 198,037 | 1.00 (0.99, 1.01) | 18,762 | 1.00 (0.97, 1.03) | 19,097 | 1.00 (0.97, 1.03) |
| Ever-regular smoker | 7,761 | 1.04 (1.01, 1.06) | 7,819 | 1.04 (1.01, 1.06) | 1,895 | 1.43 (1.36, 1.51) | 1,920 | 1.44 (1.36, 1.51) |
| Ex-smoker (by choice)^‡^ | 979 | 1.02 (0.96, 1.09) | 986 | 1.02 (0.96, 1.09) | 243 | 1.27 (1.12, 1.44) | 245 | 1.27 (1.12, 1.43) |
| Regular smoker | 6,782 | 1.04 (1.01, 1.06) | 6,833 | 1.04 (1.01, 1.06) | 1,652 | 1.46 (1.39, 1.53) | 1,675 | 1.47 (1.40, 1.54) |
| Ex-smoker (ill health) | 1,244 | 1.22 (1.15, 1.29) | 1,260 | 1.22 (1.15, 1.29) | 369 | 1.57 (1.41, 1.74) | 378 | 1.59 (1.43, 1.76) |
| Current smoker | 5,538 | 1.01 (0.98, 1.03) | 5,573 | 1.01 (0.98, 1.03) | 1,283 | 1.43 (1.36, 1.51) | 1,297 | 1.44 (1.36, 1.51) |
| Age began smoking (years)^*^ |  |  |  |  |  |  |  |  |
| 25+ | 3,428 | 1.02 (0.99, 1.06) | 3,456 | 1.02 (0.99, 1.06) | 757 | 1.37 (1.28, 1.47) | 772 | 1.38 (1.29, 1.48) |
| 18-24 | 1,988 | 1.05 (1.01, 1.10) | 1,999 | 1.05 (1.01, 1.10) | 473 | 1.44 (1.32, 1.58) | 477 | 1.44 (1.32, 1.58) |
| <18 | 1,366 | 1.05 (1.00, 1.11) | 1,378 | 1.06 (1.00, 1.12) | 422 | 1.71 (1.55, 1.89) | 426 | 1.71 (1.55, 1.89) |
| No. smoked (cig/day)^*^ |  |  |  |  |  |  |  |  |
| <15 | 5,135 | 1.02 (1.00, 1.05) | 5,168 | 1.02 (1.00, 1.05) | 1,231 | 1.40 (1.32, 1.48) | 1,244 | 1.40 (1.32, 1.48) |
| 15+ | 1,647 | 1.08 (1.03, 1.14) | 1,665 | 1.09 (1.04, 1.14) | 421 | 1.69 (1.54, 1.86) | 431 | 1.71 (1.55, 1.88) |
| ^†^ All analyses were stratified by study area and 5-year age-at-risk group and adjusted for education and alcohol drinking. ^‡^ Only include those who had quit voluntarily (i.e. not due to ill health). ^*^ Include current smokers and ex-smokers who had quit due to ill health. | | | | | | | | |

# eFigure 23: Kaplan-Meier curves for overall survival from age-at-risk of 35, among men and women


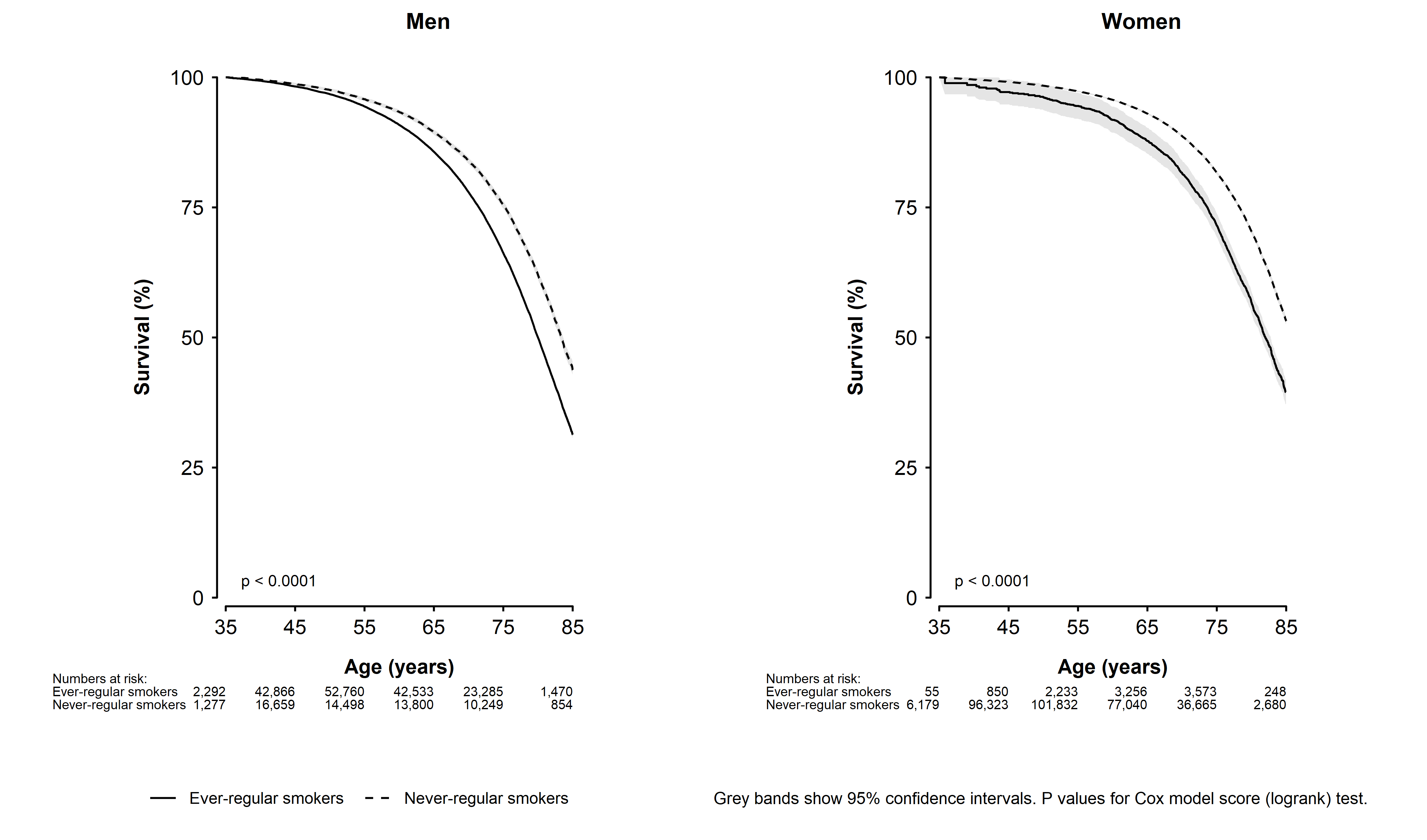


# eFigure 24: Total expected hospitalisations and days in hospital from any causes from age-at-risk of 35 years among men and women


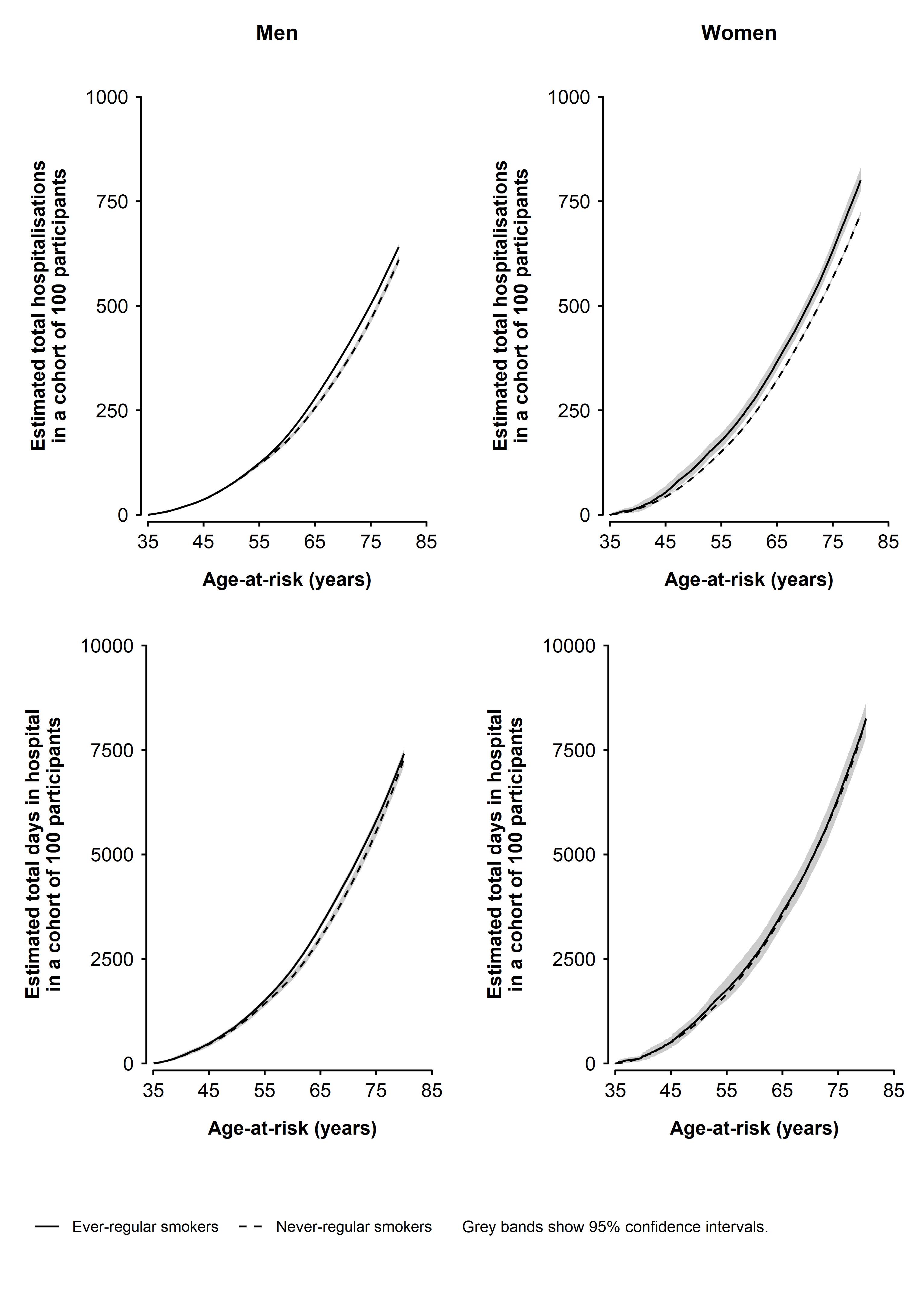


# eFigure 25: Total expected hospitalisations and days in hospital due to cancer, cardiovascular disease, respiratory disease and other conditions from age-at-risk of 35 years among men


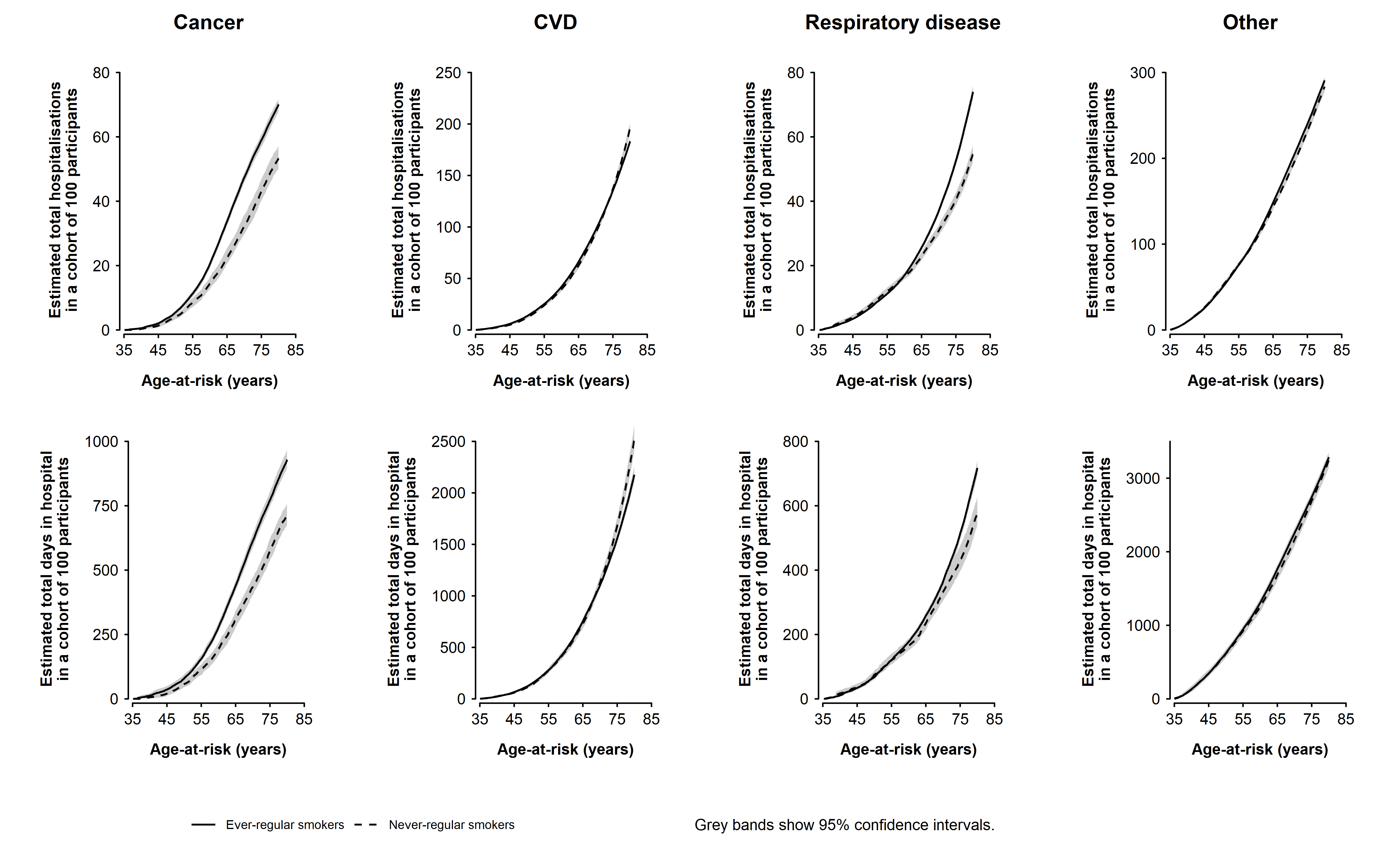


# eFigure 26: Adjusted HRs for all-cause mortality and all disease incidence by years stopped smoking and reason stopped, among men


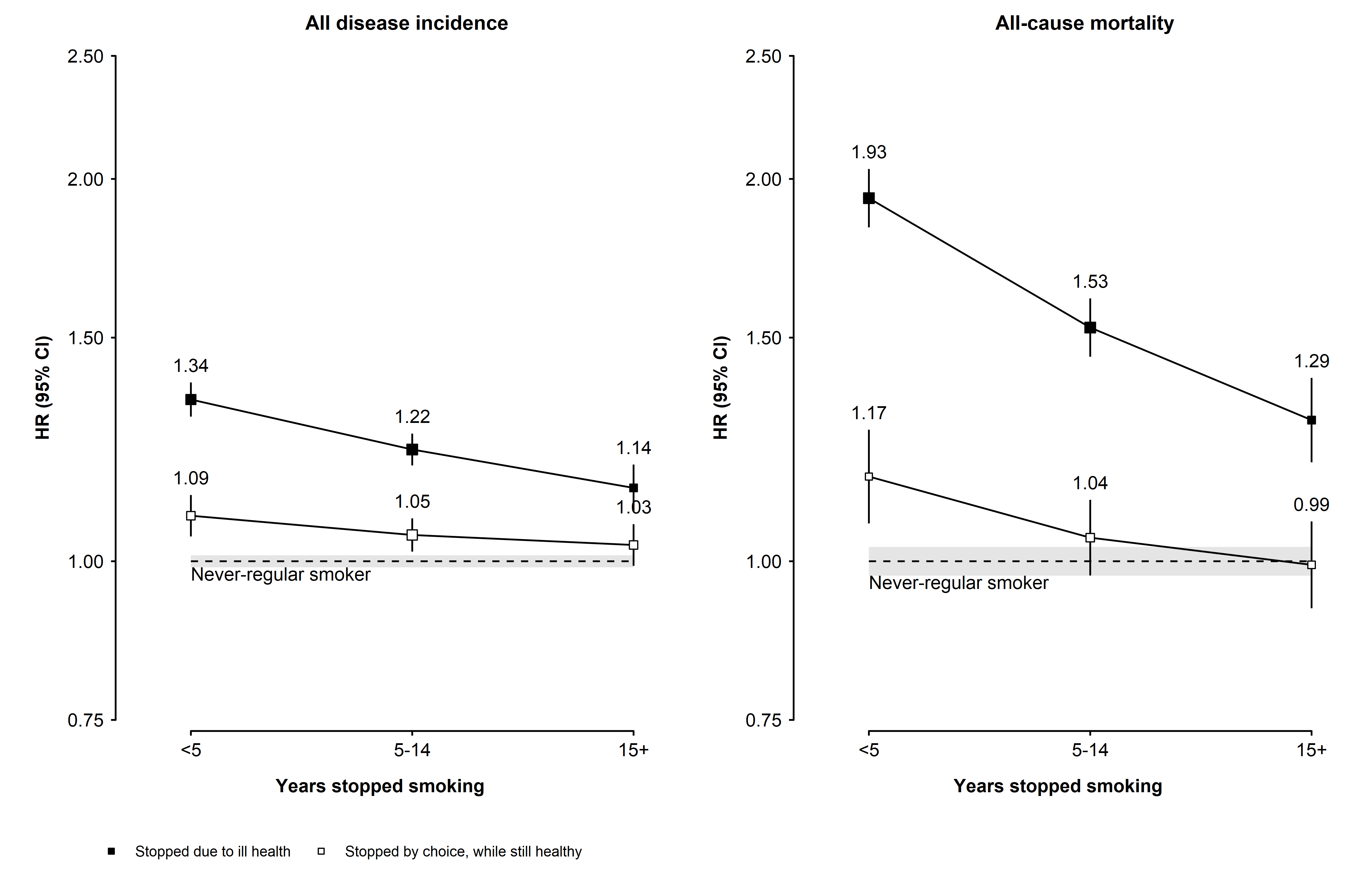


# eTable 13: Comparison of clinical conditions considered as likely causally related to smoking in the 2014 US Surgeon General Report and 2019 Global Burden of Disease Study†

| **2014 US Surgeon General report** | **Global Burden of Disease 2019 study** |
| --- | --- |
| **Cancer** |  |
| Bladder; cervical; colorectal, endometrial (postmenopausal), oesophageal, kidney, acute leukaemia, liver, lung, oral cavity and pharyngeal, pancreatic, stomach | Bladder, cervical, colorectal, endometrial, oesophageal, kidney, acute leukaemia, liver, lung, oral cavity and pharyngeal, pancreatic, stomach, larynx, breast, prostate, nasopharynx, other pharynx, acute lymphoid leukaemia, chronic lymphoid leukaemia, acute myeloid leukaemia, chronic myeloid leukaemia, other leukaemia |
| **CVD** |  |
| Abdominal aortic aneurysm, atherosclerosis, peripheral vascular disease, stroke, coronary heart disease | Atrial fibrillation & flutter, aortic aneurysm, peripheral artery disease, ischaemic heart disease, ischaemic stroke, intracerebral haemorrhage, subarachnoid haemorrhage |
| **Respiratory** |  |
| Chronic obstructive pulmonary disease, chronic respiratory symptoms (cough, phlegm, wheeze, dyspnea etc.), acute respiratory illnesses (including pneumonia); tuberculosis; lung function decline | Chronic obstructive pulmonary disease; lower respiratory infections; tuberculosis; asthma |
| **Other** |  |
| Periodontitis; diabetes mellitus; adverse surgical outcomes; nuclear cataracts; age-related macular degeneration (neovascular and atrophic forms); hip fractures; low bone density (in postmenopausal women); peptic ulcer (in people with H pylori infection) | Type II diabetes, peptic ulcer, gallbladder & biliary diseases; Alzheimer's disease and other dementia; Parkinson's disease (inverse association); multiple sclerosis; rheumatoid arthritis; low back pain; cataract; age-related macular degeneration; pedestrian road injuries; cyclist road injuries; motorcyclist road injuries; motor vehicle road injuries; other road injuries; other transport injuries; falls; other exposure to mechanical forces; non-venomous animal contact; physical violence by other means; |
| â€  Conditions in red are those only reported in either one of the two documents. | |

# eTable 14: Adjusted HRs for diseases significantly (after FDR adjustment) inversely associated with smoking, after further adjustment and after excluding first five years of follow-up

|  | | **Main analyses** | | **Additional adjustment^*^** | | **Exclude first 5 years of follow-up^†^** | |
| --- | --- | --- | --- | --- | --- | --- | --- |
|  |  | **Number of events** | **HR (95% CI)** | **Number of events** | **HR (95% CI)** | **Number of events** | **HR (95% CI)** |
| G20 | Parkinson disease | 838 | 0.73 (0.59, 0.89) | 838 | 0.73 (0.60, 0.89) | 636 | 0.69 (0.55, 0.86) |
| H11, H13 | Other disorders of conjunctiva | 3,161 | 0.75 (0.66, 0.85) | 3,161 | 0.75 (0.66, 0.85) | 2,050 | 0.70 (0.60, 0.82) |
| I83, I85, I86 | Varicose veins | 2,326 | 0.81 (0.72, 0.92) | 2,326 | 0.84 (0.74, 0.95) | 1,690 | 0.82 (0.71, 0.95) |
| J47 | Bronchiectasis | 1,592 | 0.82 (0.71, 0.96) | 1,592 | 0.75 (0.64, 0.87) | 1,152 | 0.70 (0.59, 0.84) |
| K40 | Inguinal hernia | 1,133 | 0.75 (0.65, 0.85) | 1,133 | 0.73 (0.64, 0.84) | 724 | 0.74 (0.63, 0.88) |
| M17 | Gonarthrosis [arthrosis of knee] | 1,945 | 0.74 (0.63, 0.87) | 1,945 | 0.79 (0.67, 0.93) | 1,697 | 0.80 (0.67, 0.95) |
| M19 | Other arthrosis | 16,794 | 0.91 (0.86, 0.96) | 16,794 | 0.93 (0.88, 0.99) | 7,127 | 0.91 (0.83, 0.98) |
| ^*^ Additionally adjusted for BMI, self rated health, and occupation. ^†^ Additionally adjusted and excluding first five years of follow-up. | | | | | | | |
